# Penicimenolides A-F, Resorcylic Acid Lactones from *Penicillium* sp., isolated from the Rhizosphere Soil of *Panax notoginseng*

**DOI:** 10.1038/srep27396

**Published:** 2016-06-08

**Authors:** Ya-Nan An, Xue Zhang, Tian-Yuan Zhang, Meng-Yue Zhang, Qian-Zhang, Xiao-Yu Deng, Feng Zhao, Ling-Juan Zhu, Guan Wang, Jie Zhang, Yi-Xuan Zhang, Bo Liu, Xin-Sheng Yao

**Affiliations:** 1 Shenyang Pharmaceutical University, Shenyang 110016, People’s Republic of China; 2 School of Pharmacy, Key Laboratory of Molecular Pharmacology and Drug Evaluation (Yantai University), Ministry of Education, Collaborative Innovation Center of Advanced Drug Delivery System and Biotech Drugs in Universities of Shandong, Yantai University, Yantai 264005, People’s Republic of China; 3 State Key Laboratory of Biotherapy and Cancer Center, West China Hospital, Sichuan University, and Collaborative Innovation Center of Biotherapy, Chengdu 610041, People’s Republic of China; 4 Institute of Traditional Chinese Medicine & Natural Products, College of Pharmacy, Jinan University, Guangzhou 510632, People’s Republic of China

## Abstract

Five new 12-membered resorcylic acid lactone derivatives, penicimenolides A-E (**1–5**), one new ring-opened resorcylic acid lactone derivative penicimenolide F (**6**), and six known biogenetically related derivatives (**7–12**) were isolated from the culture broth of a strain of *Penicillium* sp. (NO. SYP-F-7919), a fungus obtained from the rhizosphere soil of *Panax notoginseng* collected from the Yunnan province of China. Their structures were elucidated by extensive NMR analyses, a modified Mosher’s method, chemical derivatization and single crystal X-ray diffraction analysis. Compounds **2–4** exhibited potent cytotoxicity against the U937 and MCF-7 tumour cell lines and showed moderate cytotoxic activity against the SH-SY5Y and SW480 tumour cell lines. The substitution of an acetyloxy or 2-hydroxypropionyloxy group at C-7 significantly increased the cytotoxic activity of the resorcylic acid lactone derivatives. Subsequently, the possible mechanism of compound **2** against MCF-7 cells was preliminarily investigated by *in silico* analysis and experimental validation, indicating compound **2** may act as a potential MEK/ERK inhibitor. Moreover, proteomics analysis was performed to explore compound **2**-regulated concrete mechanism underlying MEK/ERK pathway, which is still need further study in the future. In addition, compounds **2–4** and **7** exhibited a significant inhibitory effect on NO production induced by LPS.

*Panax notoginseng* is widely distributed in Yunnan, Guangxi and Jiangxi province of China and its roots have been used as a traditional Chinese medicine for the treatment of hemorrhages, blood stasis and improvement of blood circulation and remission pain^1^. In recent years, the investigations regarding the bioactive secondary metabolites from endophytic or rhizospheric fungi of *Panax notoginseng* have been receiving increasing attention, leading to the various secondary metabolites with antimicrobial^2^,^3^, antifungal^4^, and cytotoxic activities^5^. During our studies for new natural bioactive constituents from rhizospheric fungus of *Panax notoginseng*, a strain of *Penicillium* sp. (SYP-F-7919) has drawn our interest because the EtOAc extract of the culture broth exhibited typical resorcylic acid lactones (RAL_s_) UV absorptions (λ_max_) at 215, 264 and 297 nm^6^.

RALs are a class of fungal polyketide derivatives that are produced by a variety of fungal strains, such as *Lasiodiplodia theobromae*^7^,^8^, *Penicillium* sp.^9^, *Syncephalastrum racemosum*^10^*, Pyrenophora teres*^11^ and *Acremonium zeae*^12^. Since the first RAL_12_, lasiodiplodin, was isolated in 1971^13^, approximately 30 natural RAL_12_ compounds had been obtained^6^,^7^,^8^,^9^,^10^,^11^,^12^,^13^,^14^,^15^,^16^,^17^,^18^,^19^,^20^,^21^,^22^,^23^,^24^,^25^,^26^, some of which exhibit antibacterial^15^,^16^, antifungal^12^,^14^, antitumour^10^,^17^,^18^, anti-inflammatory^19^,^20^,^23^, antidiabetic^21^, and antihypertensive activities^22^. In our study, five new RAL_12_ derivatives, penicimenolides **A-E** (**1–5**), a new ring-opened resorcylic acid lactone derivative penicimenolide **F** (**6**), and six known biogenetically related derivatives (**7–12**) (Fig. 1) were isolated from the culture broth of the fungus. In the cytotoxicity assay, compounds **2–4** showed potent cytotoxic activity against the U937 and MCF-7 tumour cell lines and displayed moderate cytotoxicity against the SH-SY5Y and SW480 tumour cell lines. Subsequently, the possible mechanism of compound **2** against MCF-7 cells was preliminarily investigated by *in silico* analysis and experimental validation, indicating compound **2** may act as a potential MEK/ERK inhibitor. Moreover, proteomics analysis was performed to explore compound **2**-regulated concrete mechanism underlying MEK/ERK pathway, which is still need further study in the future. In addition, compounds **2–4** and **7** exhibited a significant inhibitory effect on the production of nitric oxide (NO) in murine macrophages (RAW 264.7) activated by lipopolysaccharide (LPS). Herein, we report the isolation, structure elucidation, absolute configuration, bioactivities and preliminary mechanism of the compounds obtained from the *Penicillium* sp. SYP-F-7919.

## Results and Discussion

### Structural elucidation of resorcylic acid lactone derivatives

The ethyl acetate extract of the culture broth of the fungus *Penicillium* sp. was isolated by a combination of column chromatography, including silica gel, ODS, Sephadex LH-20, and reversed phase high performance liquid chromatography (HPLC) to yield twelve resorcylic acid lactone derivatives (**1–12**).

**Figure 2:**
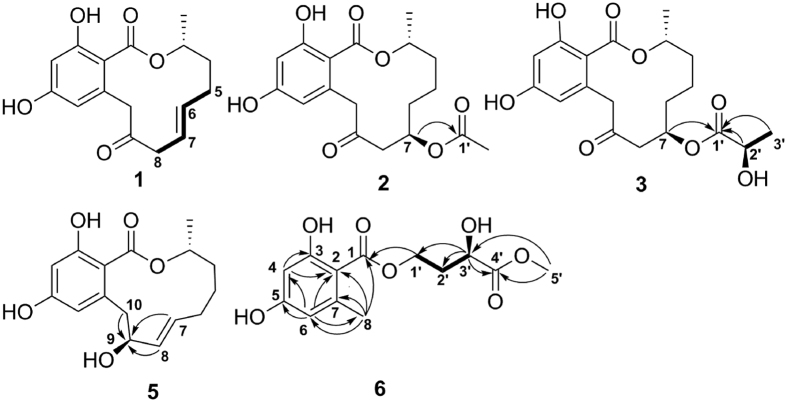
Key ^1^H-^1^H COSY (bold lines) and HMBC (arrows) correlations of 1–3, 5 and 6.

**Figure 3:**
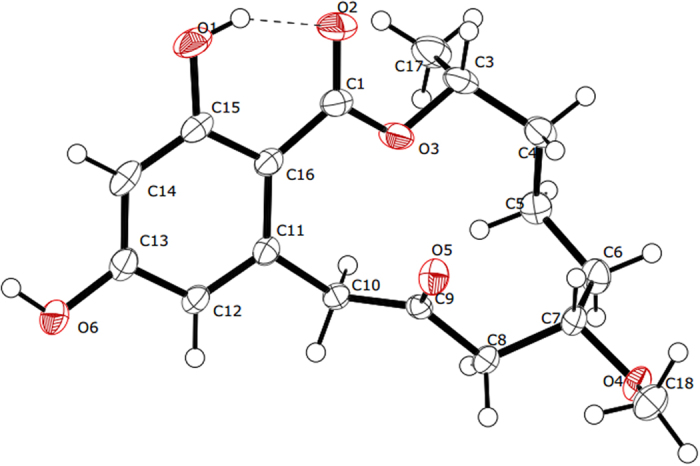
X-ray crystallographic structure of compound 10.

Penicimenolide A (**1**) was isolated as colourless needles, 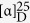 + 68.1 (*c* 0.5, MeOH). Its molecular formula was determined to be C_16_H_18_O_5_ by HRESIMS at *m/z* 291.1231 [M + H]^+^ (calcd. for C_16_H_19_O_5_, 291.1232). The IR spectrum of **1** revealed the presence of hydroxyl group(s) at 3384 cm^−1^, carbonyl group(s) at 1708 and 1642 cm^−1^ and an aromatic ring at 1605 and 1449 cm^−1^. A comparison of the ^1^H and ^13^C NMR spectroscopic data (Table 1) for **1** with those of **8** showed that the two compounds possessed a similar structure, except for the loss of two methylenes and the appearance of a pair of olefinic signals in **1**. The coupling constant (*J*_6,7_ = 15.5 Hz) indicated an *E* configuration for the double bond. The position of the double bond was confirmed by the ^1^H-^1^H COSY correlations of H-6/H-5 and H-7/H-8 (Fig. 2). Because the absolute configuration at C-3 in **10** was identified to be *R* based on the X-ray diffraction analysis (Cu Ka) (Fig. 3), the asymmetric carbon atom C-3 in the isolated compounds (except for **6**) was proposed to be an *R* configuration because of a shared biogenesis. For compound **1**, this conclusion was further confirmed by comparing the optical rotation value with **8** (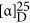 + 40.7). Based on the above evidence, the structure of **1** was identified to be (3*R*), (6*E*)-etheno-9-oxo-de-*O*-methyllasiodiplodin.

Penicimenolide B (**2**) was obtained as colourless needles, 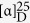 + 39.8 (*c* 0.5, MeOH). The molecular formula C_18_H_22_O_7_ was confirmed by HRESIMS at *m/z* 351.1435 [M + H]^+^ (calcd. for C_18_H_23_O_7_, 351.1444). Except for an additional acetyl group, the ^1^H and ^13^C NMR data (Table 1) for **2** were similar to those of **12**. The HMBC correlation between H-7 and the carbonyl carbon of the acetyl group at *δ*_C_ 172.2 suggested that 7-OH was acetylated (Fig. 2). To confirm the absolute configuration of **C-7** in **2**, both compounds **2** and **12** were reacted with acetic anhydride and pyridine. Because of the same retention time in the HPLC analysis and the similar ^1^H NMR data of the diacetylated derivative **2a** and the triacetylated derivative **12a** (see Supplementary Fig. S1), the absolute configuration of C-7 was confirmed to be *R*. Therefore, compound **2** was established to be (3*R*,7*R*)-7-acetoxyl-9-oxo-de-*O*-methyllasiodiplodin.

**Figure 4:**
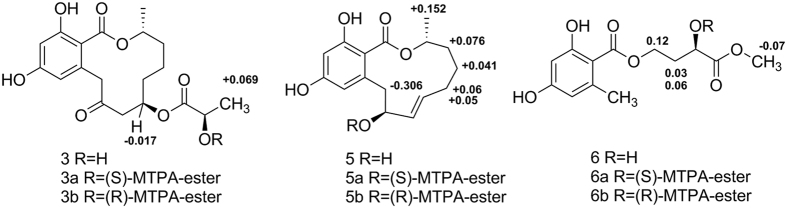
Δ*δ* Values (*δ*_a_-*δ*_b_ in ppm) obtained for the MTPA esters of compounds 3, 5 and 6.

Penicimenolide C (**3**) was obtained as yellow needles, 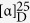
+15.4 (*c* 0.5, MeOH) and was determined to have a molecular formula of C_19_H_24_O_8_ on the basis of HRESIMS analysis (*m/z* 381.1549 [M + H]^+^, calcd. for C_19_H_25_O_8_, 381.1549). The ^1^H and ^13^C NMR data for **3** (Table 1) were similar to those of **2**, except for an additional oxygenated methine (*δ*_H_ 4.21, *δ*_C_ 68.1). The HMBC correlations from H-7 (*δ*_H_ 5.51), H-2′ (*δ*_H_ 4.21) and H_3_-3′ (*δ*_H_ 1.35) to C-1′ (*δ*_C_ 175.1), in combination with the ^1^H-^1^H COSY correlation between H_3_-3′ and H-2′, revealed the presence of a 2′-hydroxypropionyloxy fragment attached to C-7 (Fig. 2). Accordingly, a planar structure was proposed for **3**. The absolute configuration of C-2′ in **3** was established using the modified Mosher method^27^. Compound **3** was treated separately with (*R*)- and (*S*)-MTPA-Cl to obtained the respective (*S*)- and (*R*)-MTPA esters (**3a** and **3b**). The *R* configuration of C-2′ was determined by the positive Δ*δ*_H(S-R)_ value of H_3_-3´ and the negative Δ*δ*_H(S-R)_ value of H-7 (see Fig. 4 and Supplementary Table S2). In addition, the absolute configuration of C-7 was established by alkaline hydrolysis and HPLC analysis. Because of the same retention time of the alkaline hydrolysis product **3c** (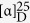 − 58.2) and the known compound **12** (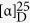 − 78.6), the configuration of C-7 was confirmed to be *R*. Based on the above evidence, the structure of **3** was determined to be (3*R*,7*R*,2′*R*)-7-(2′-hydroxypropionyloxy)-9-oxo-de-*O*-methyllasiodiplodin.

Penicimenolide D (**4**) was isolated as a yellowish oil, 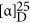 + 31.6 (*c* 0.5, MeOH). The HRESIMS of **4** (*m/z* 381.1548 ([M + H]^+^, calcd. for C_19_H_25_O_8_, 381.1549) showed the same molecular formula as **3**. The ^1^H and ^13^C NMR spectroscopic data for **4** (Table 2) were very similar to those for **3**. The analyses of the ^1^H-^1^H COSY, HSQC, and HMBC spectra of **4** indicated that the compound had an identical planar structure to that of **3**. A detailed comparison of the ^13^C NMR data for compounds **3** and **4** highlighted the differences in the chemical shifts at C-5, C-6, C-7 and C-8, suggesting that compound **4** is a C-7 epimer of **3**. Thus, the structure of **4** was determined to be (3*R*,7*S*,2′*R*)-7-(2′-hydroxypropionyloxy)-9-oxo-de-*O*-methyllasiodiplodin.

**Figure 5:**
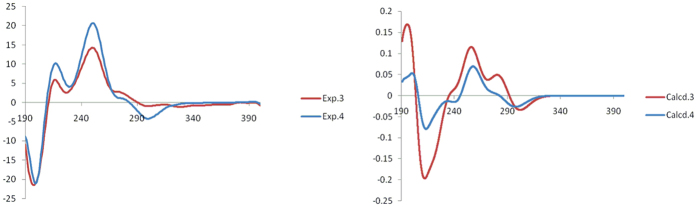
Calculated ECD spectra and experimental CD spectra of 3 and 4.

To compare computed electronic circular dichroism (ECD) with experimental results is a valid method to assign absolute configurations of natural products^28^,^29^,^30^,^31^,^32^. Thus, the absolute configurations of **3** and **4** were confirmed by quantum chemical TDDFT calculations of theirs ECD spectra. Conformational searches were performed using MMFF94S force field for **3** and **4**. All geometries (33 lowest energy conformers for **3** and 98 for **4**, respectively) with relative energy from 0-18 kcal/mol used in optimizations at the B3LYP/6-31G(d) level using Gaussian09 package^33^. The B3LYP/6-31G(d)-optimized conformers (32 lowest energy conformers for **3**) with relative energy from 0 to 10 kcal/mol and conformers (28 lowest energy conformers for **4**) with relative energy from 0 to 10 kcal/mol were then re-optimized at the B3LYP/6-311+G(d) level. ECD computations for all conformers were carried out at the B3LYP/6-311++G(2d,p) level in the gas phase. Boltzmann statistics were performed for ECD simulations with standard deviation of σ 0.22 eV. The computed ECD of **3** and **4** were looked similar to the experimental values of **3** and **4**, respectively (Fig. 5). Thus, the absolute configurations of **3** and **4** were further confirmed.

Penicimenolide E (**5**) was obtained as a yellowish oil, 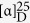 − 57.8 (*c* 0.25, MeOH). Its molecular formula was confirmed to be C_16_H_20_O_5_, which was deduced by the HRESIMS (*m/z* 293.1385 [M + H]^+^ calcd. for C_16_H_21_O_5_, 293.1389). The detailed analysis of the ^1^H and ^13^C NMR (Table 2) spectroscopic data revealed that **5** possessed a similar structure to *trans*-resorcylide^6^, and the only difference was the replacement of the ketone carbonyl at C-9 (*δ*_C_ 201.7) in *trans*-resorcylide by an oxygenated methine (*δ*_C_ 74.9) in **5**, which was further proven by the HMBC correlations from H-7, H-8, and H-10 to C-9 (Fig. 2). To confirm the absolute configuration of C-9 in **5**, a modified Mosher’s method^27^ was carried out to produce (*S*)- and (*R*)-MTPA esters (**5a** and **5b**). The negative Δ*δ*_H(S-R)_ value of H-10 and the positive Δ*δ*_H(S-R)_ value of H_3_-17, H-4, H-5 and H-6 unambiguously confirmed the *S* configuration of C-9 (see Fig. 4 and Supplementary Table S3). Based on the above evidence, the structure of compound **5** was confirmed to be (3*R*,9*S*)-(7*E*)-etheno-9-hydroxy-de-*O*-methyllasiodiplodin.

Penicimenolide F (**6**) was isolated as a yellowish oil, 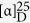 − 4.6 (*c* 0.25, MeOH). The HRESIMS exhibited a quasimolecular ion peak at *m/z* 285.0971 [M + H]^+^ (calcd. for C_13_H_17_O_7_, 285.0974), indicating a molecular formula of C_13_H_16_O_7_. The ^1^H NMR spectrum of **6** (Table 2) showed two meta-coupled aromatic protons at *δ*_H_ 6.15 (d, *J *= 2.4 Hz, H-4) and 6.19 (d, *J* = 2.4 Hz, H-6), two methyl signals at *δ*_H_ 3.68 (s, H_3_-5′) and 2.45 (s, H_3_-8), four methylene protons at *δ*_H_ 4.47 (m, H_2_-1′) and 2.06/2.24 (m, H-2′), and one methine proton at *δ*_H_ 4.32 (m, H-3′). The ^13^C NMR and DEPT spectra revealed the presence of 13 carbon resonances, including two ester carbonyls (*δ*_C_ 173.0, 176.1), six aromatic carbons, two methyls (one oxygenated), two methylenes (one oxygenated) and one oxygenated methine. The HMBC correlations (Fig. 2) from H-4 to C-1, C-2, C-3, C-5, and C-6, from H-6 to C-1, C-2, C-4, C-5, and C-8, and from H-8 to C-1, C-2, C-3, C-6, and C-7, combined with the molecular formula and the ^13^C NMR data, revealed the presence of a benzoyloxy fragment with a methyl group located at C-7 and two hydroxyl groups at C-3 and C-5, respectively. The ^1^H-^1^H COSY correlations between H-1′, H-3′ and H-2′, together with HMBC correlations from H_3_-5′ to C-3′ and C-4′ and from H-3′ to C-1′, C-2′, and C-4′, suggested the presence of a 3′-hydroxy-4′-oxo-4′-methyloxy-butyl fragment, which was attached to the benzoyloxy fragment, according to the correlation between H-1′ and C-1 in the HMBC spectrum. Additionally, the absolute configuration of C-3′ was established by a modified Mosher’s method^27^. The differences in the ^1^H-NMR chemical shifts between (*S*)- and (*R*)-MTPA esters (Δ*δ*_H(S-R)_) at H-1´, H-2´ and H_3_-5´ were analyzed to confirm the absolute configuration of C-3´ as *R* (see Fig. 4 and Supplementary Table S4). Thus, the structure of compound **6** was determined to be (3′*R*)-3′-hydroxy-4′-oxo-4′-methoxy-orsellinate.

In addition, six known compounds were isolated and identified as *cis*-resorcylide (**7**)^6^, dihydroresorcylide (**8**)^24^, (13*S*, 14*R*)-13-hydroxydihydroresorcylide (**9**)^25^, (11*S*)- methoxyresorcylide (**10**)^6^, (11*R*)-methoxyresorcylide (**11**)^6^ and 7-hydroxydihydroresorcylide (**12**)^6^ by a comparison of the observed and published data.

### Cytotoxicity of compounds against human tumour cell lines

Most of the isolated compounds (**2–5**, **7–12)** were evaluated for their cytotoxic activity against six human tumour cell lines, including U937, MCF-7, A549, SH-SY5Y, HepG2 and SW480 (Table 3). Taxol, a well-known anticancer drug, was used as a positive control. Compounds **2**-**4** exhibited potent cytotoxicity against the U937 and MCF-7 cell lines, with IC_50_ values ranging from 1.4 to 11.6 *μ*M and showed moderate cytotoxic activity against the SH-SY5Y and SW480 tumour cell lines. In contrast, compounds **5** and **7–12** were inactive against the six tested cell lines. The above data indicated that the substitution of an acetyloxy or a 2-hydroxypropionyloxy group at C-7 significantly increased the cytotoxic activity of the resorcylic acid lactone derivatives.

### Molecular docking of compound 2 with MEK1 and ERK1

Molecular docking is a widely used computational approach to screen potential active structures based on their interactions with the binding site^34^. The predicted results obtained from SEA showed that our candidate compounds potentially chose MEK1 or ERK1 as a target. In this study, compound **2** was selected as a potential MEK1-ERK1 inhibitor, based upon its favorable “-CDOCKER ENERGY” scores. In order to understand the conformation of compound **2** in the ATP binding site of MEK1 and ERK1, molecular docking analysis and MD simulations were conducted to evaluate the binding affinity of compound **2** and MEK1, as well as ERK1.

**Figure 6:**
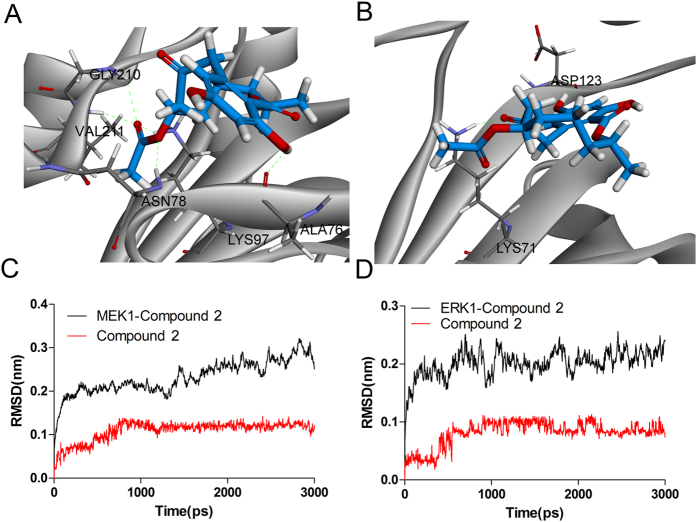
Binding analysis of compound 2 with the ATP binding domain of MEK1 and ERK1 proteins. (**A**) The binding model of compound **2** (colored red) with MEK1. Compound **2** and its hydrophobic surface in receptor residues were illustrated, from blue for hydrophilic to brown for hydrophobic, the important residues and their critical interactions (dash lines) with compound **2** in the binding site were depicted, conventional hydrogen bond, hydrophobic interactions were colored green and carnation, respectively. (**B**) The Binding model of compound **2** (colored marine) with ERK1. (**C**) RMSD of MEK1-compound **2** complex and compound **2** during the 3ns MD simulation. (**D**) RMSD of ERK1-compound **2** complex and compound **2** during the 3ns MD simulation.

After 3ns MD simulations, one frame (time of 3000ps) of the equalized trajectory was extracted, we found that compound **2** could bind to ATP binding domain of MEK1 and ERK1 stably. Compound **2** could form hydrogen bonds with residues Ala76, Asn78, Lys97, Gly210 and Val211 of the MEK1 (Fig. 6A). For ERK1, the hydrogen bonds were found between compound **2** and residue Lys71 and Asp123 of the ERK1, respectively (Fig. 6B). Moreover, we found compound **2** can form hydrophobic interactions in the hydrophobic pocket of MEK1 with lipophilic residue Val82. For ERK1 protein, the hydrophobic interactions were also formed with lipophilic residues Val56, Ala69, Met125 and Leu173. Altogether, these results suggest that compound **2** exhibits efficient binding to ATP binding domain of MEK1 and ERK1, which may support its inhibitory effect against MEK1 and ERK1.

Root mean square deviation (RMSD) fluctuation was an important criterion to gauge whether the protein-ligand system was stable. For MEK1-compound **2** system, RMSD of the complex were process about 2ns equilibrium during the whole simulation (Fig. 6C). During the process of MD simulation, RMSD of ERK1-compound **2** complex were limited to 0.3 nm, indicating a well-balanced system (Fig. 6D). Notably, we found RMSD of compound **2** in MEK1 protein remains stable after 800ps simulation, and then the RMSD fluctuations were narrow, indicating a stable binding in the ATP binding domain of MEK1 protein (Fig. 6C). The similar situation was also found in ERK1-compound **2** system, in which RMSD of compound **2** reached its dynamic equilibrium after 800ps simulation (Fig. 6D).

### Compound 2 induces apoptosis through MEK/ERK pathway in MCF-7 cells

**Figure 7:**
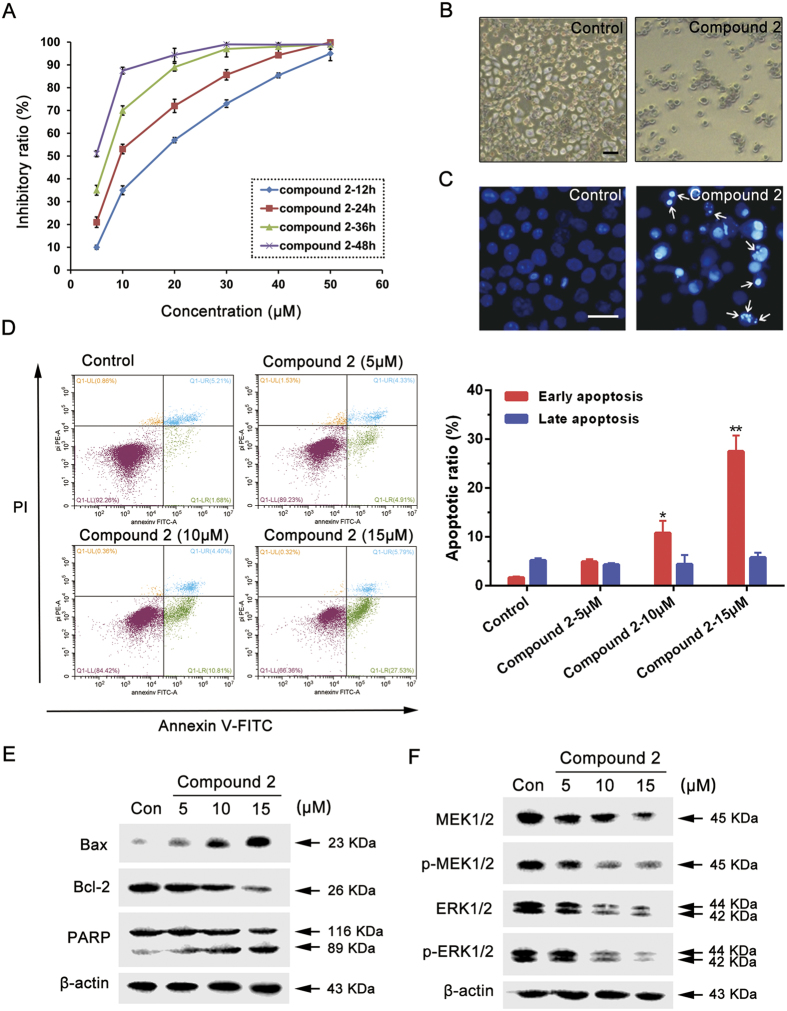
Compound 2 induces apoptosis in MCF-7 cells via targeting MEK/ERK pathway. (**A**) Effect of compound **2** on MCF-7 cell viability. The cells were cultured for 24 h, and then incubated with different concentrations of compound **2** for 12, 24, 36 and 48 h. The viability was determined by the MTT assay. The data are presented as the mean ± SD of the results from three independent experiments. (**B**) The cells were treated with compound **2** (10 μM) for 24 h, then observed by phase contrast microscope. Scale bar = 100 μm. (**C**) The cells were treated with compound **2** (10 μM) for 24 h then stained with Hoechst 33258 and observed by fluorescence microscope. Arrows indicate the apoptotic bodies. Scale bar = 50 μm. (**D**) The cells were treated with different concentrations of compound **2** for 24 h, then revealed by Annexin-V/PI double staining using flow cytometry analysis. *Compared with control, *p* < 0.05, **Compared with control, *p* < 0.01. (**E**) The cells were incubated with different concentrations of compound **2** for 24 h, then the expression levels of Bax, Bcl-2 and PARP were detected by western blot analysis. Each lane was loaded with 30 μg of whole cell extracts, β-actin was used as a loading control. (**F**) The cells were incubated with different concentrations of compound **2** for 24 h, then the expression levels of MEK1/2, p-MEK1/2, ERK1/2 and p-ERK1/2 were detected by western blot analysis. Each lane was loaded with 30 μg of whole cell extracts, β-actin was used as a loading control.

*In vitro* experiments were employed with MCF-7 cells to elucidate the possible mechanism of compound **2**, as ERK are often excessively activated in breast cancer^35^. Interestingly, we found that compound **2** demonstrated potent cytotoxic effect against MCF-7 cells in a time- and concentration-dependent manner, with an IC_50_ value of 9.893 μM (24 h) (Fig. 7A). In order to determine features of cell death induced by compound **2**, the morphologic changes of cells were examined by phase-contrast microscope. Compared with control group, compound **2** caused remarkable morphologic changes such as membrane blebbing and apoptotic bodies (Fig. 7B). These changes were further confirmed by Hoechst 33258 staining. The cells in control group showed uniform dispersion of low-density fluorescence, but compound **2** treated cells showed condensed, bright fluorescence and nuclear fragmentation (Fig. 7C). In addition, apoptotic ratio was further evaluated by Annexin-V/PI double staining. We found the early apoptotic cells ratio was increased from 1.68 ± 0.25% to 10.81 ± 2.47% (*p* < 0.05) and 27.53 ± 3.21% (*p* < 0.01), respectively, when treated with 10 μM and 15 μM of compound **2**, indicating that compound **2** increases apoptotic ratio in a dose-dependent manner (Fig. 7D).

To further demonstrate compound **2** induced apoptosis, we investigated the involvements of apoptotic markers in compound **2**-treated cells. We found that compound **2** significantly upregulated Bax expression as well as downregulated Bcl-2 expression. And the active form of PARP was also increased during compound **2** treatment (Fig. 7E). Based upon the molecular docking results of compound **2**, we next examined the expression levels of MEK1/2 and ERK1/2. As expected, the phosphorylation of MEK1/2 and ERK1/2 were simultaneously decreased after treatment of compound **2**, as well as the decreased expression of total MEK1/2 and ERK1/2, which is in accordance with our previous prediction (Fig. 7F). These results demonstrate that compound **2** could induce apoptosis by targeting MEK1/2 and ERK1/2 in MCF-7 cells.

### Proteomics-based analysis of potential apoptotic pathways

**Figure 8:**
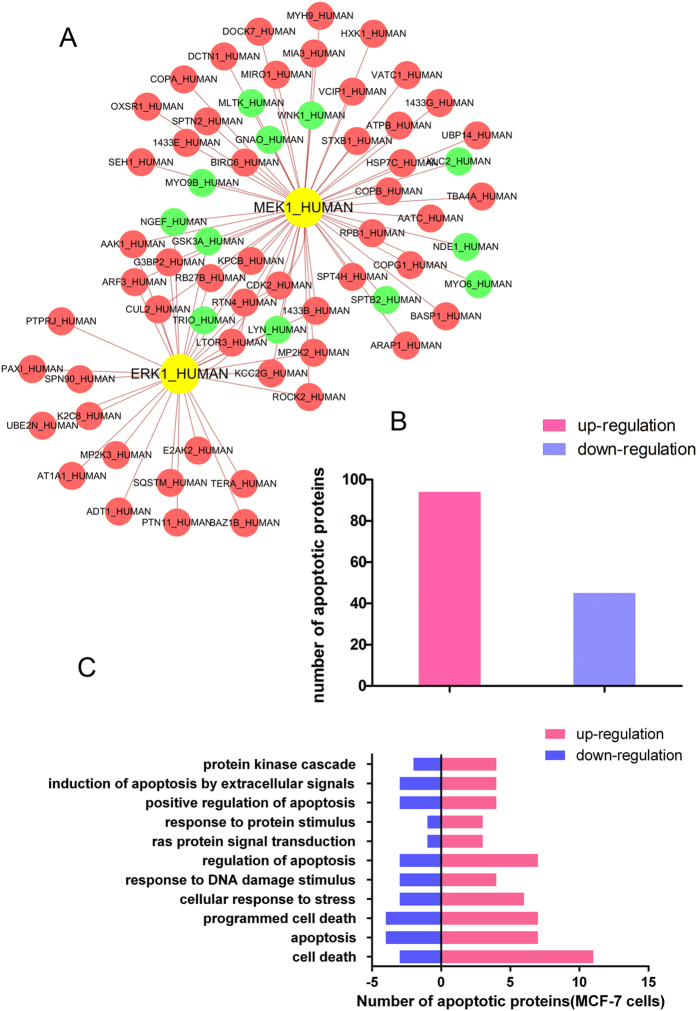
Proteomics analyses of compound 2-treated MCF-7 cells. (**A**) Proteomics-based identification of novel ERK1 or MEK1 apoptotic pathways in compound **2**-treated MCF-7 cells. (**B**) The difference of protein levels (upregulation or downregulation) between control and compound **2**-treated MCF-7 cells. (**C**) The apoptosis-related enrichment analyses of compound **2**-treated MCF-7 cells.

In order to study the potential molecular mechanisms of compound **2**-induced apoptosis, we used iTRAQ and MS/MS analysis to profile differentially expressed proteins in compound **2**-treated MCF-7. Based on high fold-change, we defined about thousands of differentially expressed proteins in compound **2**-treated MCF-7 cells. A proteomics-based MEK1/ERK1-modulated PPI network that includes 67 differentially expressed proteins and 84 interactions was constructed (Fig. 8A). In view of the above results, in breast cancer MEK1/ERK1-independent pathways may play a role in the process of apoptosis. Subsequently, differentially expressed proteins in proteomics-based MEK1/ERK1-modulated PPI network was annotated in two colors (Fig. 8B) and the 139 apoptosis-related proteins were divided into 11 clusters in MCF-7 cells (Fig. 8C). These results may shed a light on the exploration of further mechanism study in the future.

### Inhibitory effect of compounds on NO production

All of the isolated compounds, except for **1** and **6**, were evaluated for their inhibitory effect on NO production in LPS-activated murine macrophages (RAW 264.7), as shown in Table 4. In this assay, hydrocortisone was used as a positive control. The cell viability was measured in parallel using the MTT method to exclude the bioactivity resulting from the cytotoxicity of the tested compounds. Compounds **2-4** and **7** showed a significant inhibitory effect on NO production, with the IC_50_ values ranging from 0.7 to 5.8 *μ*M. In addition, compounds **5**, **8**, **10** and **12** had weak activity.

The free radical NO, which is produced by the oxidation of L-arginine catalyzed by a group of nitric oxide synthases (NOSs), can be involved in a series of physiological and pathological processes^35^. Many diseases, such as inflammation and cancer, have been reported to be associated with the overproduction of NO^36^. Therefore, an inhibitor of NO production may be beneficial to the treatment of diseases induced by excessive concentrations of NO, especially in some malignant tumours. And of course, the great significance and possible mechanisms of these active compounds, as well as the relationship between their antitumour activities and NO inhibition activities remain to be further explored.

## Methods

### General Experimental Procedures

Melting points were determined with a Tektronix X-4 micromelting-point apparatus (Beijing, China). The optical rotations were recorded on a JASCO P-1020 digital polarimeter (Kyoto, Japan), and the UV spectra were measured with a Shimadzu UV-2201 UV-Vis recording spectrophotometer (Kyoto, Japan). The IR data (KBr disks) were measured by a Bruker IFS-55 spectrometer (Rheinstetten, Germany). The CD spectra were obtained using a Bio-Logic MOS-450 (Grenoble, France). The 1D and 2D NMR spectra were run on Bruker AVANCE-400 or AVANCE-600 NMR spectrometers (Rheinstetten, Germany). The HRESIMS data were obtained on a Waters Synapt G2 QTOF mass spectrometer (MA, USA). The analytical HPLC was performed using an Agilent 1200 (CA, USA) equipped with a DAD detector using an ODS column (5 μm, 250 × 4.60 mm; Phenomenex Luna, CA, USA). Semipreparative HPLC was performed using a Shimadzu LC-6AD (Kyoto, Japan) equipped with a UV SPD-20A detector using an ODS column (5 μm, 250 × 10 mm, Phenomenex Luna, CA, USA). Column chromatography was carried out using silica gel (100–200 mesh and 200–300 mesh, Qingdao, China), ODS (60–80 μm, Tokyo, Japan), and Sephadex LH-20 (Uppsala, Sweden). Analytical grade solvents were used for the extraction and chromatographic separation.

### Fungal Material

The fungal strain used in this work was isolated from the rhizosphere soil of *Panax notoginseng*, which was collected from Wenshan, Yunnan province of China in November 2012. The fungus was identified on the basis of the DNA sequence of the internal transcribed spacer (ITSI-5.8S-ITS2 region). The sequence data derived from the fungal strain has been submitted and deposited in GeneBank with the accession number KU380346. BLAST search results revealed that the isolate belongs to the genus *Penicillium* and had a high sequence identity (99%) to the species *Penicillium menonorum*. A voucher specimen (No. SYP-F-7919) was deposited at the School of Life Science and Biopharmaceutics, Shenyang Pharmaceutical University.

The fungal strain was cultured on plates of potato dextrose agar (PDA) at 28 °C for 5 days. The plates were washed with double distilled water (ddH_2_O) to obtain conidial suspensions. To inoculate Erlenmeyer flasks (500 mL), 1 mL of conidial suspension was used, each containing 200 mL of the medium (which contained soluble starch 50 g, peanut powder 15 g, soybean flour 3 g, glucose 20 g, MgSO_4_ 0.75 g, KH_2_PO_4_ 1.0 g and distilled water 1 L). Each flask was incubated at 28 °C on a rotary shaker at 150 rpm for 7 days. Large scale fermentation was carried out in 200 Erlenmeyer flasks (500 mL).

### Extraction and Isolation

The fermentation broth (48 L) was centrifuged to separate the supernatant from the mycelia. The supernatant was extracted thoroughly with ethyl acetate (3 × 48 L), and the organic solvent was concentrated under reduced pressure to afford the crude extract (18.6 g), which was subjected to column chromatography over silica gel and eluted with cyclohexane-EtOAc (10:0 to 0:10) and EtOAc-MeOH (10:0 to 0:10) to produce eleven fractions (A-K). Fraction F (2.0 g), which was eluted with cyclohexane-EtOAc (8:2, v/v), was subjected to an ODS column and a MeOH-H_2_O stepwise gradient to obtain seven subfractions (F1-F7). Subfraction F5 (eluted with 60% MeOH, 325.4 mg) was further purified using a Sephadex LH-20 column that was eluted with CH_2_Cl_2_-MeOH (1:1). Then, it was purified by pHPLC using 55% MeOH to yield compounds **1** (4.5 mg, t_R_ 22.5 min), **7** (106.6 mg, t_R_ 25.3 min) and **8** (16.0 mg, t_R_ 36.2 min). Fraction G (eluted with cyclohexane-EtOAc, 7:3, 1.8 g) was applied to an ODS column that was eluted with a gradient of MeOH-H_2_O to give seven subfractions (G1-G7). Subfraction G3 (eluted 40% MeOH, 45.9 mg) was purified by pHPLC (25% MeCN) to afford compounds **6** (7.6 mg, t_R_ 36 min) and **5** (15.5 mg, t_R_ 40 min). Compounds **2** (8.4 mg, t_R_ 45 min), **10** (140.0 mg, t_R_ 26.5 min) and **11** (85.0 mg, t_R_ 22.5 min) were obtained from subfraction G4 (60% MeOH eluate, 649.2 mg) by purification with pHPLC using 55% MeOH. Fraction H (3.0 g) was also obtained from the cyclohexane-EtOAc (7:3, v/v) eluate on silica gel CC and then was separated into ten subfractions (H1-H10) by ODC CC using MeOH-H_2_O as the eluent. Subfractions H4 (40% MeOH eluate, 181.7 mg) and H5 (50% MeOH eluate, 106.0 mg) were purified by pHPLC (43% MeOH and 25% MeCN) to yield compounds **9** (10.5 mg, t_R_ 14.2 min) and **12** (15.5 mg, t_R_ 35 min), respectively. Compound **4** (10.0 mg, t_R_ 22.5 min) was purified from subfraction H6 (50% MeOH eluate, 162.6 mg) by pHPLC with 33% MeCN. Compound **3** (5.5 mg, t_R_ 40 min) was obtained from the subfraction H7 (60% MeOH eluate, 220.8 mg) using pHPLC (33% MeCN).

### Acetylation of 2 and 12

A sample of compound **2** (5.0 mg) was dissolved in dry pyridine (1.0 mL), and Ac_2_O (0.5 mL) was subsequently added. After stirring at 40 °C for 48 h, the mixture was concentrated in vacuo to dryness, and the residue was purified by pHPLC with MeCN-H_2_O (35:65, v/v) at a flow rate of 4 mL/min to yield **2a** (1.0 mg). Following the above procedure, compound **12** was acetylated to yield **12a**.

### Alkaline hydrolysis and HPLC analysis of 3

A mixture of compound **3** (5.0 mg) and 4% NaOH aqueous solution (5 mL) was stirred at 30–40 °C in a water bath for 3 h. The reaction mixture was neutralized with 10% HCl (4 mol/L) and extracted with EtOAc. After the extract was concentrated in vacuo to dryness, the residue was purified by pHPLC, eluting with MeCN-H_2_O (25:75, v/v) at a flow rate of 4 mL/min to obtain the hydrolyzate **3c** (1.0 mg). Then, **3c** was compared to **12** by HPLC analysis, which showed that the retention time of the two compounds were identical (see Supplementary Fig. S2).

#### Penicimenolide A (**1**)

colourless needles; mp 148–150 °C; 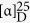 + 68.1 (*c* 0.5, MeOH); UV (MeOH) λ_max_ (log ε) 215 (3.88), 265 (3.54), 302 (3.27) nm; CD (1.4 × 10^−3^ M, MeOH) λ (Δε) 206 (-1.21), 217 (0.65), 290 (3.25), 330 (−0.05); IR (KBr) ν_max_ 3384, 2920, 1708, 1642, 1605, 1449, 1384, 1262 cm^−1^; ^1^H NMR and ^13^C NMR data, see Table 1; HR-ESI-MS: *m/z* 291.1231 ([M + H]^+^, calcd. for C_16_H_19_O_5_, 291.1232).

#### Penicimenolide B (**2**)

colourless needles; mp 128–130 °C; 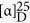 + 39.8 (*c* 0.5, MeOH); UV (MeOH) λ_max_ (log ε) 214 (4.03), 262 (3.74), 302 (3.47) nm; CD (1.4 × 10^−3^ M, MeOH) λ (Δε) 204 (−8.26), 224 (3.84), 260 (5.31), 306 (−1.01); IR (KBr) ν_max_ 3405, 2921, 1722, 1646, 1619, 1449, 1384, 1362, 1263 cm^−1^; ^1^H NMR and ^13^C NMR data, see Table 1; HR-ESI-MS: *m/z* 351.1435 ([M + H]^+^, calcd. for C_18_H_23_O_7_, 351.1444).

#### Penicimenolide C (**3**)

yellow needles; mp 118–119 °C; 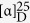 + 15.4 (*c* 0.5, MeOH); UV (MeOH) λ_max_ (log ε) 213 (4.05), 262 (3.80), 301 (3.56) nm; CD (1.3 × 10^−3^ M, MeOH) λ_max_ (Δε) 208 (−8.05), 225 (1.51), 261 (5.34), 308 (−0.34); IR (KBr) ν_max_ 3426, 2920, 1727, 1644, 1452, 1384, 1261, 1207 cm^−1^; ^1^H NMR and ^13^C NMR data, see Table 1; HR-ESI-MS: *m/z* 381.1549 ([M + H]^+^, calcd. for C_19_H_25_O_8_, 381.1549).

#### Penicimenolide D (**4**)

yellowish oil; 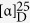 + 31.6 (*c* 0.5, MeOH); UV (MeOH) λ_max_ (log ε) 220 (4.31), 264 (4.01), 303 (3.85) nm; CD (5.3 × 10^−4^ M, MeOH) λ_max_ (Δε) 209 (−2.91), 223 (0.74), 261 (4.81), 302 (−1.61); IR (KBr) *ν*_max_ 3417, 2920, 1752, 1645, 1606, 1458, 1384, 1261, 1189; ^1^H NMR and ^13^C NMR data, see Table 2; HR-ESI-MS: *m/z* 381.1548 ([M + H]^+^, calcd. for C_19_H_25_O_8_, 381.1549).

#### Penicimenolide E (**5**)

yellowish oil; 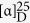 − 57.8 (*c* 0.25, MeOH); UV (MeOH) λ_max_ (log ε) 212 (3.94), 256 (3.27), 289 (3.07) nm; CD (6.8 × 10^−4^ M, MeOH) λ (Δε) 204 (4.65), 248 (−3.11), 285 (−0.70); IR (KBr) ν_max_ 3408, 2932, 1690, 1618, 1452, 1385, 1349, 1264 cm^−1^; ^1^H NMR and ^13^C NMR data, see Table 2; HR-ESI-MS: *m/z* 293.1385 ([M + H]^+^, calcd. for C_16_H_21_O_5_, 293.1389).

#### Penicimenolide F (**6**)

yellowish oil; 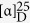
−4.6 (*c* 0.25, MeOH); UV (MeOH) λ_max_ (log ε) 220 (4.16), 264 (3.97), 302 (3.59) nm; CD (7.0 × 10^−4 ^M, MeOH) λ (Δε) 214 (5.49), 265 (−2.27), 298 (−2.18); IR (KBr) ν_max_ 3429, 2919, 1735, 1648, 1448, 1384, 1263 cm^−1^; ^1^H NMR and ^13^C NMR data, see Table 2; HR-ESI-MS: *m/z* 285.0971 ([M + H]^+^, calcd. for C_13_H_17_O_7_, 285.0974).

### Crystallography data for compound 10

Colourless lump crystals of **10** were obtained from CHCl_3_ containing a small amount of MeOH at room temperature. Crystallography data were obtained on an Agilent Super Nova (Dual, Cu at zero, Atlas) single crystal X-ray diffractometer with the Cu Ka (λ = 1.54184 Å). Structure solution and refinement were performed with the SHELXL program package. The crystallography data for **10**: C_17_H_22_O_6_; FW = 322.35; monoclinic space group P21, crystal size 0.40 × 0.40 × 0.30 mm^3^, unit cell dimensions a = 5.05804 (7) Å, b = 11.74745 (14) Å, c = 13.77837 (18) Å, α = γ = 90.00°, β = 100.1922 (13)°, *V* = 805.778 (18) Å^3^; Z = 2; *T* = 150 K; D_calcd_ = 1.329 mg/m^3^; μ = 0.836 mm^−1^, F (000) = 344, 5093 measured reflections were collected, 3141 independent reflections [Rint = 0.0167]. Completeness to θ_max_ was 98.58%; Final *R* indices: R_1_ = 0.0140 and wR_2_ was 0.1007 [I > 2σ (I)]. The goodness of fit on F^2^ was 0.971. Flack parameter = 0.08 (10).

The crystallography data for **10** were deposited in the Cambridge Crystallographic Data Centre (CCDC 1445050).

### Cytotoxicity assay

The U937 and MCF-7 human cancer cell lines were obtained through commercial purchasing from American Type Cultures Collection (ATCC, Manassas, VA, USA). The A549, SH-SY5Y, HepG2 and SW480 human cancer cell lines were purchased from Life Science College Cell Resource Center of Chinese Academy of Sciences (Shanghai, China). The MCF-7, A549, SH-SY5Y and HepG2 cells were cultured in DMEM medium with supplement of 10% fetal bovine serum (FBS, Hyclone, USA). The U937 and SW480 cells were cultured in RPMI-1640 medium with supplement of 10% fetal bovine serum (FBS, Hyclone, USA). All these cells were cultivated in a 5% CO_2_ humidified incubator at 37 °C. Cell lines in the phase of their growth cycle (6 × 10^4^ cells/mL) were added to each well of 96-well plate (100 μL/well) and cultivated for 24 h. The tested compounds (**2**–**5**, **7**–**12**) were serially diluted in DMEM/RPMI-1640 at different concentrations and placed into wells in a total volume of 200 μL. Taxol (National Institutes for Food and Drug Control, China) was used as a positive control. After 24 h, 20 μL of MTT (3-(4,5-dimethylthiazol-2-yl)-2,5-diphenyltetrazolium bromide; 5 mg/ml) was added to each well. After a 4 h incubation, the supernatant was removed from the wells and the formazan crystals were dissolved in DMSO (150 μL). The plates were then shaken for 15 min, and the absorbance was measured at 570 nm using an enzyme immunoassay instrument (Thermo Scientific, USA).

### Molecular docking

Target proteins of the small molecule compounds, were obtained from the similarity ensemble approach (SEA) (http://sea.bkslab.org/)^37^. We downloaded the initial three-dimensional geometric coordinates of the X-ray crystal structure of MEK1 (PDB ID: 3W8Q) and ERK1 (PDB ID: 4QTB). Accelrys Discovery Studio version 3.5 (Accelrys Inc., USA) with CHARMm force field parameters were used to dock the pre-generated conformations of compound into MEK1 and ERK1, respectively. The CDOCKER to a rigid receptor with grid-based scoring was performed. By this way, the ligands were allowed to be flexible and structurally rearranged in response to the receptors. Finally, according to the “-CDOCKER INTERACTION ENERGY” and “-CDOCKER ENERGY” scores, the top ten ranked conformations for each ligand were finally preserved for further analysis.

### Molecular dynamics (MD) simulations

To further verify the binding model of compound **2** with MEK1 and ERK1. MD simulations were carried out by GROMACS package (version 5.1.2)^38^. Protein and small molecule compound were charged using amber99sb-ildn force field and general AMBER force field (gaff), respectively^39^. The complex was solvated in a cubic box filled with TIP3P water molecules, the minimum distance from closest atom of solutes to edge of the box was define as 0.1 nm. Periodic boundary conditions (PBC) were used to avoid edge effects during the whole MD simulation. The charges of whole system were neutralized by adding an appropriate number Na^+^ and Cl^−^, so as to mimic a physiology concentration NaCl (0.15 M). Prior to operate the MD simulation, energy minimization was carried out to clear bad contacts. Then, 100ps NVT and 100ps NPT ensembles with simultaneous protein-ligand position restraints were also carried out to equilibrate the system. Consequently, 3ns MD production simulation was performed with a 2fs time step under constant temperature (300 K) and pressure (1 atm).

### Hoechst 33258 staining

MCF-7 cells were seeded in each well of 6-well culture plates at a density of 2.5 × 10^5^ cells/mL treated or untreated with compound **2** and cultured for 24 h. The subcellular structure alterations of apoptosis were observed under fluorescence inversion microscope.

### Annexin-V/PI double staining

MCF-7 cells were seeded into 6-well culture plates with different concentrations of compound **2** and cultured for 24 h. The collected cells were then fixed with 500 μL PBS. The apoptotic ratio was measured by flow cytometry (Becton Dickinson, Franklin Lakes, NJ) after employing Annexin-V-FLUOS Staining Kit (Roche, Germany).

### Western blot analysis

MCF-7 cells were cultured in 6-well culture plates for 24 h. Then cells were treated with different concentrations of compound **2**. All the cells were collected and were lysed by lysis buffer at 4 ^o^C for 1 h. After 12, 000 rpm centrifugation for 15 min, the protein level of the supernatant was quantified by Bio-Rad DC protein assay (Bio-Rad Laboratories, Hercules, CA, USA). Equal amounts of the total protein were separated by 12% SDS-PAGE and electrophoretically transferred to nitrocellulose membranes. Subsequently, membranes were blocked with 5% nonfat dried milk. Proteins were detected using primary antibodies (Danvers, MA, USA), followed by HRP-conjugated secondary antibody and visualized by employing ECL as the HRP substrate.

### iTRAQ and MS/MS analysis

The iTRAQ and MS/MS analysis were performed in compound **2**-treated MCF-7 cells. In simple terms, cells were dissolved in lysis buffer containing protease inhibitor (Sigma). Centrifuging the lysate for 1 h at 15 °C to get the supernatant, and stored it at −80 °C for further use. Protein quantitation was performed using Bio-Rad DC protein assay Kit (Bio-Rad). iTRAQ labeling was carried out using iTRAQ Reagent 4-Plex kit (AB SCIEX) based on the manufacturer’s protocol with minor modifications. The whole cell lysates were labeled with iTRAQ labeling reagent 114 and 115 for the pair of control MCF-7 and compound **2**-treated MCF-7, respectively. After 2D LC analysis and tandem mass spectrometry analysis, protein identification and relative iTRAQ quantification were performed with ProteinPilot™ Software 4.2 (AB SCIEX) using the Paragon™ algorithm for the peptide identification, which was further processed by Pro GroupTM algorithm where isoform-specific quantification was adopted to trace the differences between expressions of various isoforms. Results with iTRAQ ratio cutoff values of 1.2 and 0.8 for fold-change and number cutoff values of 3 for quantifiable peptides for in protein abundance were accepted. Moreover, the results of 114 and 115 were adopted when their expression level were changed at the same trend.

### Proteomics-based network construction of compound 2

To build the MEK1/ERK1 kinase protein–protein interaction (PPI) network, we collected diverse sets of biological evidence from seven online databases. Different PPIs were collected from the Database of Interacting Proteins (DIP)^40^, Biomolecular Object Network Databank (BOND)^41^, Human Protein Reference Database (HPRD)^42^, HomoMINT^43^, IntAct^44^, BioGRID^45^ and PrePPI^46^. Moreover, we extracted the MEK1/ERK1 subnetwork from the PPI network based on the ITRAQ-based proteomics results.

### Functional enrichment analyses

Differentially expressed proteins were assigned to 16 functional synaptic protein groups. Enrichment was only considered relevant when over represented functional groups contained at least 4 proteins. In addition, functional enrichment was determined using the DAVID functional annotation tool (http://david.abcc.ncifcrf.gov/). The functional categories used were GO term related to Biological Process (BP). And, the apoptosis-related proteins were detected and used as the background set.

### NO production inhibition assay

Mouse monocyte-macrophages (RAW 264.7) were maintained in RPMI 1640 medium supplemented with penicillin (100 U/mL), streptomycin (100 μg/mL) and heat-inactivated fetal bovine serum (10%) at 37 °C in a 5% CO_2_ humidified incubator. The inhibition of NO production was evaluated by measuring the accumulation of nitrite (NO_2_^−^) using a previously reported method^47^,^48^. The RAW 264.7 cells were seeded in 96-well plates (200 μL/well) at the initial density of 5 × 10^5^ cells/mL in RPMI 1640 medium. After incubation for 1 h, the tested samples at various concentrations and LPS (Sigma, 1 μg/mL) were simultaneously added to the wells. After being incubated at 37 °C for 24 h, the culture supernatant (100 μL/well) was transferred to the enzyme-labelled plates and then mixed with an equal volume of Griess reagent. After 10 min at room temperature, the absorbance of the reaction products was measured at 540 nm. The cell culture without the tested compounds was used as the LPS group, the cell culture sample without the tested compounds and LPS was used as control group, and hydrocortisone was used as a positive control. All tests were performed in quadruplicate. The concentration of NO_2_^−^ was calculated using a working curve of 1, 5, 10, 50 μM NaNO_2_ solutions, and the inhibitory rate on NO production was calculated by the level of NO_2_^−^. Cytotoxicity was measured using a 3-(4,5-dimethylthiazol-2-yl)-2,5-diphenyltetrazolium bromide (MTT) colorimetric assay after 24 h of incubation with the tested compounds.

## Additional Information

**How to cite this article**: An, Y.-N. *et al*. Penicimenolides A-F, Resorcylic Acid Lactones from *Penicillium* sp., isolated from the Rhizosphere Soil of *Panax notoginseng*. *Sci. Rep.* **6**, 27396; doi: 10.1038/srep27396 (2016).

## Supplementary Material

Supplementary Information

## Figures and Tables

**Figure 1:**
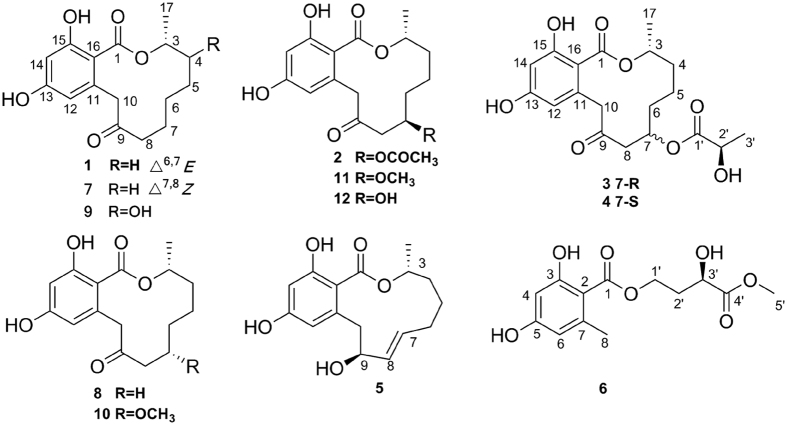
Chemical structures of compounds 1–12.

**Table 1:** ^1^H (600 MHz) and ^13^C NMR (150 MHz) data of 1–3 in CD_3_OD (*J* in Hz).

NO.	1		2		3	
	δ_C_	δ_H_	δ_C_	δ_H_	δ_C_	δ_H_
1	172.3 (C)		172.4 (C)		172.4 (C)	
3	74.8 (CH)	5.37 m	74.1 (CH)	5.16 m	74.1 (CH)	5.16 m
4	34.7 (CH_2_)	1.96 m;	34.0 (CH_2_)	1.65 m;	33.9 (CH_2_)	1.68 m;
		1.85 m		1.71 m		1.71 m
5	29.7 (CH_2_)	2.42 m;	19.2 (CH_2_)	1.56 m;	19.2 (CH_2_)	1.57 m;
		2.21 m		1.58 m		1.59 m
6	138.5 (CH)	5.60 dt (15.5, 6.9)	33.5 (CH_2_)	1.88 m;	33.4 (CH_2_)	1.89 m;
				1.56 m		1.61 m
7	122.5 (CH)	5.52 dt (15.5, 6.7)	71.6 (CH)	5.45 m	72.1 (CH)	5.51 m
8	46.2 (CH_2_)	2.99 m;	47.9 (CH_2_)	3.09 dd (15.9, 10.4);	47.7 (CH_2_)	3.14 dd (15.9, 10.4);
		2.99 m		2.68 dd (15.9, 1.4)		2.70 dd (15.9, 1.3)
9	208.2 (C)		206.7 (C)		206.6 (C)	
10	47.4 (CH_2_)	4.37 d (17.0);	52.9 (CH_2_)	4.55 d (18.7);	53.0 (CH_2_)	4.56 d (18.8);
		3.97 d (17.0)		3.85 d (18.7)		3.85 d (18.8)
11	139.1 (C)		139.9 (C)		139.9 (C)	
12	113.7 (CH)	6.12 d (2.5)	113.9 (CH)	6.11 d (2.5)	113.9 (CH)	6.11 d (2.4)
13	163.2 (C)		164.1 (C)		164.1 (C)	
14	103.1 (CH)	6.23 d (2.5)	103.2 (CH)	6.24 d (2.5)	103.2 (CH)	6.25 d (2.4)
15	165.5 (C)		167.0 (C)		167.0 (C)	
16	107.9 (C)		106.5 (C)		106.5 (C)	
17	19.3 (CH_3_)	1.34 d (6.5)	18.9 (CH_3_)	1.28 d (6.4)	18.9 (CH_3_)	1.29 d (6.4)
1′			172.2 (C)		175.1 (C)	
2′			21.3 (CH_3_)	2.01 s	68.1 (CH)	4.21 dd (13.8, 6.9)
3′					20.7 (CH_3_)	1.35 d (6.9)

**Table 2:** ^1^H (600 MHz) and ^13^C NMR (150 MHz) data of 4–6 in CD_3_OD (*J* in Hz).

NO.	4		5		6	
	δ_C_	δ_H_	δ_C_	δ_H_	δ_C_	δ_H_
1	172.6 (C)		170.8 (C)		173.0 (C)	
2					105.9 (C)	
3	76.1 (CH)	4.96 m	73.0 (CH)	5.08 m	166.4 (C)	
4	33.8 (CH_2_)	1.85 m;	33.4 (CH_2_)	1.93 m;	101.9 (CH)	6.15 d (2.4)
		1.76 m		1.66 m		
5	21.6 (CH_2_)	1.66 m;	22.5 (CH_2_)	1.70 m;	164.1 (C)	
		1.42 m		1.60 m		
6	34.0 (CH_2_)	1.74 m;	31.4 (CH_2_)	2.15 m;	112.7 (CH)	6.19 d (2.4)
		1.69 m		1.99 m		
7	70.8 (CH)	5.52 m	129.8 (CH)	5.49 dt (15.7, 6.3)	144.6 (C)	
8	50.1 (CH_2_)	2.99 dd (13.4, 3.0);	133.4 (CH)	5.35 dt (15.7, 5.2)	24.6 (CH_3_)	2.45 s
		2.63 dd (13.4, 10.3)				
9	207.3 (C)		74.9 (CH)	4.05 m		
10	51.0 (CH_2_)	4.77 d (18.5);	43.6 (CH_2_)	3.11 d (18.8);		
		3.77 d (18.5)		2.82 d (18.8)		
11	139.6 (C)		140.8 (C)			
12	114.1 (CH)	6.13 d (2.5)	111.1 (CH)	6.25 d (2.4)		
13	163.9 (C)		161.2 (C)			
14	103.1 (CH)	6.25 d (2.5)	102.3 (CH)	6.19 d (2.4)		
15	166.4 (C)		ND^a^			
16	106.9 (C)		115.1 (C)			
17	21.2 (CH_3_)	1.32 d (6.1)	19.0 (CH_3_)	1.31 d (6.4)		
1′	176.0 (C)				62.4 (CH_2_)	4.47 m
2′	68.1 (CH)	4.25 dd (13.8, 6.9)			34.4 (CH_2_)	2.24 m;
						2.06 m
3′	20.6 (CH_3_)	1.37 d (6.9)			68.9 (CH)	4.32 m
4′					176.1 (C)	
5′					52.7 (CH_3_)	3.68 s

^a^Designate signal not detected.

**Table 3:** Cytotoxicity of compounds 2–4 against human tumour cell lines.

Compound	IC_50_ (*μ*M)					
	MCF-7	U937	SH-SY5Y	HepG2	SW480	A549
**2**	9.89 ± 0.41	1.38 ± 0.18	44.41 ± 1.42	>100	22.66 ± 1.22	>100
**3**	11.59 ± 0.23	6.49 ± 0.41	47.62 ± 0.81	>100	24.82 ± 1.85	>100
**4**	10.47 ± 0.44	2.19 ± 0.074	73.47 ± 1.62	>100	41.18 ± 2.3	>100
Taxol	0.0152	0.0553	0.454	1.562	1.845	1.512

**Table 4:** Inhibitory effect of compounds 2–5 and 7–12 on NO production.

Compound	IC_50_ (*μ*M)
**2**	5.53 ± 0.39
**3**	5.79 ± 0.46
**4**	1.23 ± 0.10
**5**	68.39 ± 4.51
**7**	0.73 ± 0.06
**8**	73.93 ± 7.15
**9**	>100
**10**	70.45 ± 6.62
**11**	>100
**12**	55.94 ± 3.22
Hydrocortisone	48.66 ± 3.26
