# Supplementary material for: Penicimenolides A-F, Resorcylic Acid Lactones from Penicillium sp., isolated from the Rhizosphere Soil of Panax notoginseng
Source: Sci Rep. 2016 Jun 8;6:27396. doi: 10.1038/srep27396 (PMC4897632; doi:10.1038/srep27396)

Supplementary Information

Penicimenolides A-F, Resorcylic Acid Lactones from *Penicillium* sp., isolated from the Rhizosphere Soil of *Panax notoginseng*

Ya-Nan An[1+], Xue Zhang[1+], Tian-Yuan Zhang[1], Meng-Yue Zhang[1], Qian-Zhang[2], Xiao-Yu Deng[1], Feng Zhao[2], Ling-Juan Zhu[1], Guan Wang[1], Jie Zhang[1], Yi-Xuan Zhang[1*], Bo Liu[3*], and Xin-Sheng Yao[1,4*]

[1]Shenyang Pharmaceutical University, Shenyang 110016, People's Republic of China

[2]School of Pharmacy, Key Laboratory of Molecular Pharmacology and Drug Evaluation (Yantai University), Ministry of Education, Collaborative Innovation Center of Advanced Drug Delivery System and Biotech Drugs in Universities of Shandong, Yantai University, Yantai 264005, People's Republic of China

[3]State Key Laboratory of Biotherapy and Cancer Center, West China Hospital, Sichuan University, and Collaborative Innovation Center of Biotherapy, Chengdu 610041, People's Republic of China

[4]Institute of Traditional Chinese Medicine & Natural Products, College of Pharmacy, Jinan University, Guangzhou 510632, People's Republic of China

[+]These authors contributed equally to this work.

Correspondence and requests for materials should be addressed to X.S.Yao (E-mail: tyaoxs@jnu.edu.cn), Y.X.Zhang (zhangyxzsh@163.com) or B. Liu (liubo2400@scu.edu.cn)

List of Supporting Information

1. Acetylation of **2** and **12**..2
2. Alkaline hydrolysis of **3**..3
3. NMR spectra of compound **1-6**..3
4. NMR data of compound **8**..33
5. ^{1}H-NMR chemical shift differences for the MTPA esters of **3**,**5** and **6**..........................34
6. Single crystal X-ray diffraction analysis of compound **10**..35
7. CheckCIFPLATON report of compound **10**..50

1. Acetylation of 2 and 12.

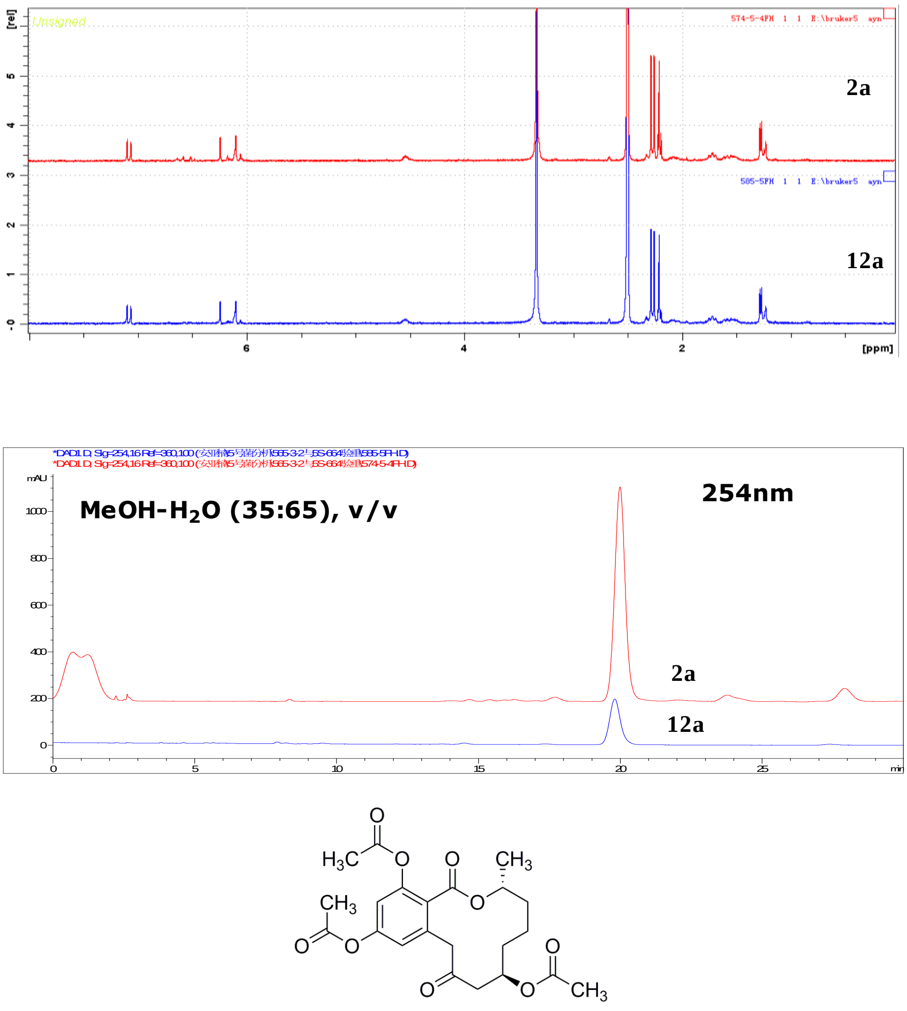

Figure S1 The ^{1}H-NMR spectra, HPLC analysis and structures of **2a** and **12a**

2.Alkaline hydrolysis of 3.

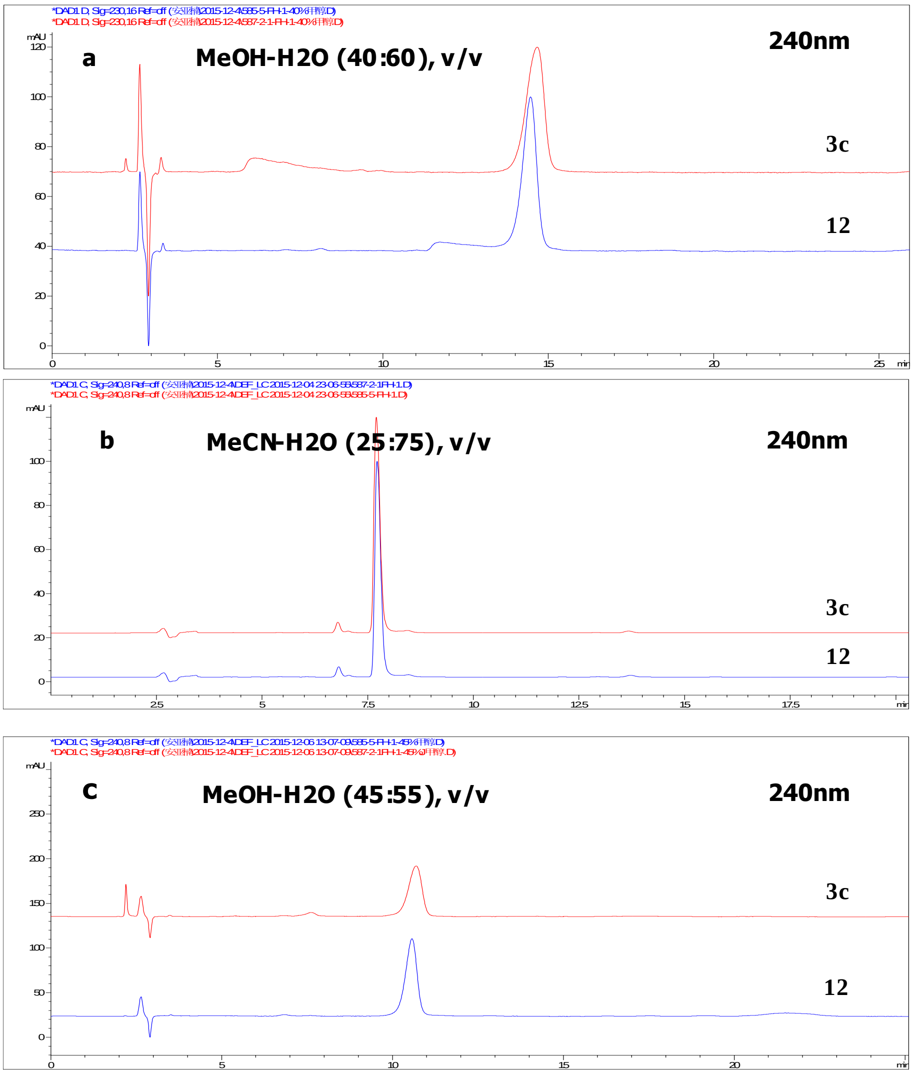

Figure S2 The alkaline hydrolysis product **3c** was compared with **12** by HPLC with three eluting systems (a: MeOH–H_2O (40:60, v/v), at a flow rate of 1 mL/min; b: MeOH–H_2O (45:55, v/v), at a flow rate of 1 mL/min; c: MeCN–H_2O (25:75, v/v), at a flow rate of 1 mL/min)

3. The spectra of compounds 1-6.

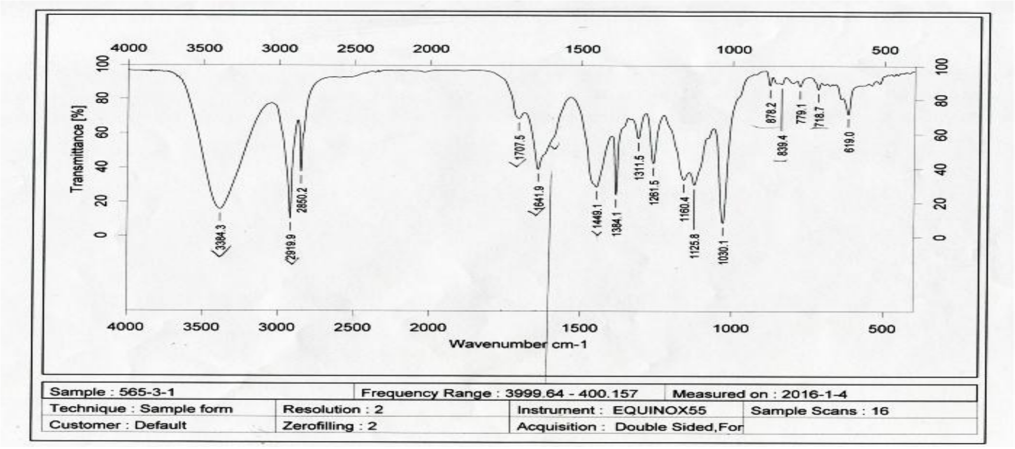

Figure S3 IR (KBr disc) spectrum of penicimenolide A (**1**)

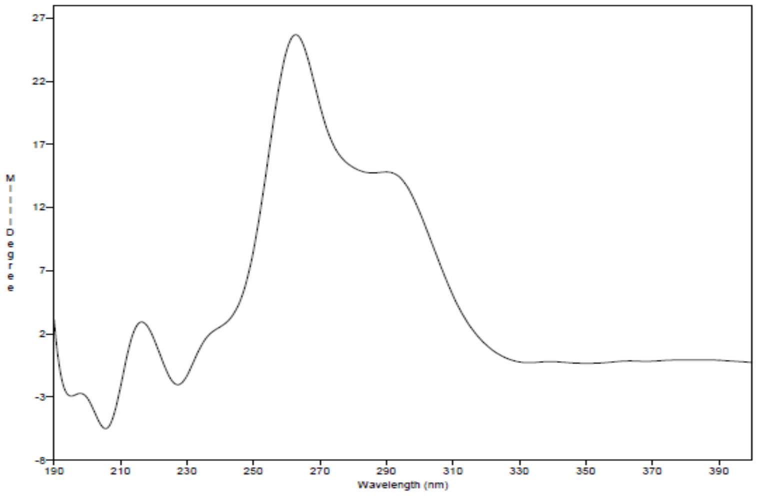

Figure S4 CD spectrum of penicimenolide A (**1**) in MeOH

Spectrum Peak Pick Report

FIELD FIELD TEXT

Data Set: 没有

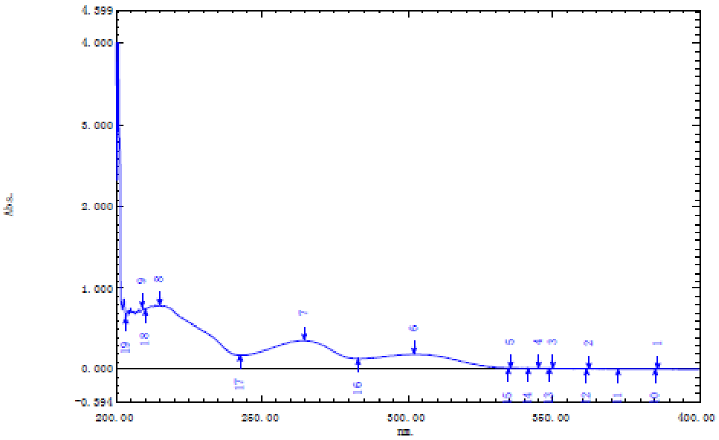

测定属性
波长范围 (nm.): 200.00到400.00
扫描速度: 高速
采样间隔: 0.2
自动采样间隔: 停用
扫描模式: 单一的

试样准备属性
重量: 0.5
体积 : 10
稀释:
光程长: 407
附加信息:

仪器属性
仪器类型: UV-1700
测定方式: 吸收值
狭缝宽: 1.0 nm
光源改变波长: 340.8 nm
S/R 转换: 标准

附件属性
附件: 无

No.	P/V	Wavelength	Abs.	描述
1		386.00	.008	
2		362.40	.013	
3		350.00	.018	
4		345.00	.018	
5		335.40	.024	
6		302.20	.193	
7		264.60	.359	
8		214.80	.792	
9		209.00	.758	
10		385.20	.007	
11		372.40	.008	
12		361.60	.012	
13		348.60	.015	
14		341.60	.016	
15		334.60	.018	
16		283.00	.137	
17		242.60	.181	
18		210.00	.738	
19		203.20	.640	

Figure S5 UV spectrum of penicimenolide A (**1**) in MeOH

Elemental Composition Report

Single Mass Analysis
Tolerance = 5.0 PPM / DBE: min = -1.5, max = 30.0
Element prediction: Off
Number of isotope peaks used for i-FIT = 3

Monoisotopic Mass, Even Electron Ions
276 formula(e) evaluated with 1 results within limits (up to 20 closest results for each mass)
Elements Used:
C: 0-100 H: 0-200 N: 0-2 O: 0-100 Na: 0-1

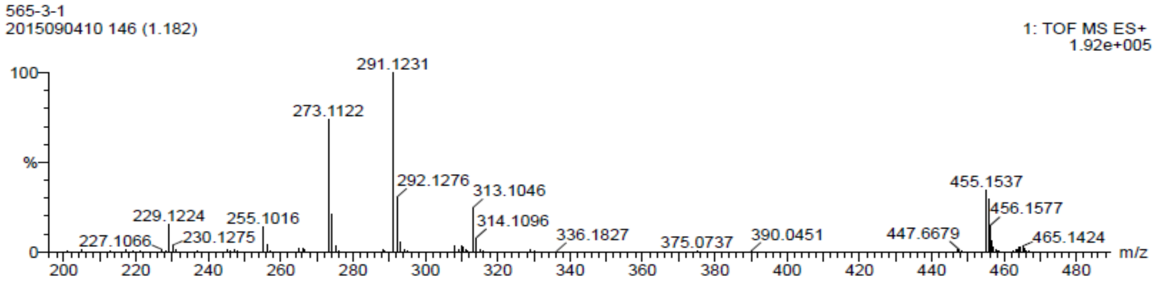

Minimum:				-1.5				
Maximum:		5.0	5.0	30.0				
Mass	Calc. Mass	mDa	PPM	DBE	i-FIT	Norm	Conf(%)	Formula
291.1231	291.1232	-0.1	-0.3	7.5	339.2	n/a	n/a	C16 H19 O5

Figure S6 HR-ESI-MS spectrum of penicimenolide A (**1**)

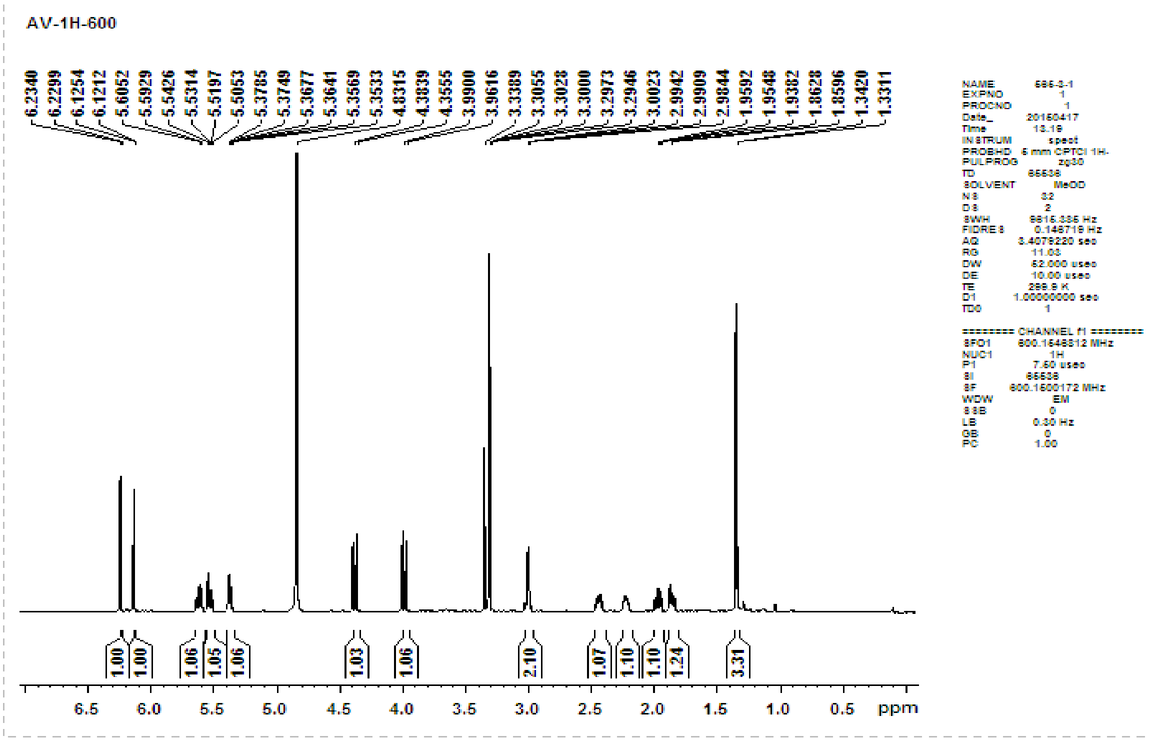

Figure S7 ^{1}H-NMR spectrum of **1** (600MHz, in CD_3OD)

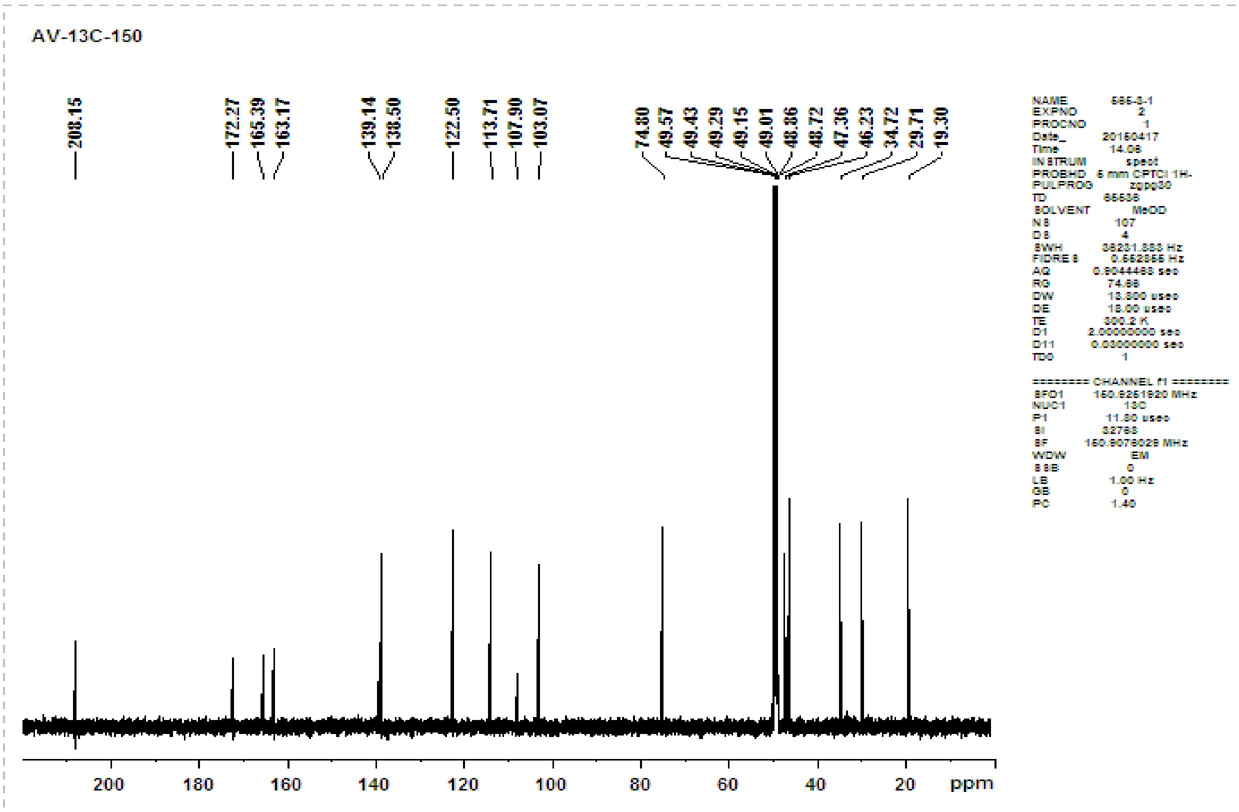

Figure S8 ^{13}C-NMR spectrum of **1** (150MHz, in CD_3OD)

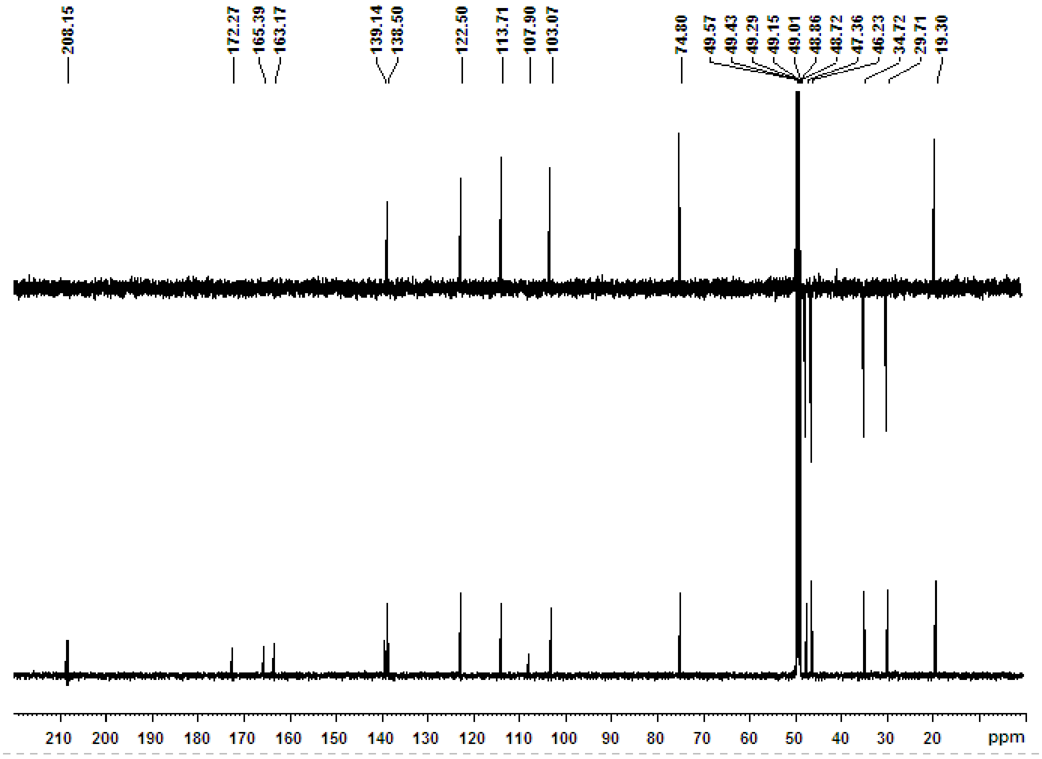

Figure S9 ^{13}C-NMR and DEPT 135 spectra of **1** (150MHz, in CD_3OD)

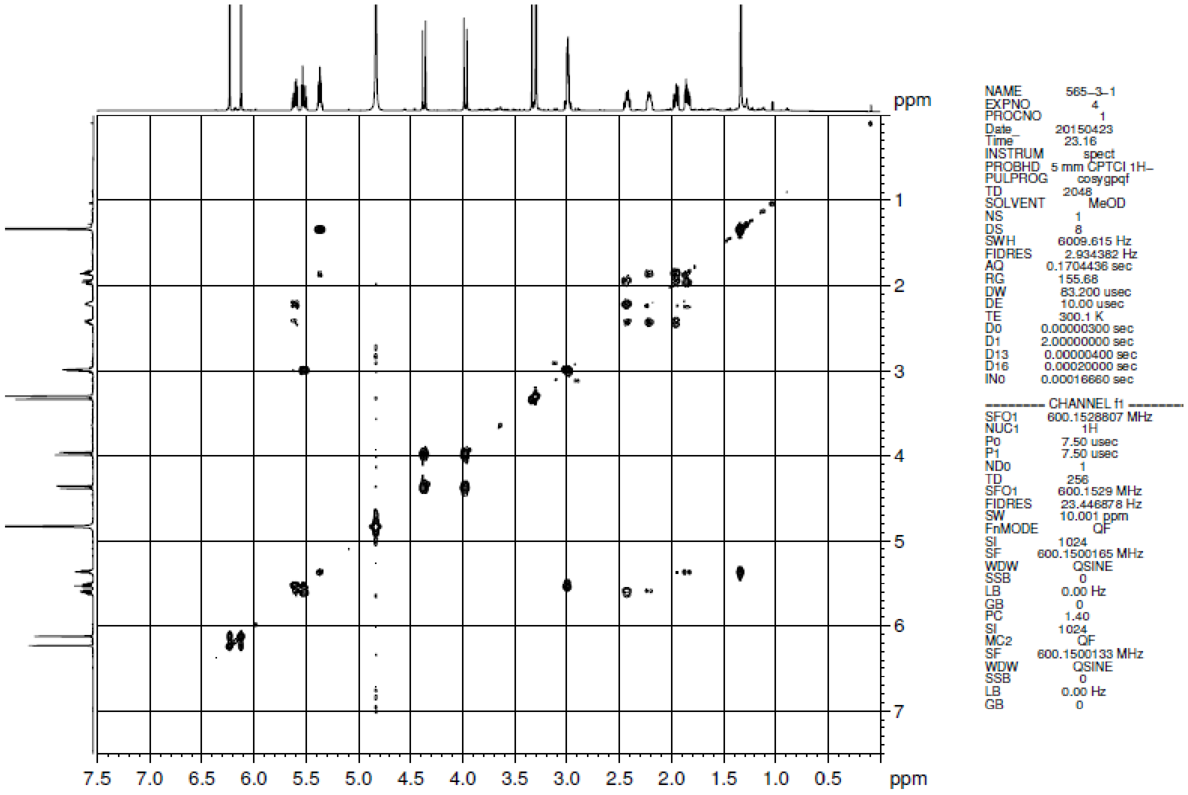

Figure S10 ^{1}H-^{1}H COSY spectrum of **1** (600MHz, in CD_3OD)

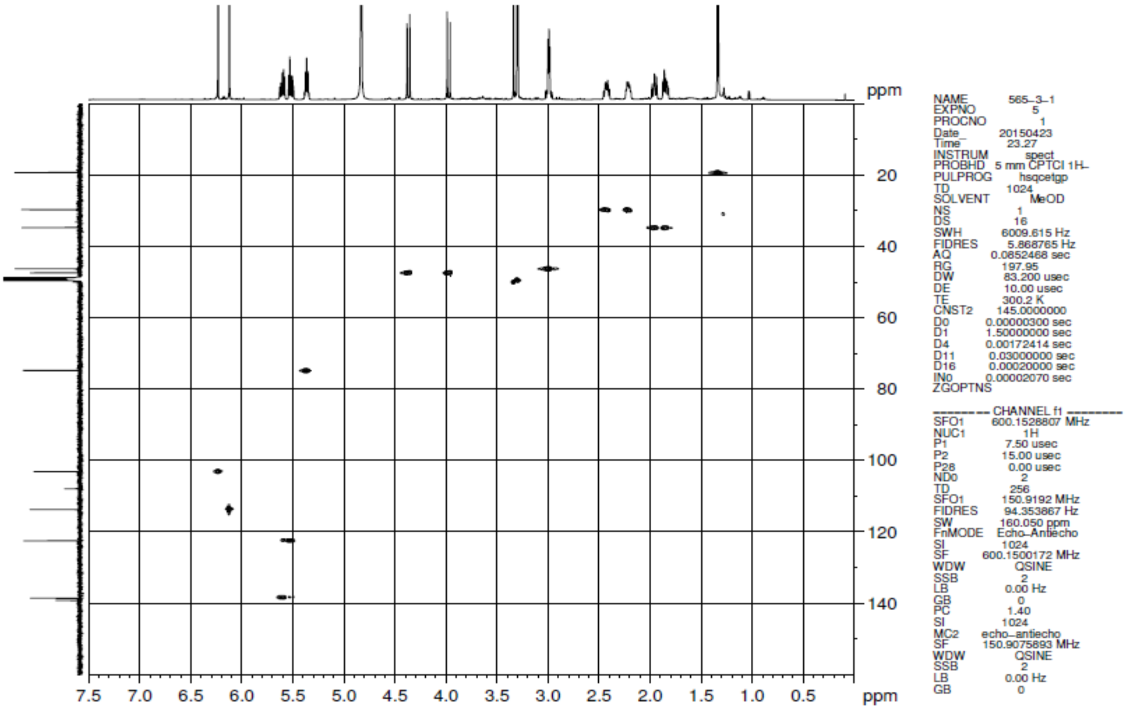

Figure S11 HSQC spectrum of **1** (600MHz, in CD_3OD)

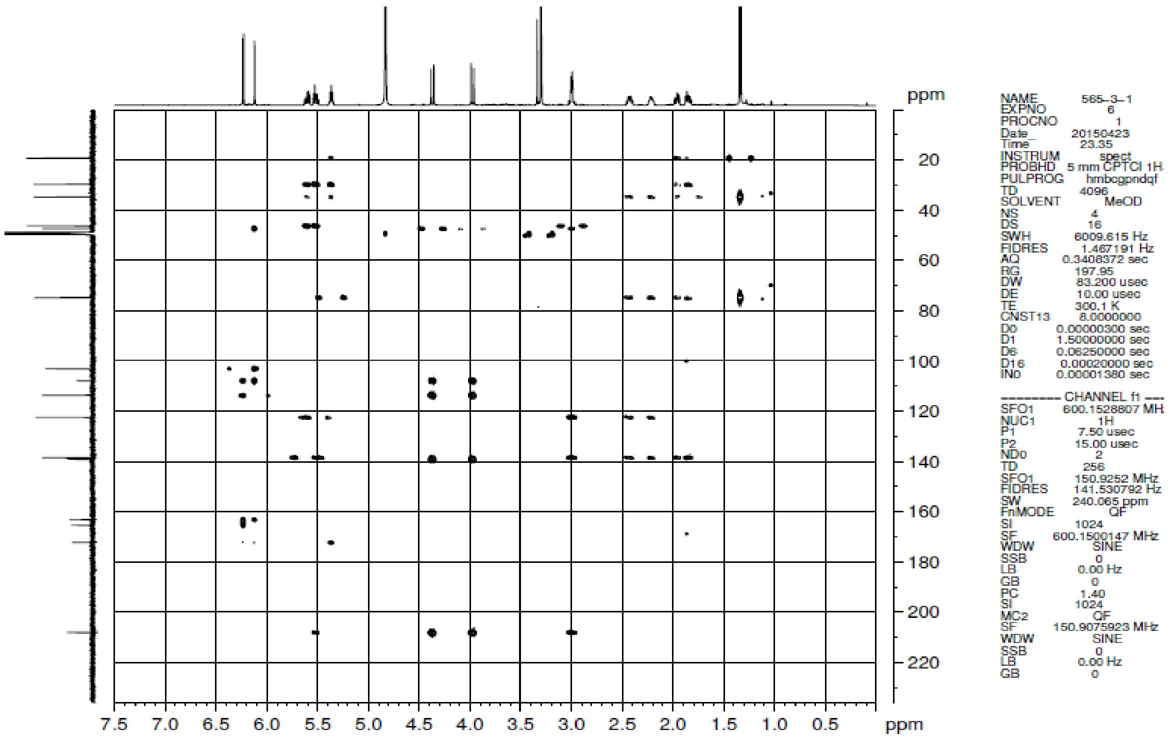

Figure S12 HMBC spectrum of **1** (600MHz, in CD_3OD)

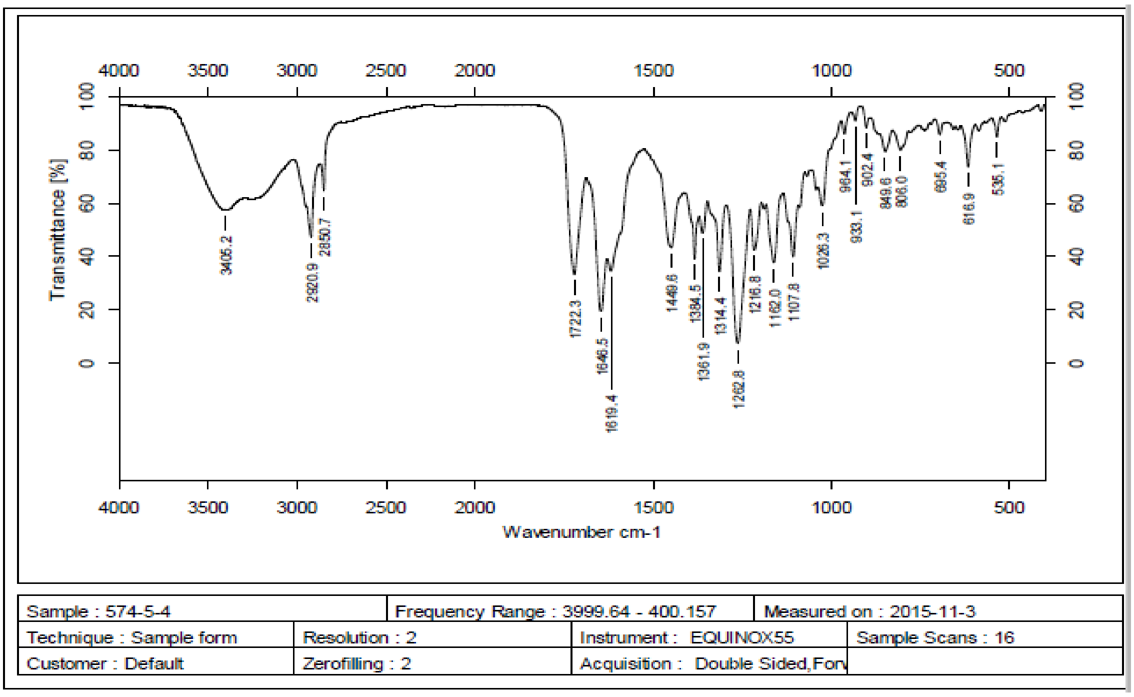

Figure S13 IR (KBr disc) spectrum of penicimenolide B (**2**)

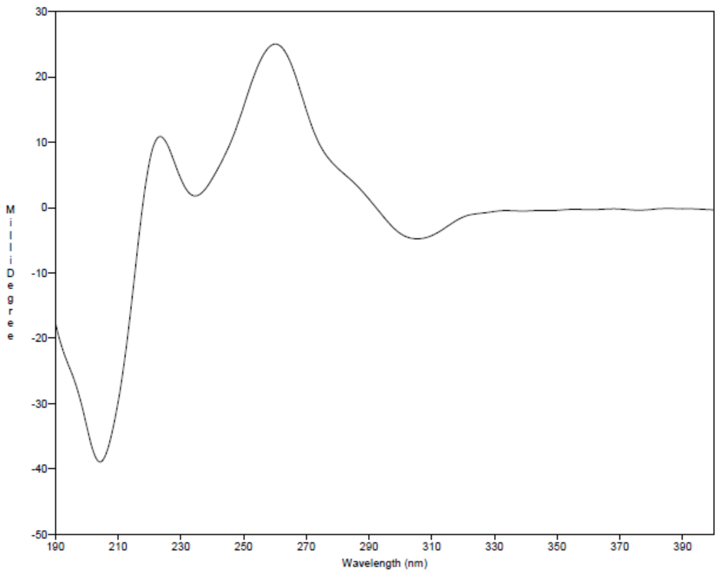

Figure S14 CD spectrum of penicimenolide B (**2**) in MeOH

Spectrum Peak Pick Report

FIELD FIELD TEXT

Data Set: 没有

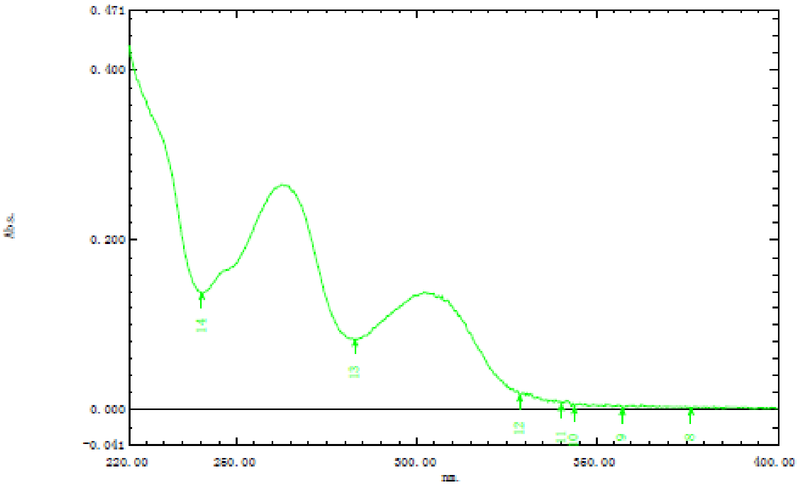

测定属性
波长范围 (nm.): 220. 00到400. 00
扫描速度: 高速
采样间隔: 0. 2
自动采样间隔: 启用
扫描模式: 单一的

试样准备属性
重量: 0. 5
体积 : 10
稀释:
光程长: 407
附加信息:

仪器属性
仪器类型: UV-1700
测定方式: 吸收值
狭缝宽: 1. 0 nm
光源改变波长: 340. 8 nm
S/R 转换: 标准

No.	P/V	Wavelength	Abs.	描述
1	↑	382.80	.004	
2	↑	362.80	.006	
3	↑	352.60	.008	
4	↑	341.80	.011	
5	↑	329.40	.020	
6	↑	302.00	.139	
7	↑	262.40	.266	
8	↓	376.20	.003	
9	↓	357.20	.003	
10	↓	343.80	.004	
11	↓	340.20	.007	
12	↓	326.60	.018	
13	↓	282.60	.083	
14	↓	240.20	.137	

Figure S15 UV spectrum of penicimenolide B (**2**) in MeOH

Elemental Composition Report **Page 1**

Single Mass Analysis
Tolerance = 5.0 mDa / DBE: min = -1.5, max = 50.0
Element prediction: Off
Number of isotope peaks used for i-FIT = 3

Monoisotopic Mass, Even Electron Ions
72 formula(e) evaluated with 2 results within limits (up to 10 best isotopic matches for each mass)
Elements Used:
C: 0-500 H: 0-1000 O: 0-200
574-5-4
2015033009 152 (1.238) 1: TOF MS ES+ 3.00e+005

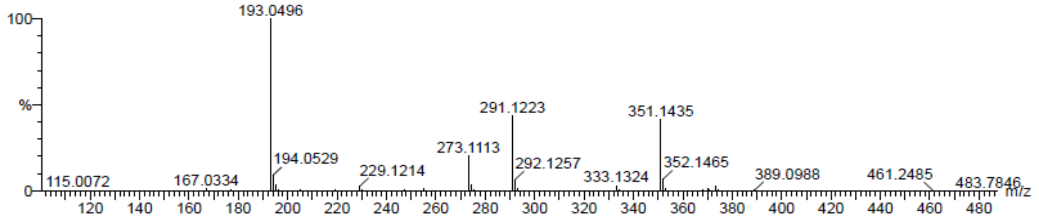

Minimum:				-1.5				
Maximum:		5.0	10.0	50.0				
Mass	Calc. Mass	mDa	PPM	DBE	i-FIT	Norm	Conf(%)	Formula
351.1435	351.1444	-0.9	-2.6	7.5	173.2	0.006	99.44	C18 H23 O7
	351.1385	5.0	14.2	16.5	178.4	5.187	0.56	C25 H19 O2

Figure S16 HR-ESI-MS spectrum of penicimenolide B (**2**)

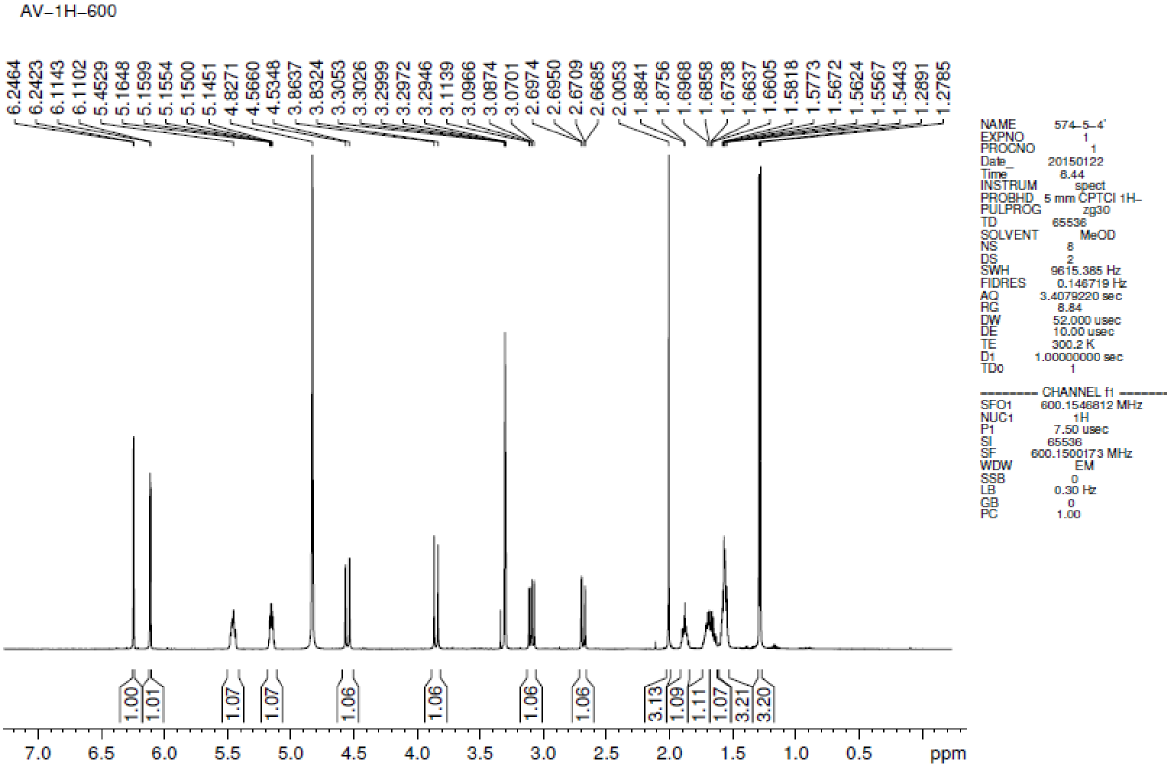

Figure S17 ^{1}H-NMR spectrum of **2** (600 MHz, in CD_3OD)

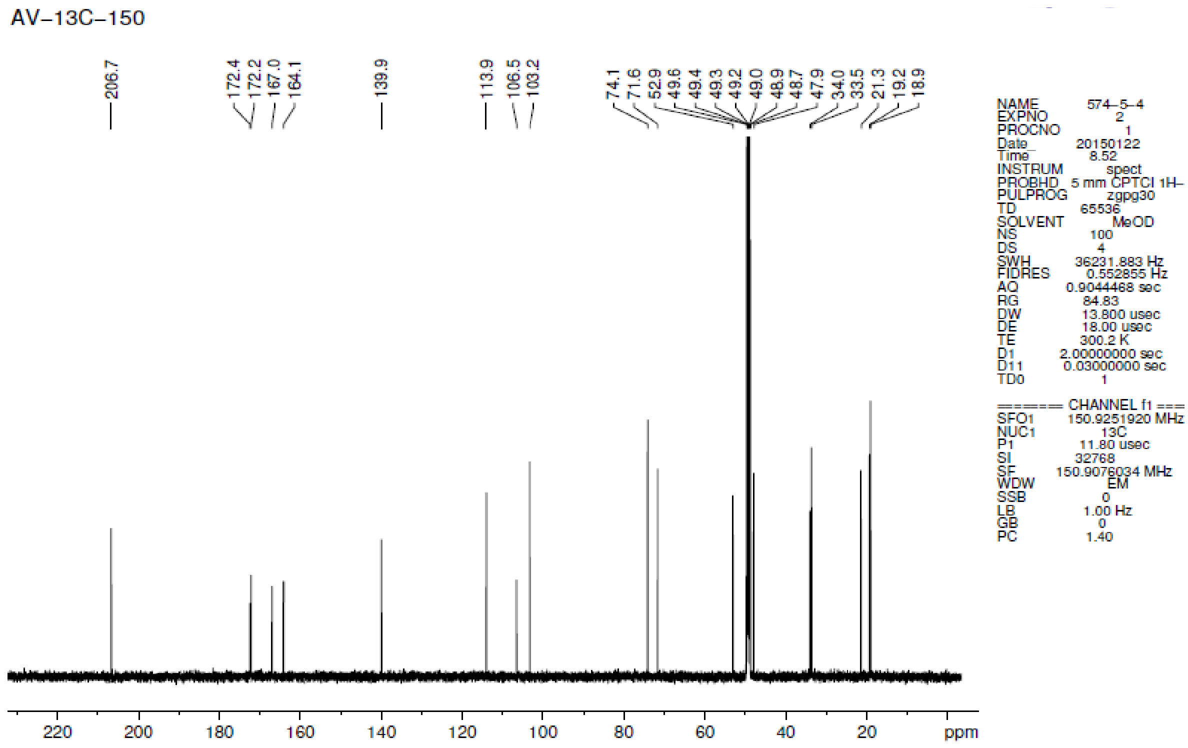

Figure S18 ^{13}C-NMR spectrum of **2** (150MHz, in CD_3OD)

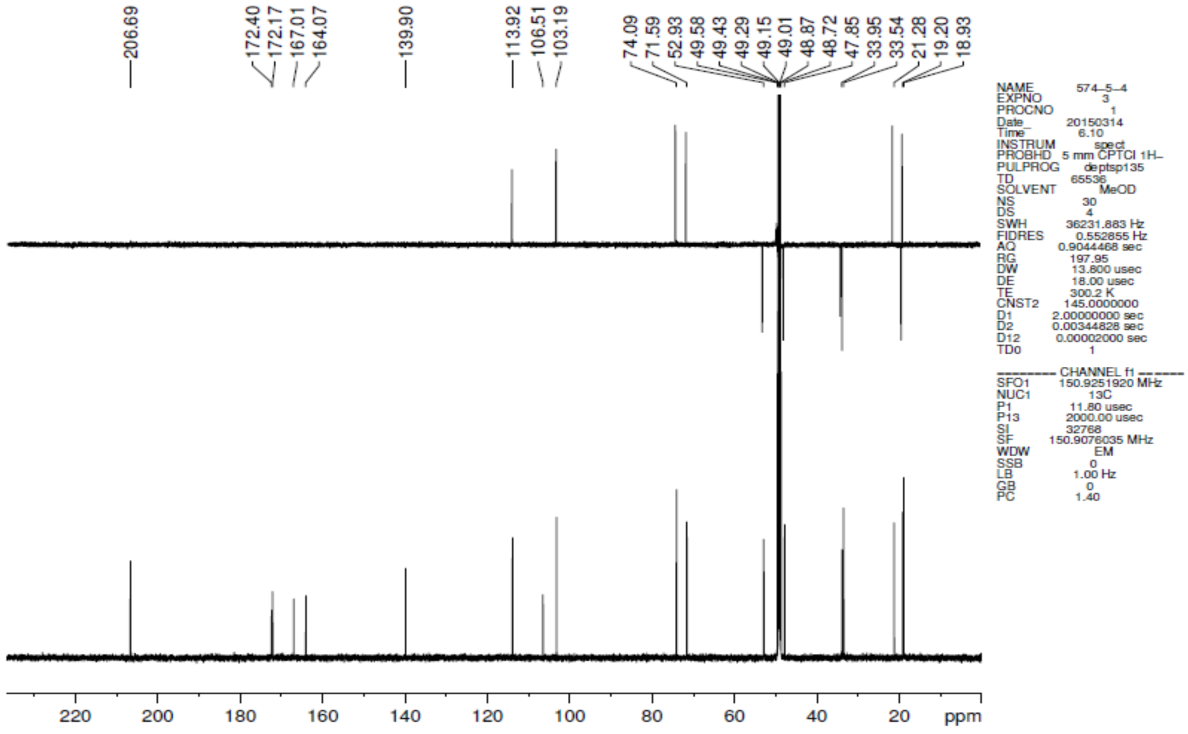

Figure S19 ^{13}C-NMR and DEPT 135 spectra of **2** (150MHz, in CD_3OD)

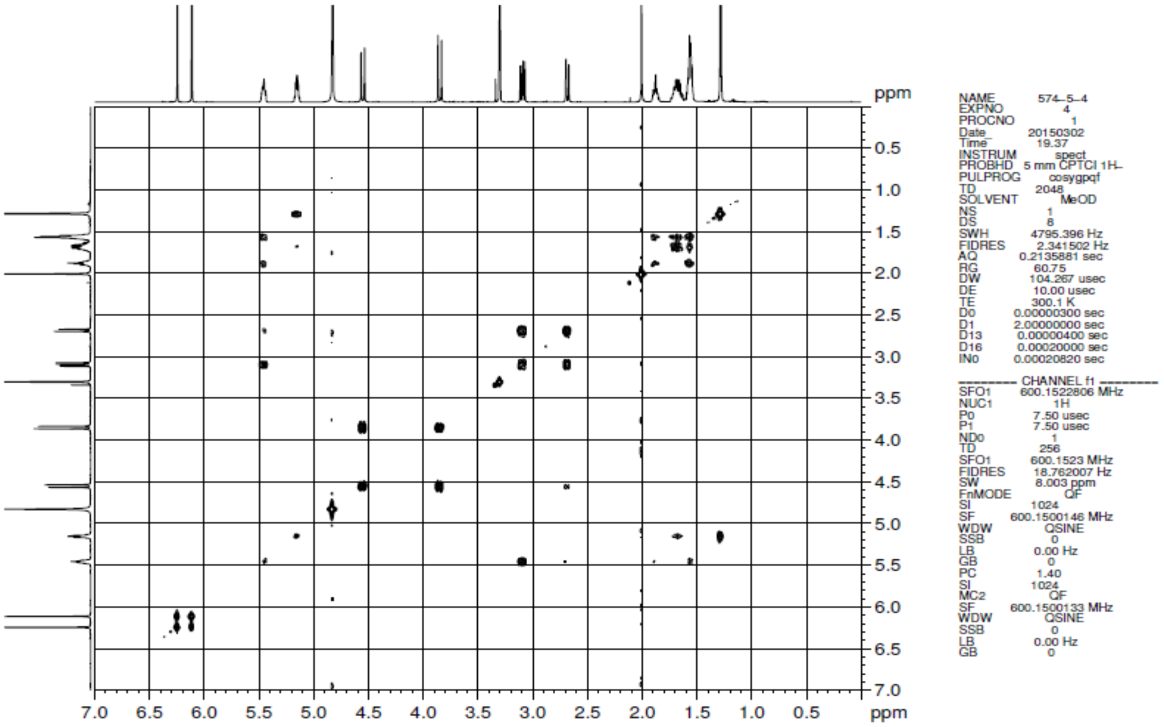

Figure S20 ^{1}H-^{1}H COSY spectrum of **2** (600MHz, in CD_3OD)

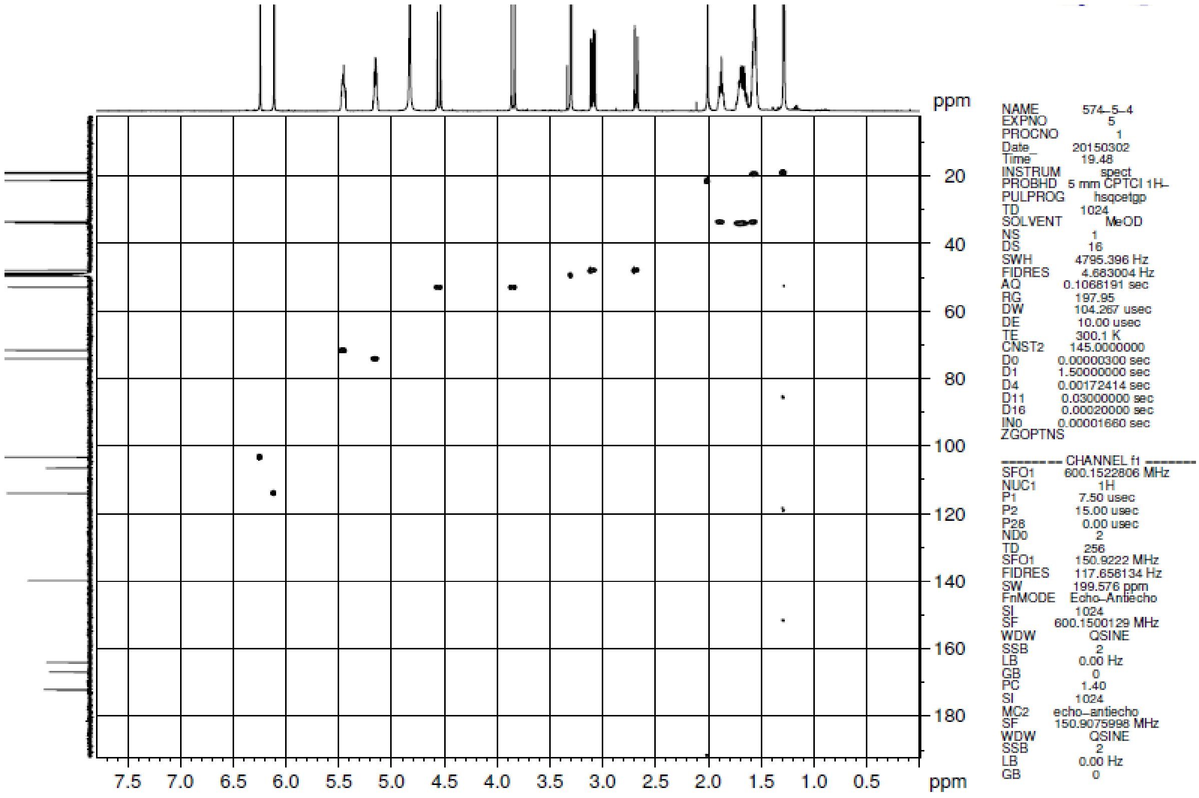

Figure S21 HSQC spectrum of **2** (600MHz, in CD_3OD)

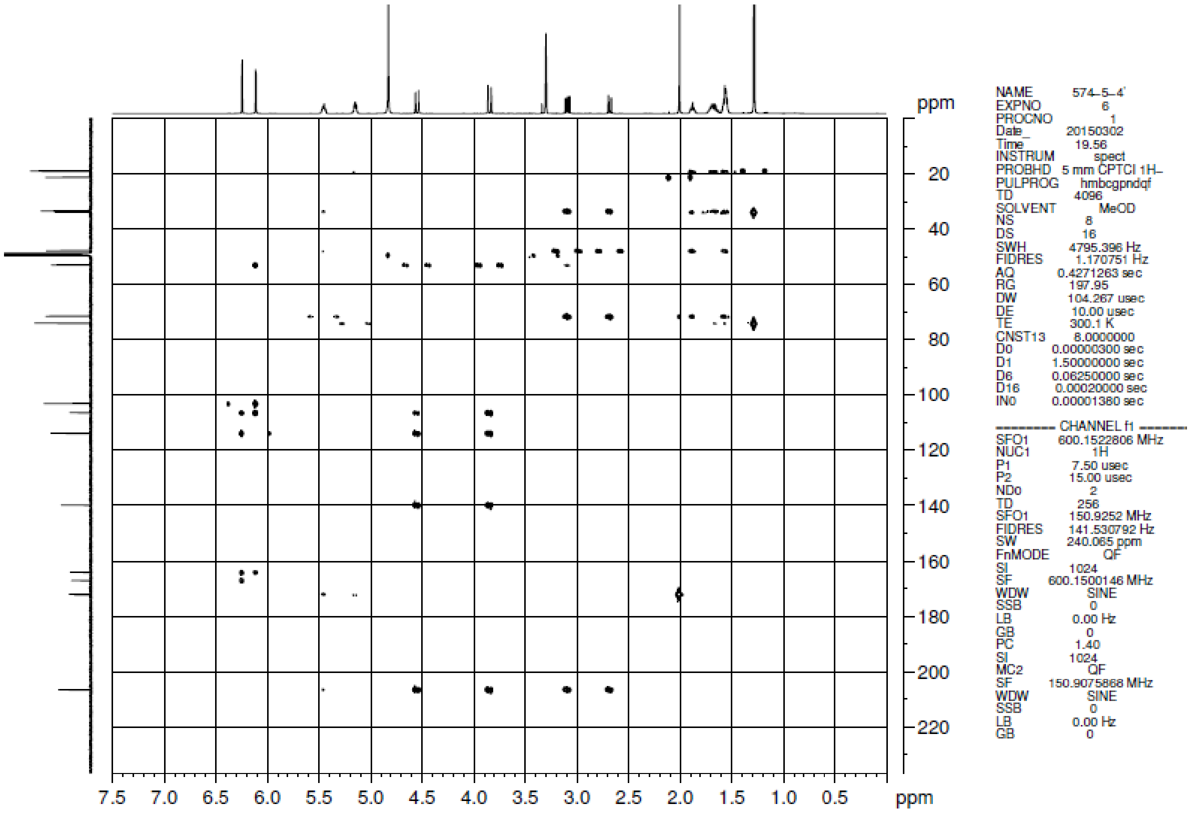

Figure S22 HMBC spectrum of **2** (600MHz, in CD)

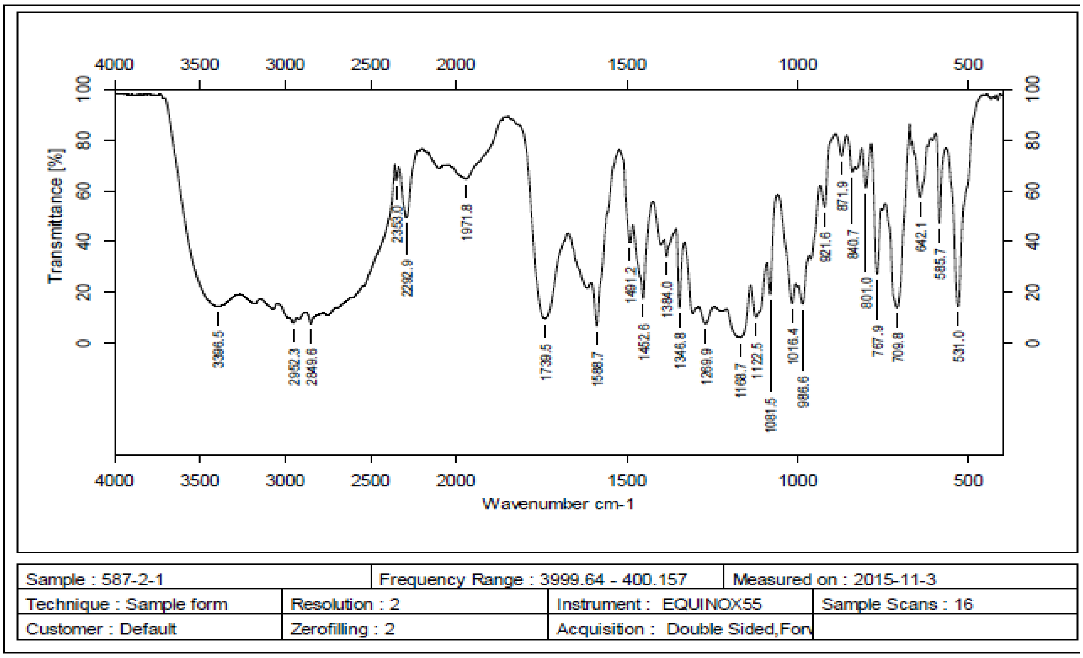

Figure S23 IR (KBr disc) spectrum of penicimenolide C (**3**)

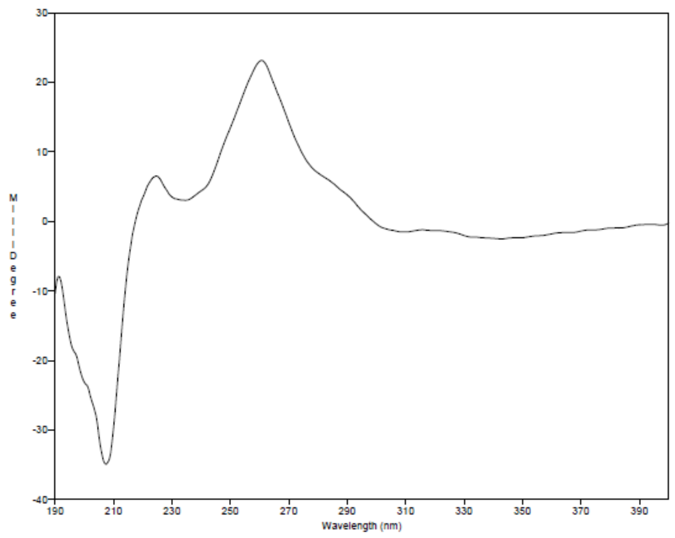

Figure S24 CD spectrum of penicimenolide C (**3**) in MeOH

Spectrum Peak Pick Report

FIELD FIELD TEXT

Data Set: 没有

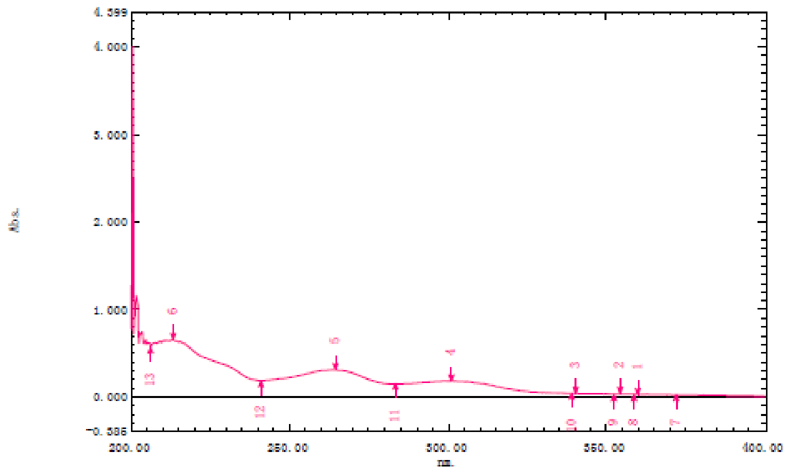

测定属性
波长范围 (nm.): 200.00到400.00
扫描速度: 高速
采样间隔: 0.2
自动采样间隔: 停用
扫描模式: 单一的

试样准备属性
重量: 0.5
体积 : 10
稀释:
光程长: 407
附加信息:

仪器属性
仪器类型: UV-1700
测定方式: 吸收值
狭缝宽: 1.0 nm
光源改变波长: 340.8 nm
S/R 转换: 标准

附件属性
附件: 无

No.	P/V	Wavelength	Abs.	描述
1	T	359.80	.035	
2	T	354.40	.037	
3	T	340.40	.045	
4	T	301.00	.166	
5	T	264.60	.314	
6	T	213.20	.658	
7	↓	372.00	.026	
8	↓	358.80	.033	
9	↓	352.20	.034	
10	↓	339.20	.042	
11	↓	283.20	.152	
12	↓	241.00	.189	
13	↓	206.20	.577	

Figure S25 UV spectrum of penicimenolide C (**3**) in MeOH

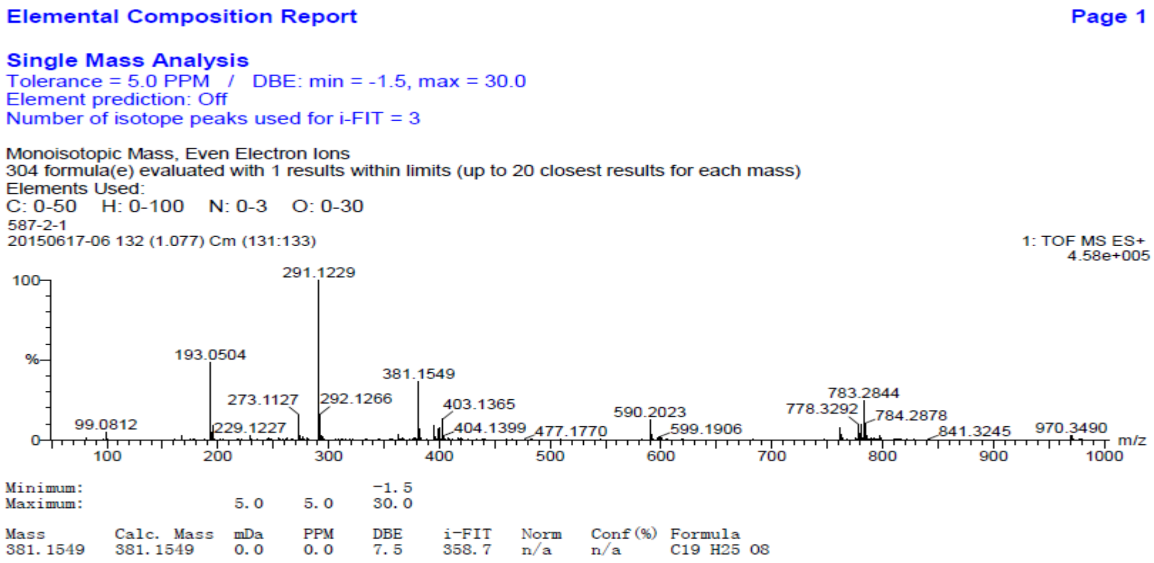

Figure S26 HR-ESI-MS spectrum of penicimenolide C (**3**)

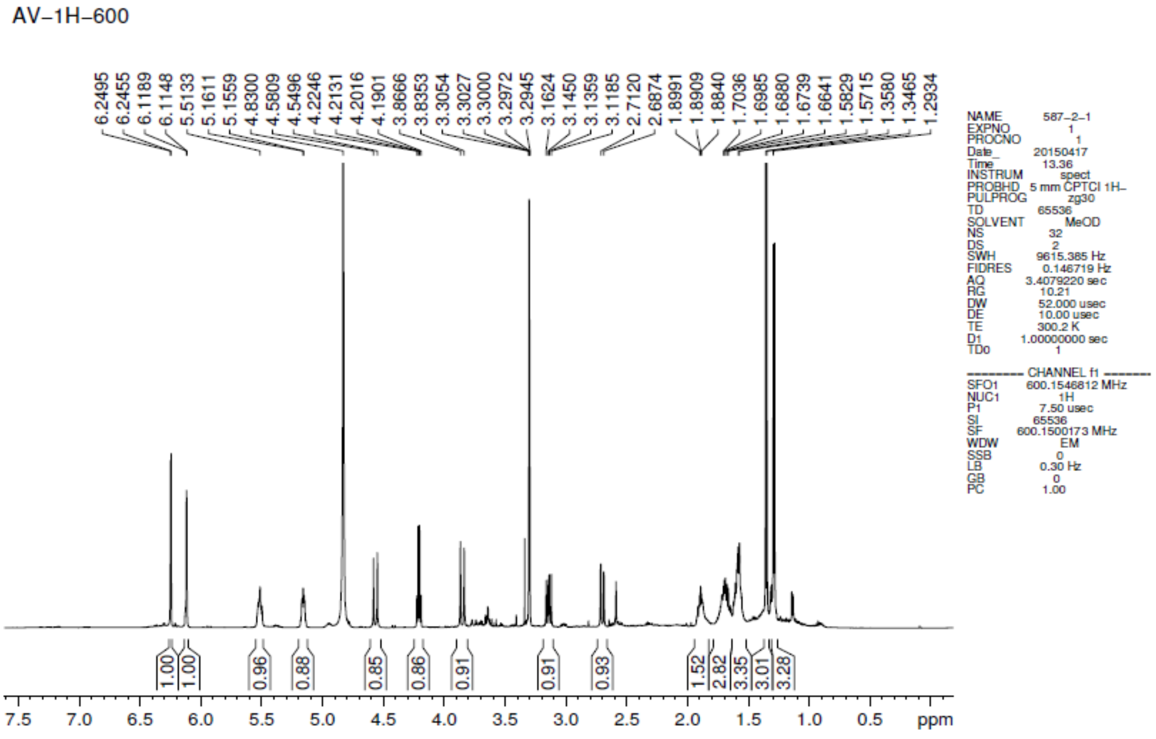

Figure S27 ^{1}H-NMR spectrum of **3** (600MHz, in CD_3OD)

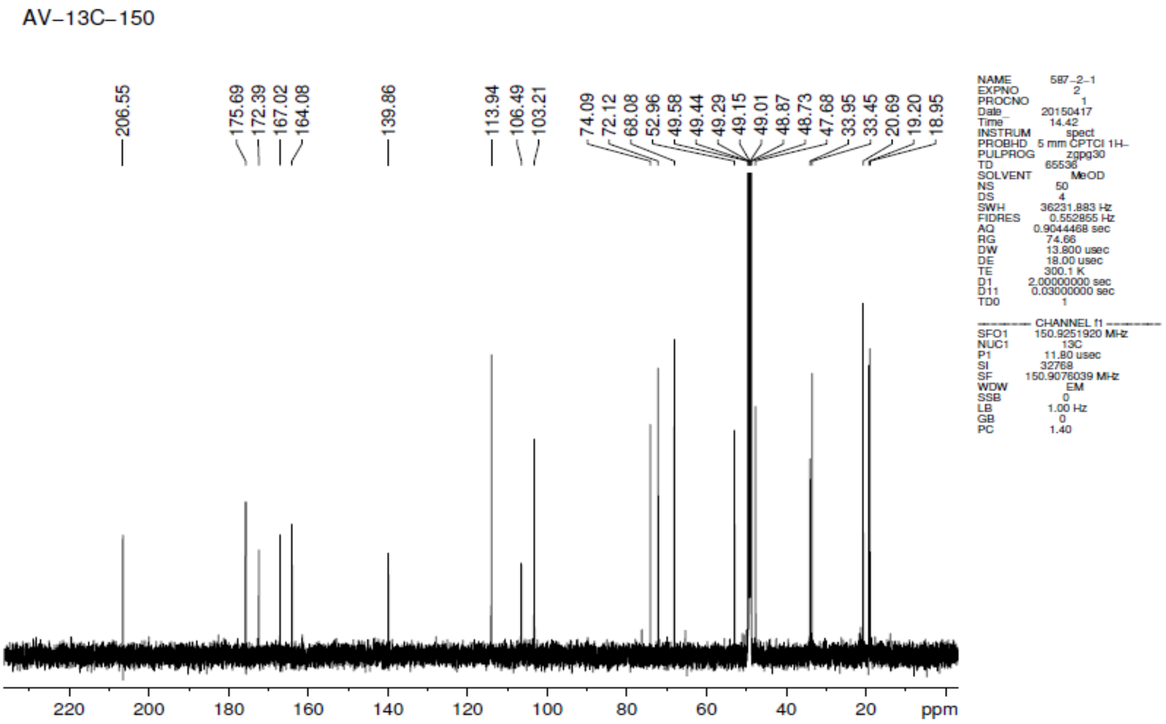

Figure S28 ^{13}C-NMR spectrum of **3** (150MHz, in CD_3OD)

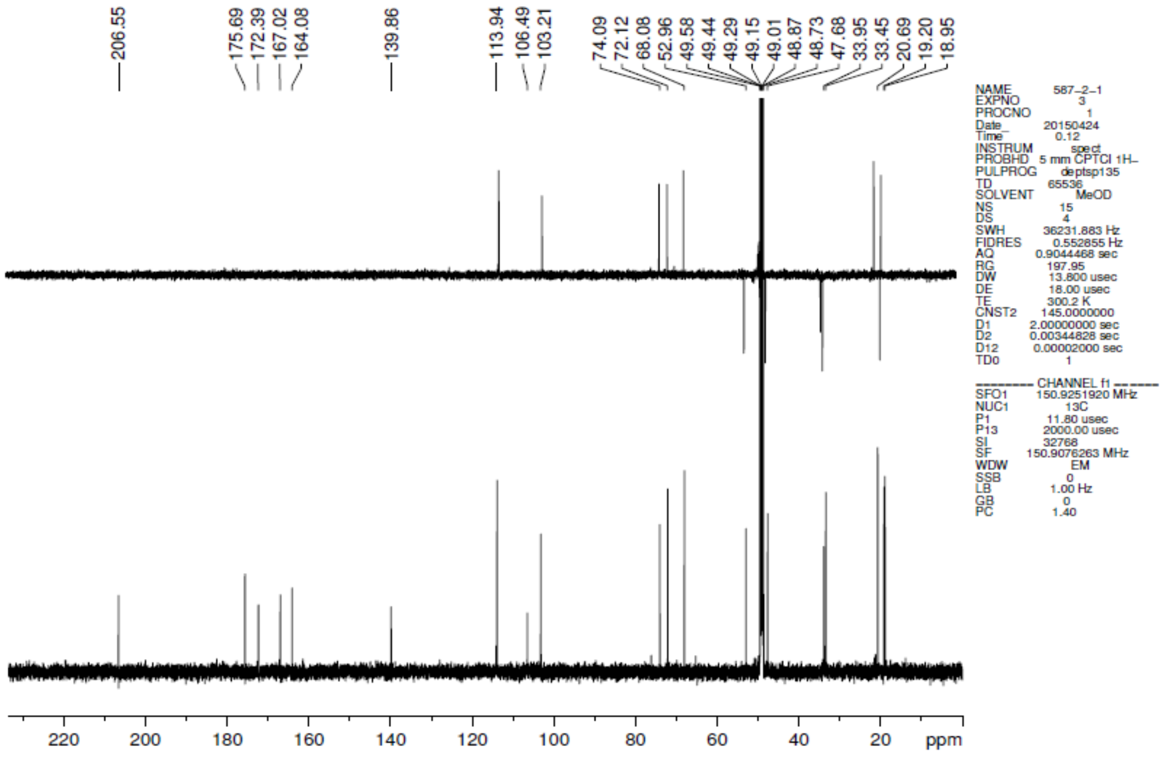

Figure S29 ^{13}C-NMR and DEPT 135 spectra of **3** (150MHz, in CD_3OD)

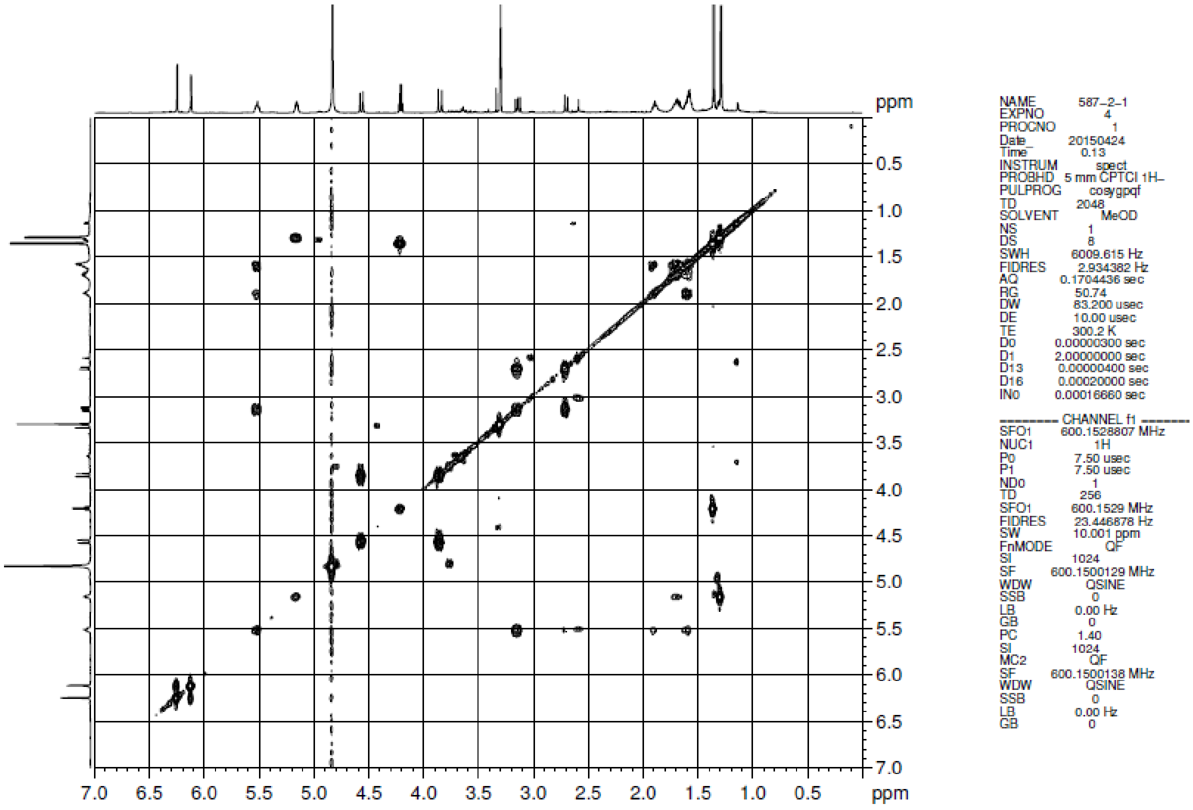

Figure S30 ^{1}H-^{1}H COSY spectrum of **3** (600MHz, in CD_3OD)

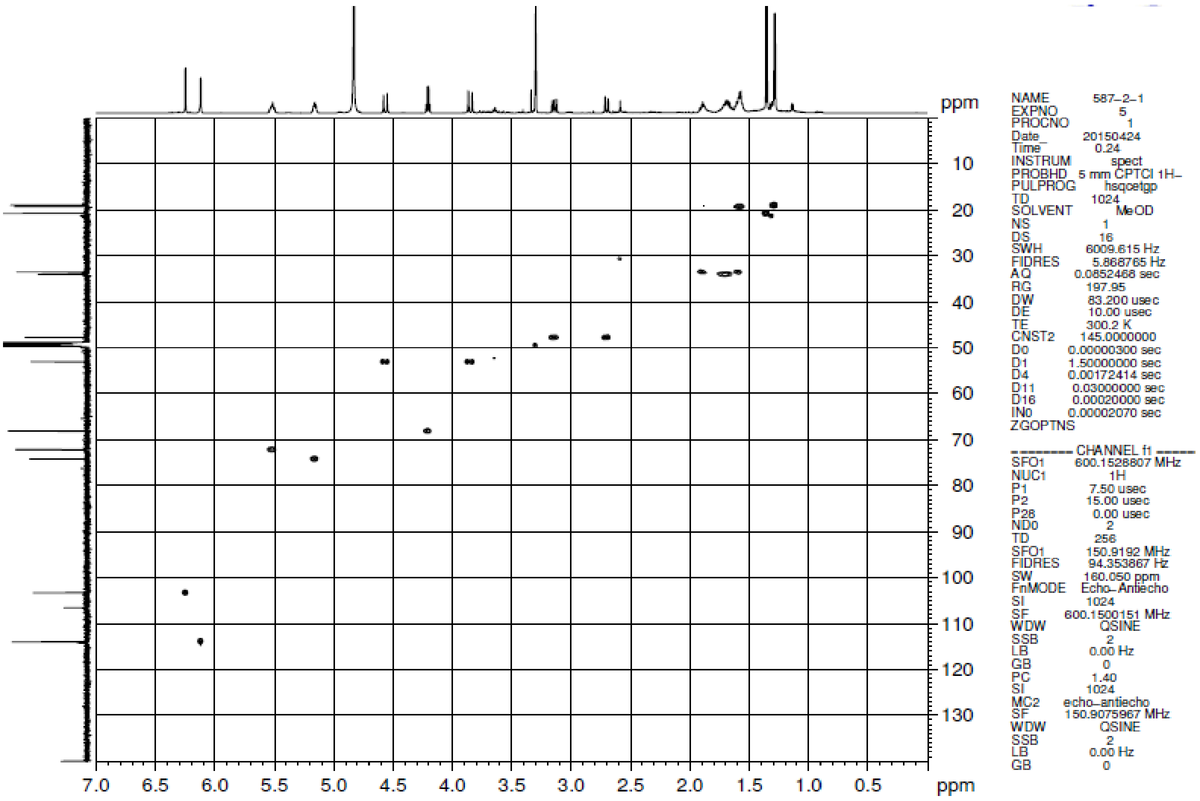

Figure S31 HSQC spectrum of **3** (600MHz, in CD_3OD)

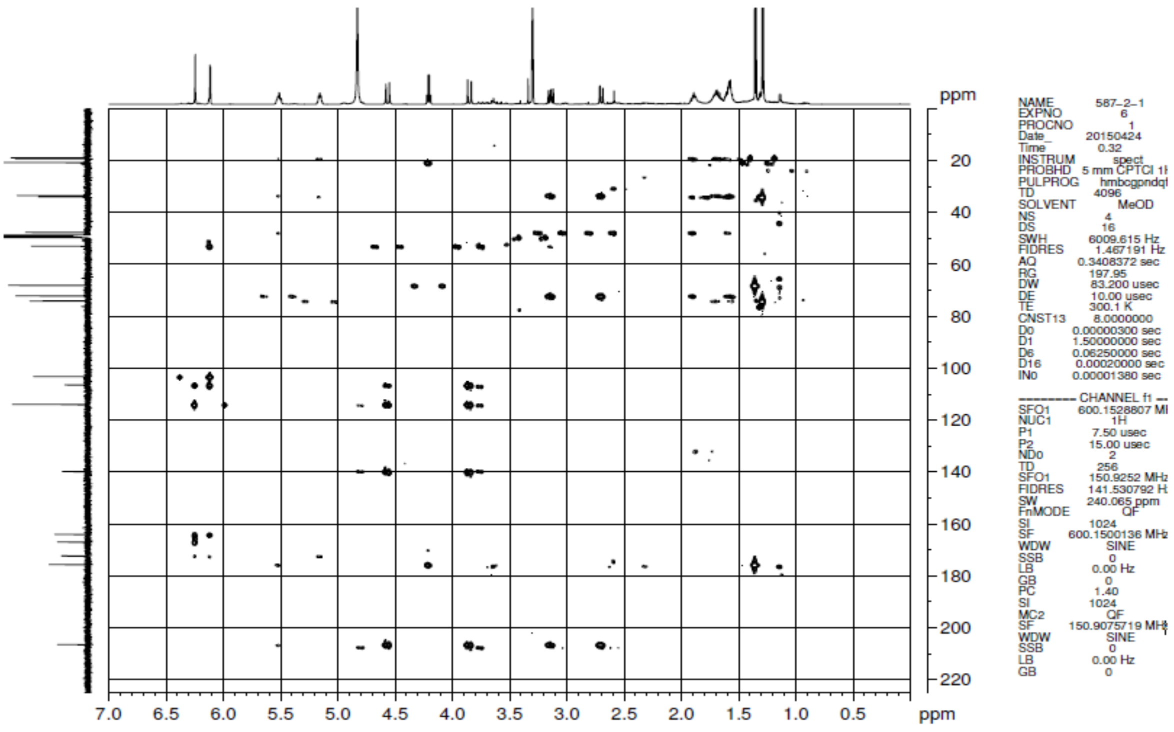

Figure S32 HMBC spectrum of **3** (600MHz, in CD_3OD)

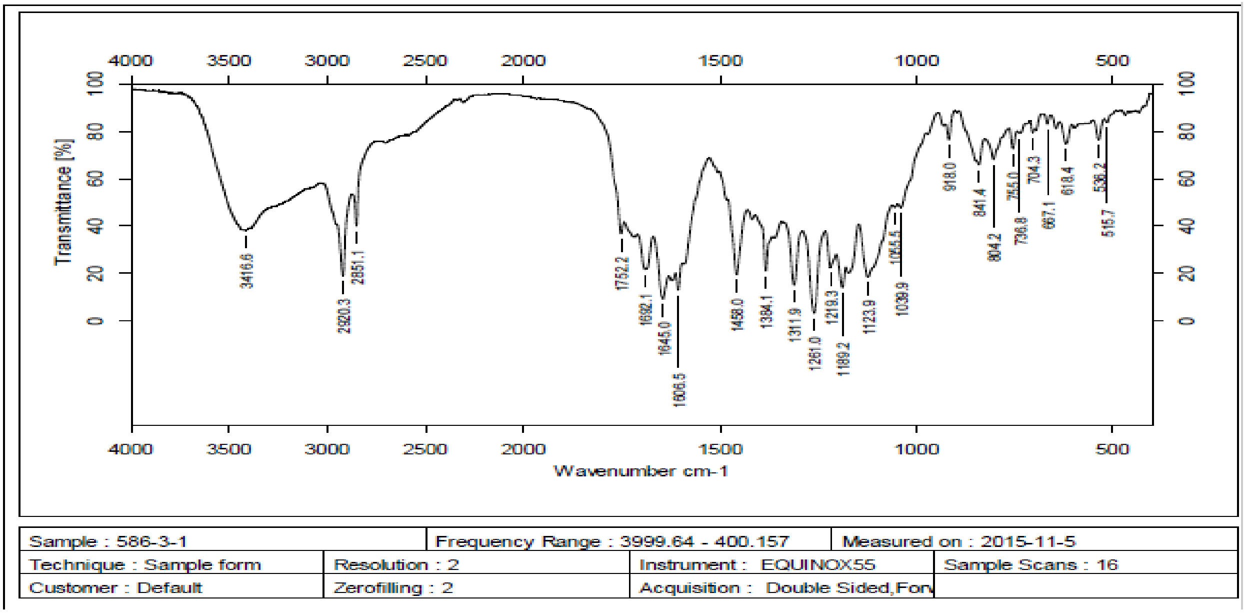

Figure S33 IR (KBr disc) spectrum of penicimenolide D (**4**)

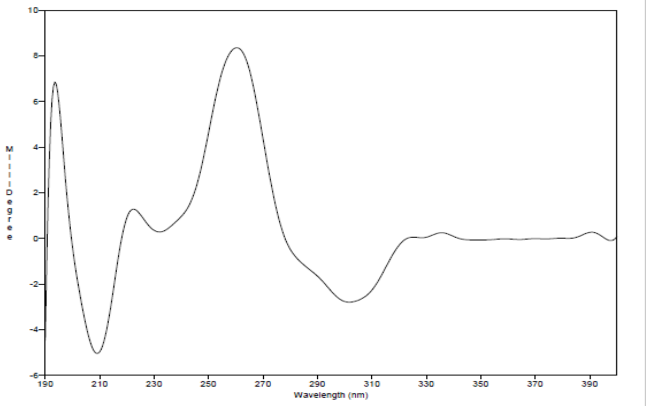

Figure S34 CD spectrum of penicimenolide D (**4**) in MeOH

Spectrum Peak Pick Report FIELD FIELD TEXT

Data Set: 没有

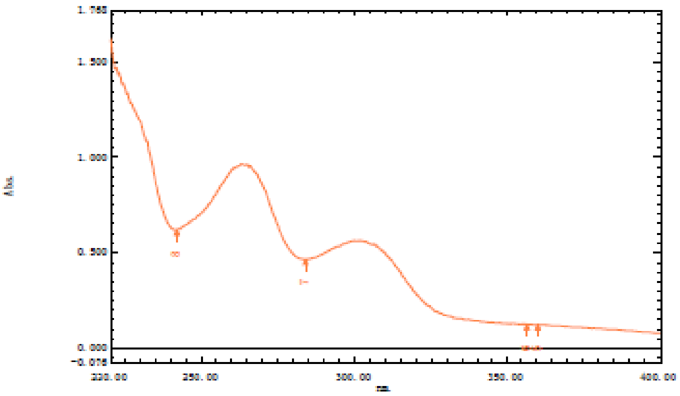

测定属性	
波长范围（nm.）:	220.00到400.00
扫描速度:	高速
采样间隔:	0.2
自动采样间隔:	启用
扫描模式:	单一的
试样准备属性	
重量:	0.3
体积 :	10
稀释:	
光程长:	407
附加信息:	
仪器属性	
仪器类型:	UV-1700
测定方式:	吸收值
狭缝宽:	1.0 nm
光源改变波长:	340.8 nm
S/R 转换:	标准
附件属性	
附件:	无

No.	P/V	Wavelength	Abs.	描述
1	⬆	361.40	.124	
2	⬆	357.80	.126	
3	⬆	301.80	.563	
4	⬆	264.80	.961	
5	⬇	360.00	.120	
6	⬇	356.20	.124	
7	⬇	283.80	.461	
8	⬇	241.60	.617	

FIELD TEXT

Figure S35 UV spectrum of penicimenolide D (**4**) in MeOH

Single Mass Analysis
Tolerance = 5.0 PPM / DBE: min = -1.5, max = 30.0
Element prediction: Off
Number of isotope peaks used for i-FIT = 3

Monoisotopic Mass, Even Electron Ions
304 formula(e) evaluated with 1 results within limits (up to 20 closest results for each mass)
Elements Used:
C: 0-50 H: 0-100 N: 0-3 O: 0-30
586-3-1
20150617-08 121 (0.984) Cm (120:124)

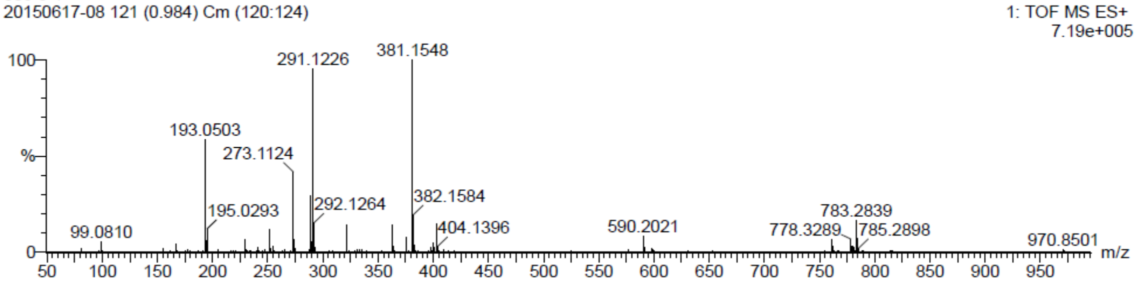

Minimum:				-1.5				
Maximum:		5.0	5.0	30.0				
Mass	Calc. Mass	mDa	PPM	DBE	i-FIT	Norm	Conf(%)	Formula
381.1548	381.1549	-0.1	-0.3	7.5	619.6	n/a	n/a	C19 H25 O8

Figure S36 HR-ESI-MS spectrum of penicimenolide D (**4**)

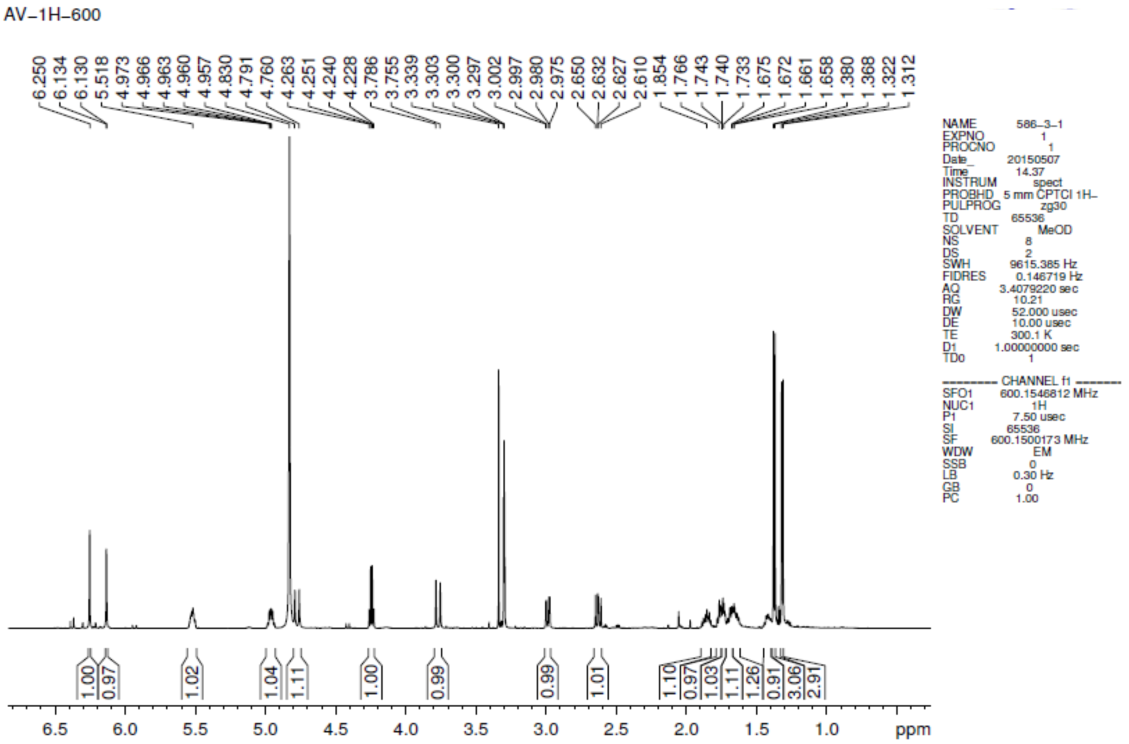

Figure S37 ^{1}H-NMR spectrum of **4** (600MHz, in CD_3OD)

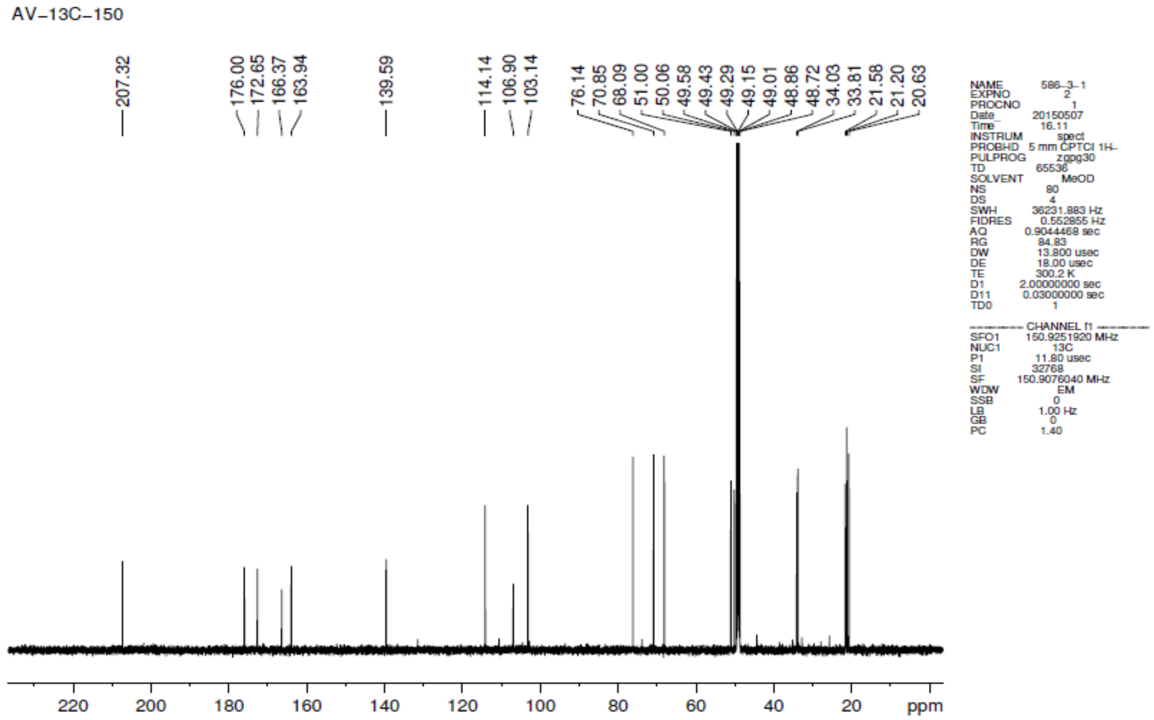

Figure S38 ^{13}C-NMR spectrum of **4** (150MHz, in CD_3OD)

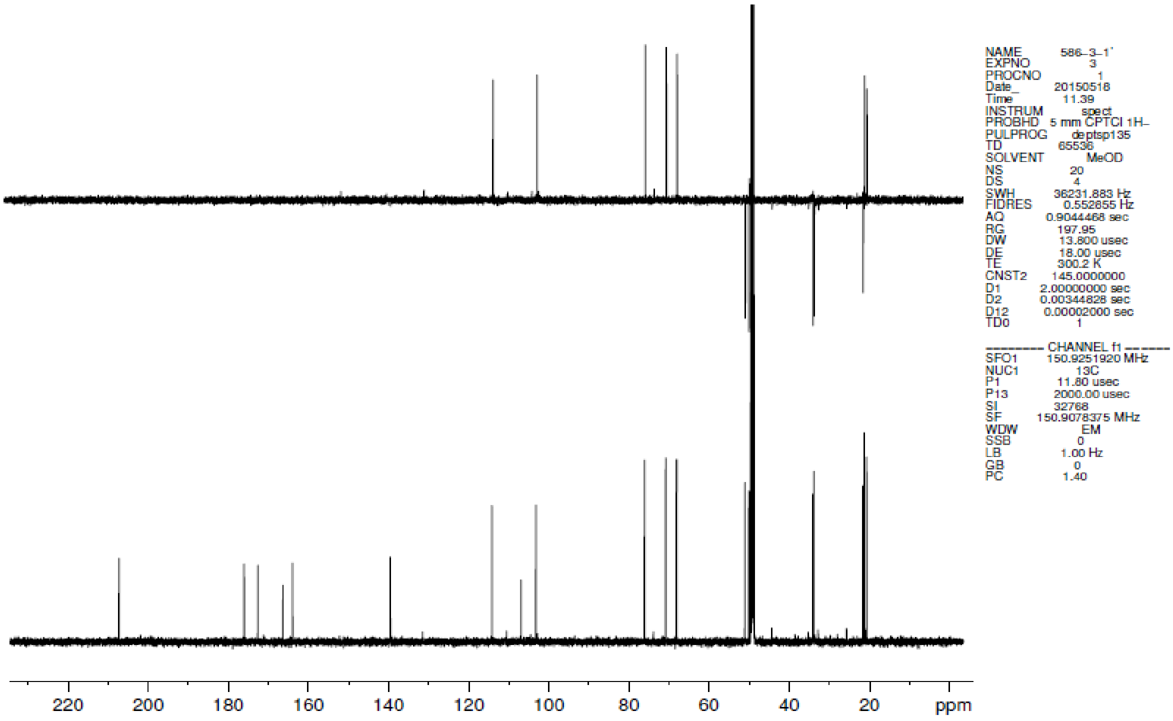

Figure S39 ^{13}C-NMR and DEPT 135 spectra of **4** (150MHz, in CD_3OD)

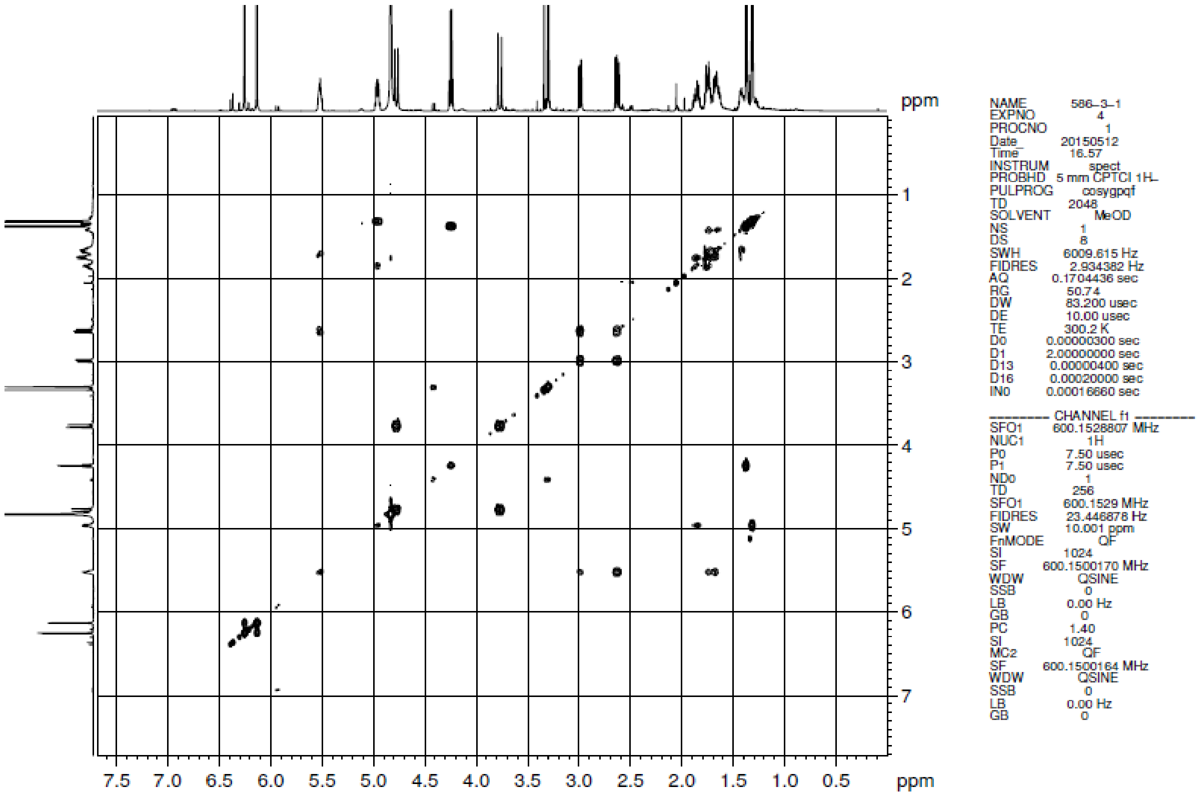

Figure S40 ^{1}H-^{1}H COSY spectrum of **4** (600MHz, in CD_3OD)

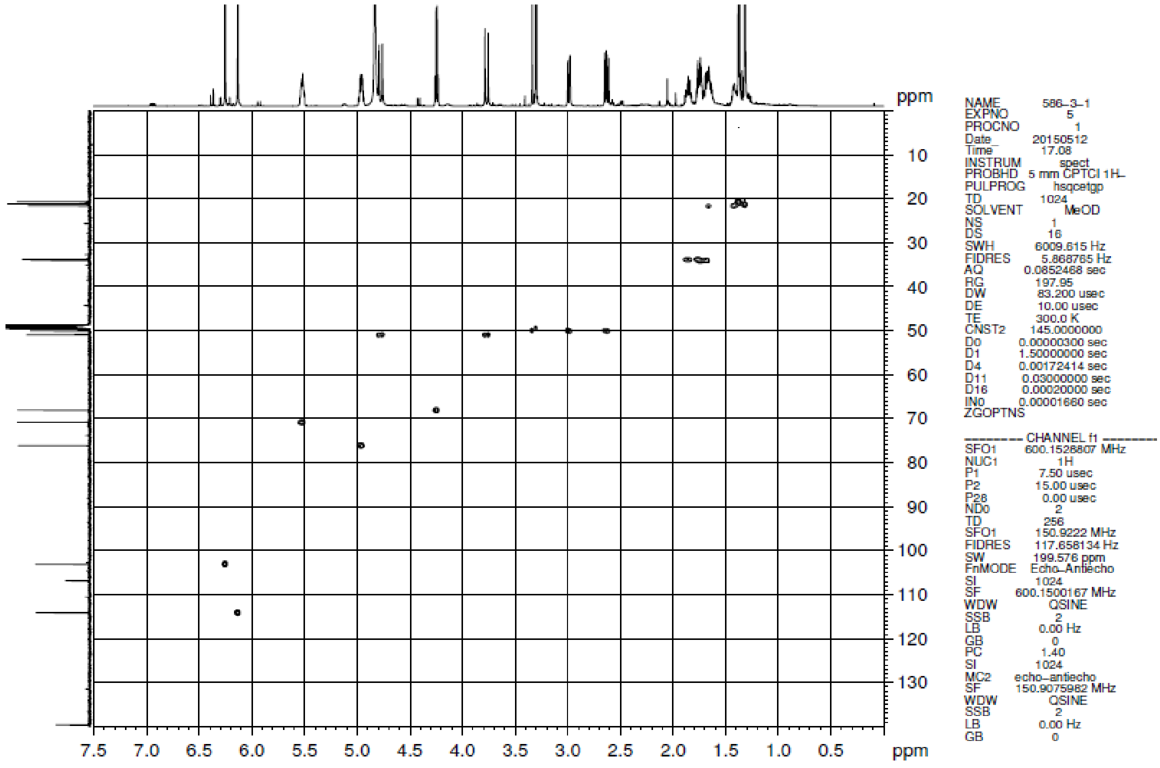

Figure S41 HSQC spectrum of **4** (600MHz, in CD_3OD)

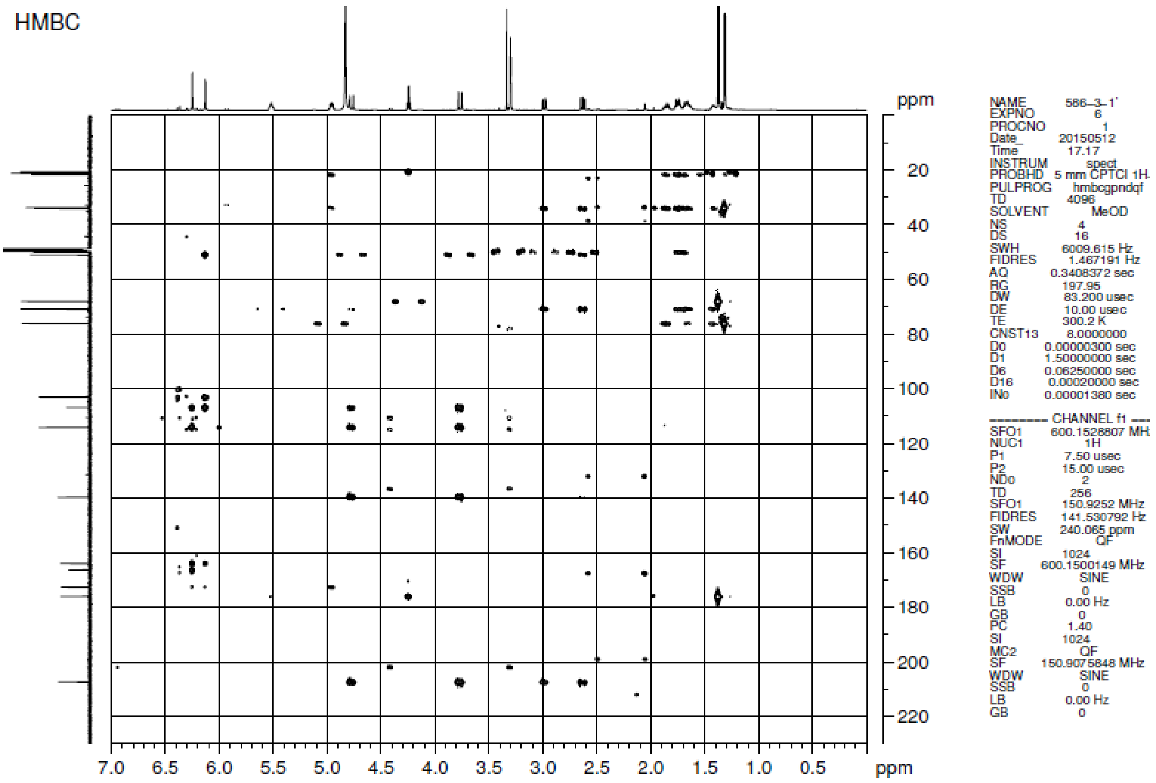

Figure S42 HMBC spectrum of **4** (600MHz, in CD_3OD)

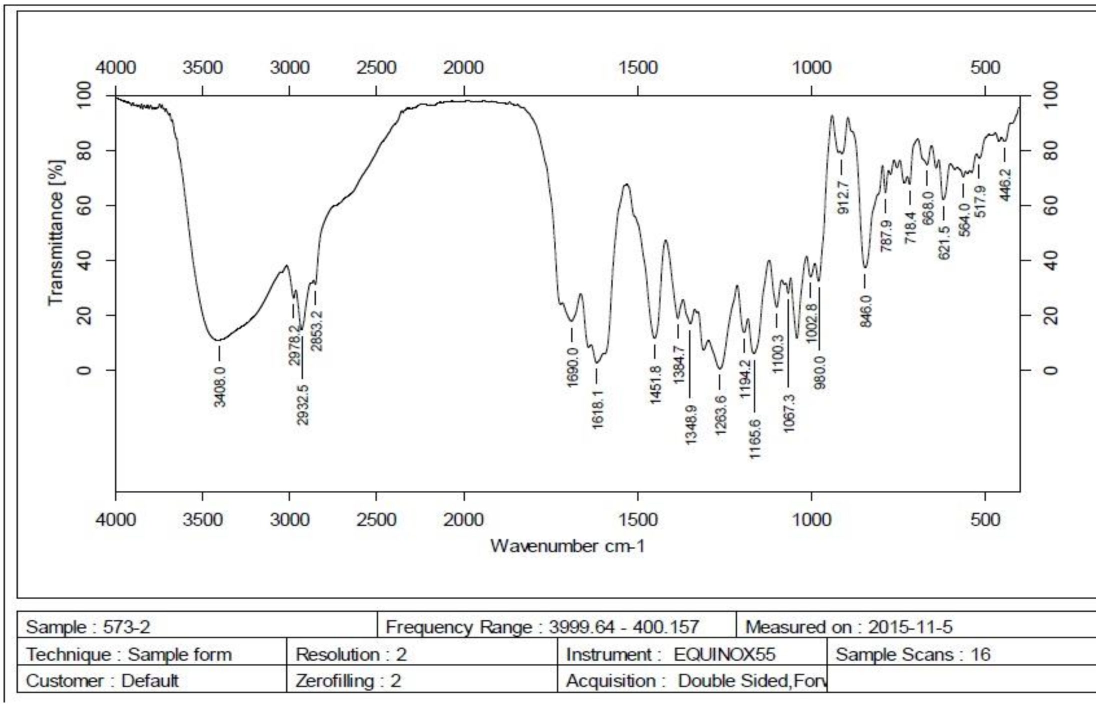

Figure S43 IR (KBr disc) spectrum of penicimenolide E (**5**)

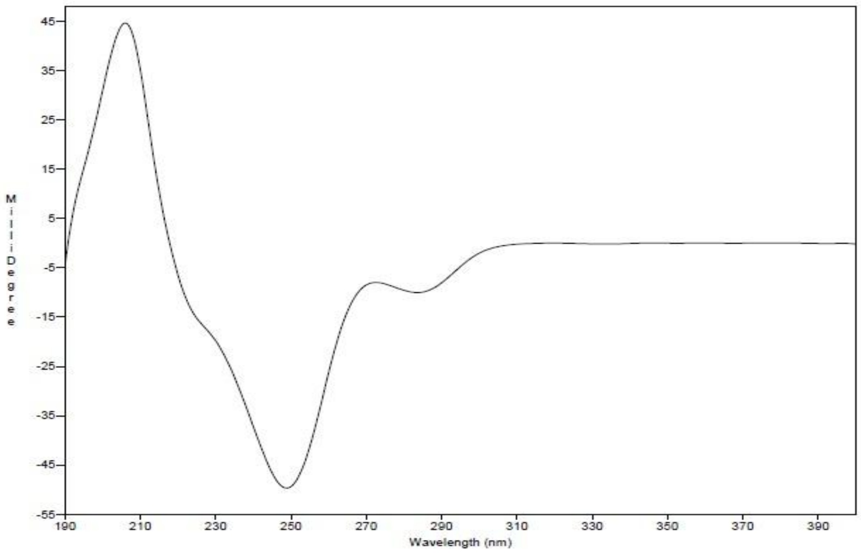

Figure S44 CD spectrum of penicimenolide E (**5**) in MeOH

Spectrum Peak Pick Report

FIELD FIELD TEXT

Data Set: 没有

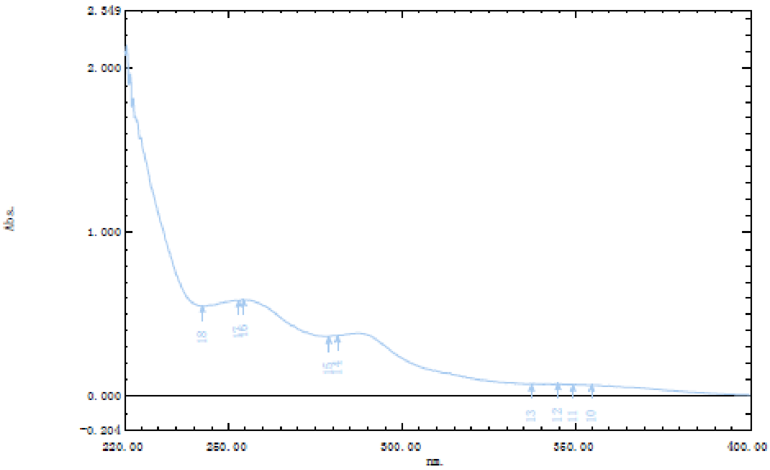

测定属性
波长范围 (nm.): 220.00到400.00
扫描速度: 高速
采样间隔: 0.2
自动采样间隔: 启用
扫描模式: 单一的

试样准备属性
重量: 0.5
体积 : 10
稀释:
光程长: 407
附加信息:

仪器属性
仪器类型: UV-1700
测定方式: 吸收值
狭缝宽: 1.0 nm
光源改变波长: 340.8 nm
S/R 转换: 标准

No.	P/V	Wavelength	Abs.	描述
1	P	355.80	.069	
2	P	350.00	.073	
3	P	345.60	.076	
4	P	339.00	.077	
5	P	287.60	.386	
6	P	280.40	.369	
7	P	255.20	.587	
8	P	253.60	.588	
9	P	252.00	.585	
10	V	354.40	.067	
11	V	349.20	.072	
12	V	344.80	.074	
13	V	337.40	.074	
14	V	281.20	.368	
15	V	278.80	.364	
16	V	254.40	.585	
17	V	252.80	.582	
18	V	242.40	.548	

Figure S45 UV spectrum of penicimenolide E (**5**) in MeOH

Elemental Composition Report **Page 1**

Single Mass Analysis
Tolerance = 5.0 mDa / DBE: min = -1.5, max = 50.0
Element prediction: Off
Number of isotope peaks used for i-FIT = 3

Monoisotopic Mass, Even Electron Ions
436 formula(e) evaluated with 6 results within limits (up to 10 best isotopic matches for each mass)
Elements Used:
C: 0-500 H: 0-1000 N: 0-10 O: 0-200
573-2
20150032337 77 (0.632)

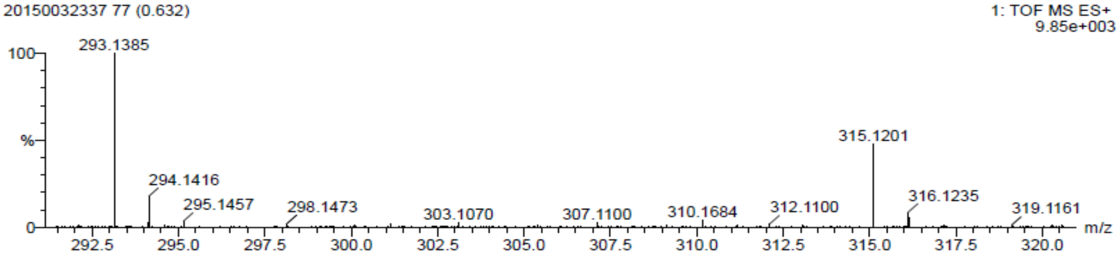

		mDa	PPM	DBE				
Minimum:				-1.5				
Maximum:		5.0	10.0	50.0				

Mass	Calc. Mass	mDa	PPM	DBE	i-FIT	Norm	Conf(%)	Formula
293.1385	293.1389	-0.4	-1.4	6.5	187.0	0.078	92.48	C16 H21 O5
	293.1402	-1.7	-5.8	11.5	189.6	2.702	6.70	C17 H17 N4 O
	293.1362	2.3	7.8	7.5	192.1	5.198	0.55	C12 H17 N6 O3
	293.1349	3.6	12.3	2.5	192.9	5.993	0.25	C11 H21 N2 O7
	293.1421	-3.6	-12.3	-1.5	196.7	9.741	0.01	C5 H21 N6 O8
	293.1434	-4.9	-16.7	3.5	196.9	10.005	0.00	C6 H17 N10 O4

Figure S46 HR-ESI-MS spectrum of penicimenolide E (**5**)

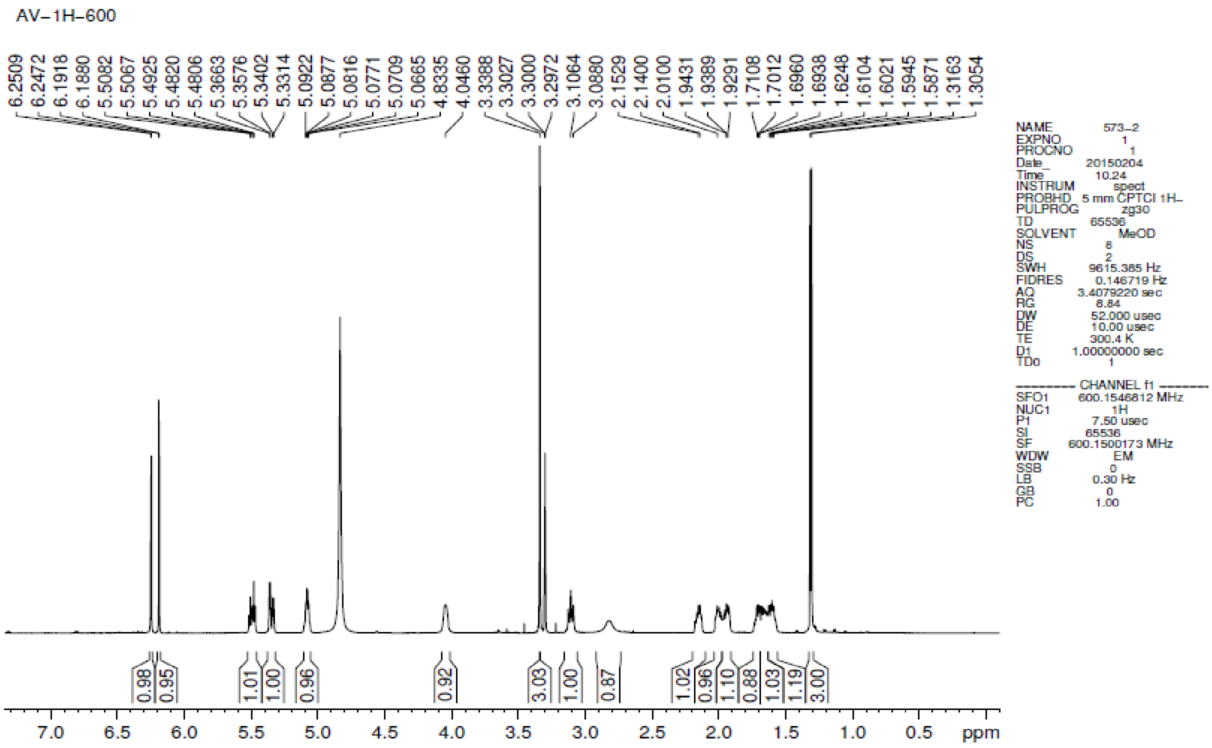

Figure S47 ^{1}H-NMR spectrum of **5** (600MHz, in CD_3OD)

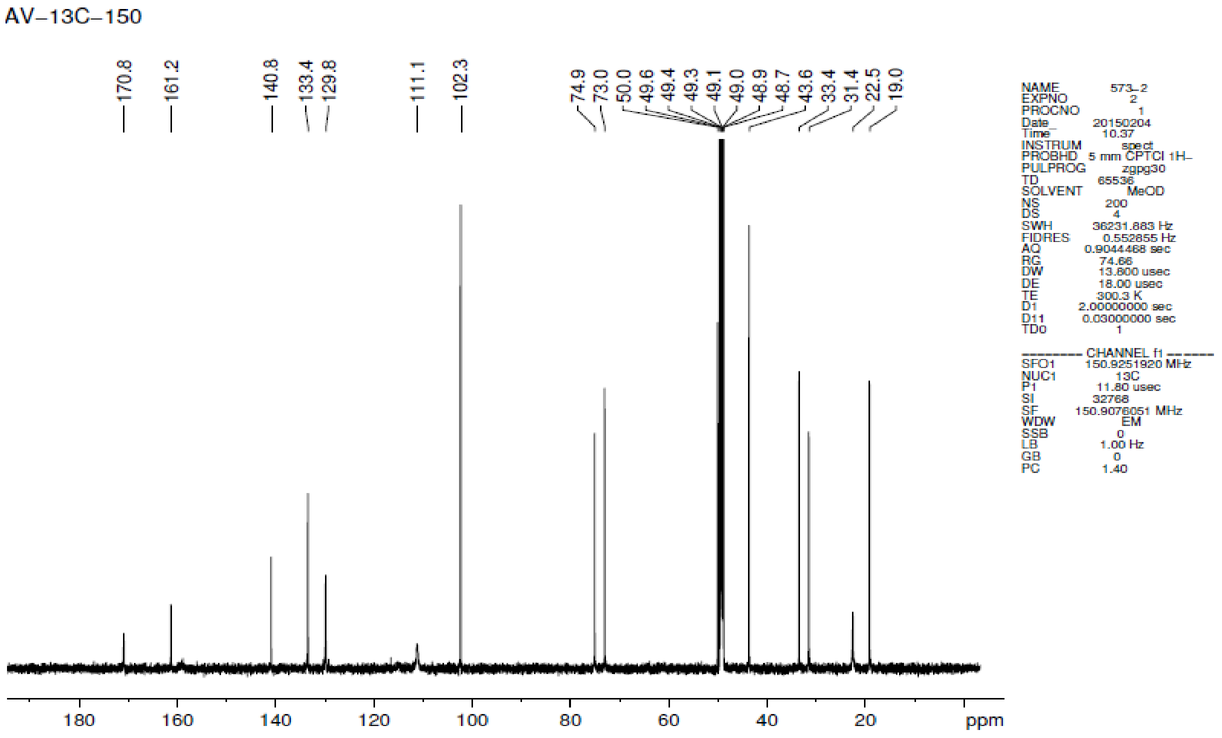

Figure S48 ^{13}C-NMR spectrum of **5** (150MHz, in CD_3OD)

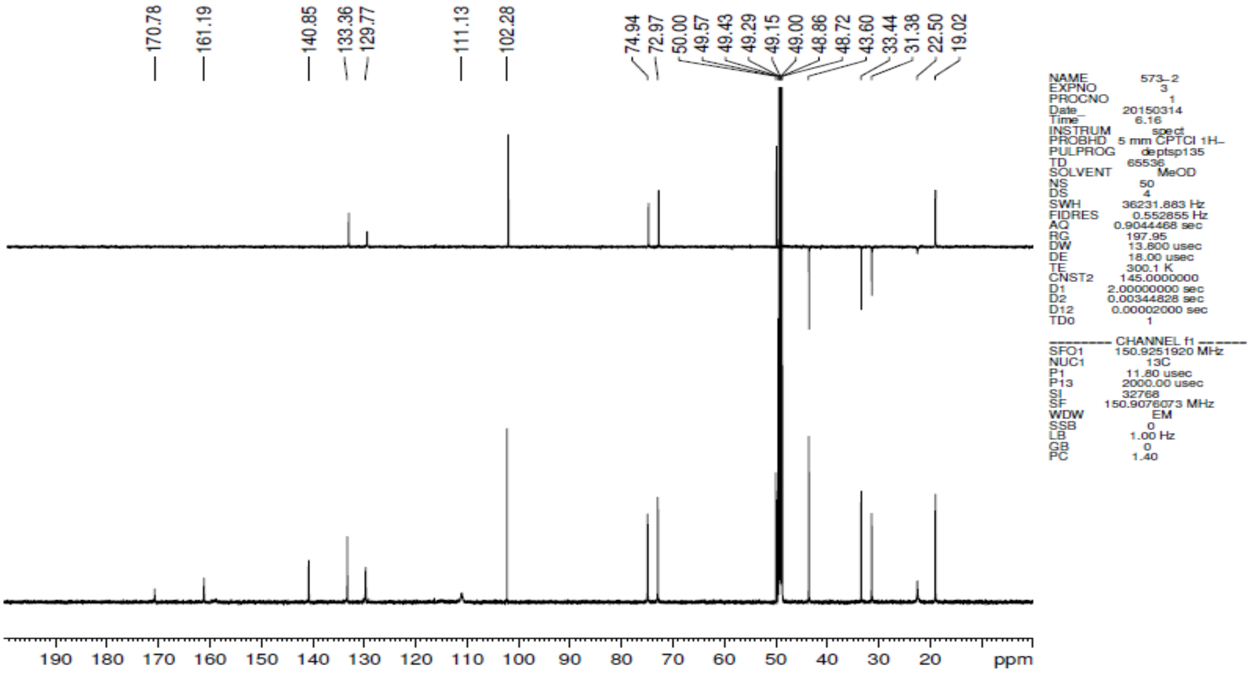

Figure S49 ^{13}C-NMR and DEPT 135 spectra of **5** (150MHz, in CD_3OD)

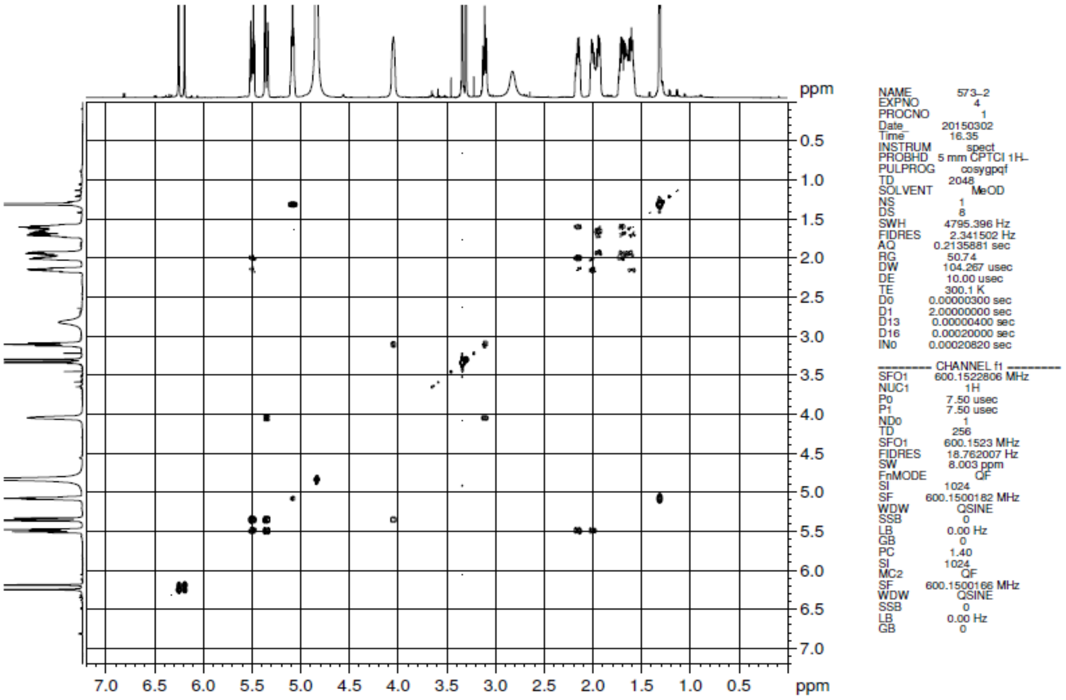

Figure S50 ^{1}H-^{1}H COSY spectrum of **5** (600MHz, in CD_3OD)

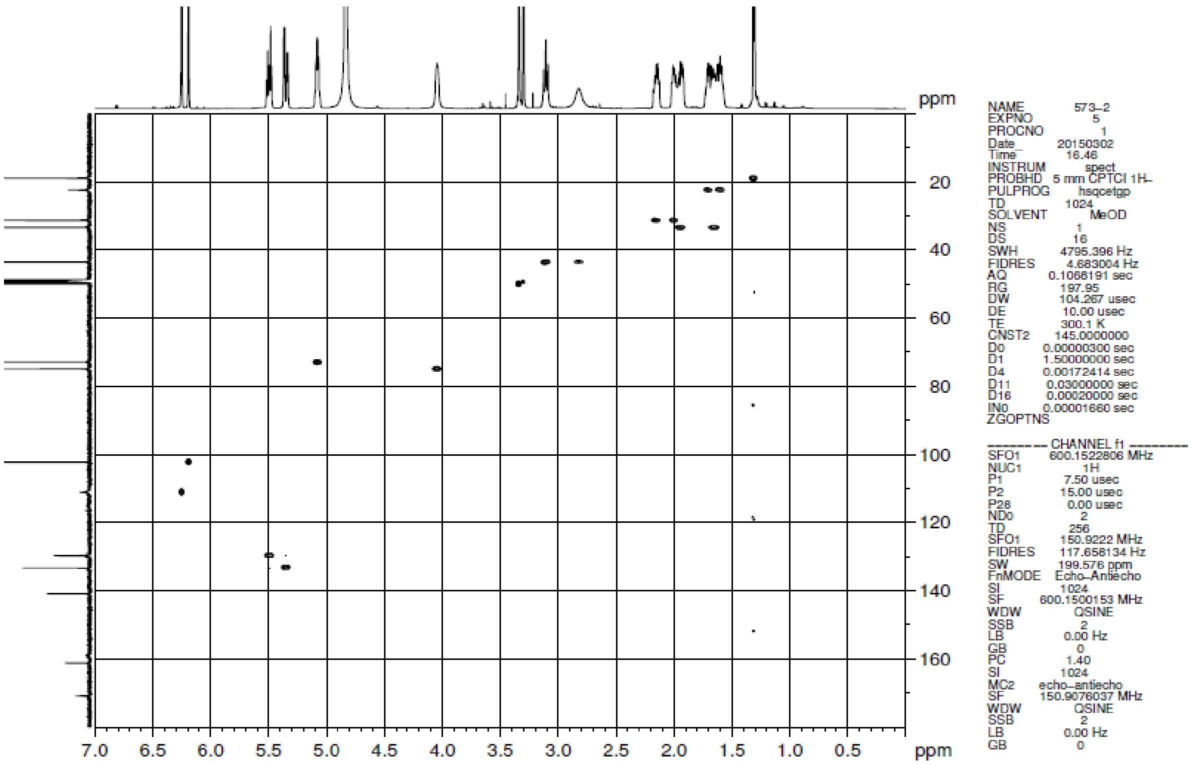

Figure S51 HSQC spectrum of **5** (600MHz, in CD_3OD)

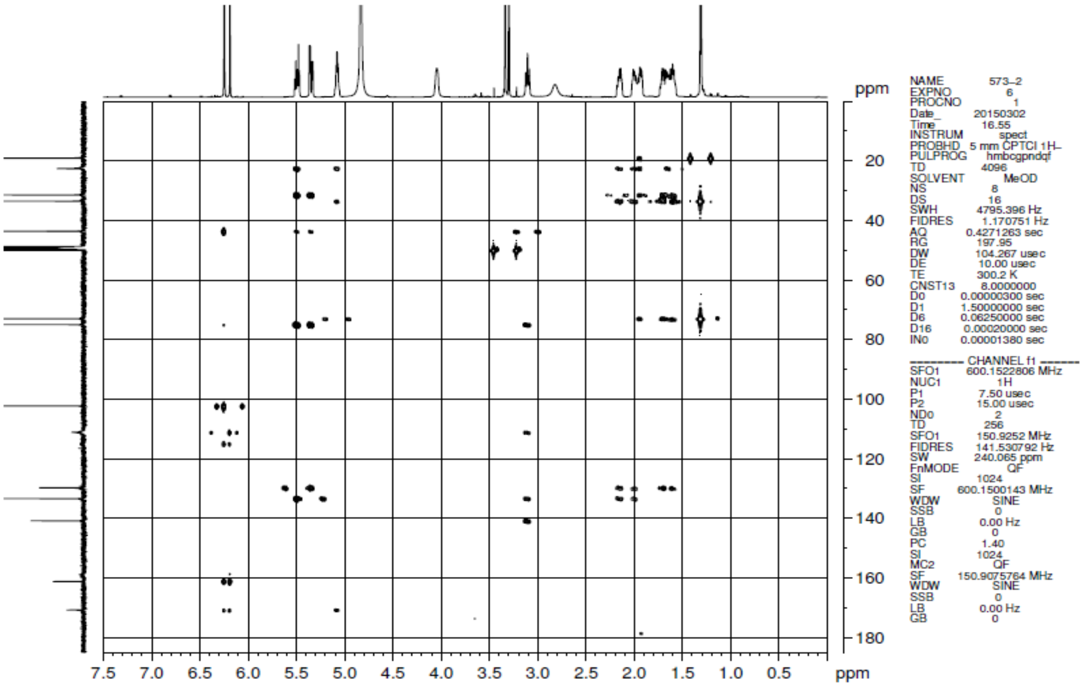

Figure S52 HMBC spectrum of **5** (600MHz, in CD_3OD)

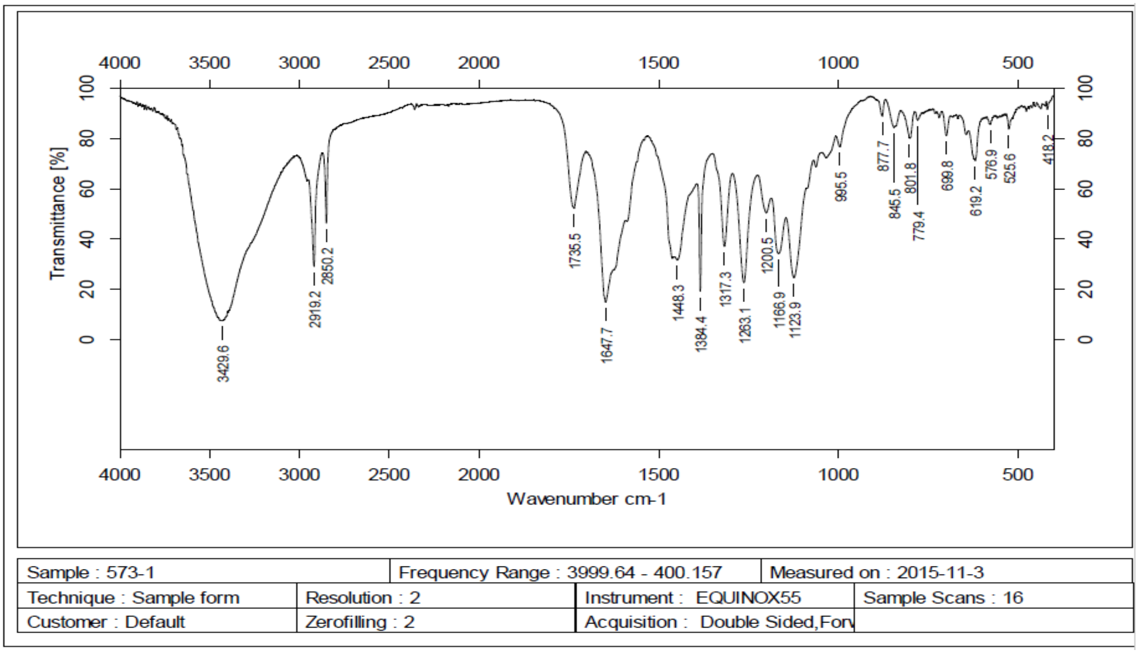

Figure S53 IR (KBr disc) spectrum of penicimenolide F (**6**)

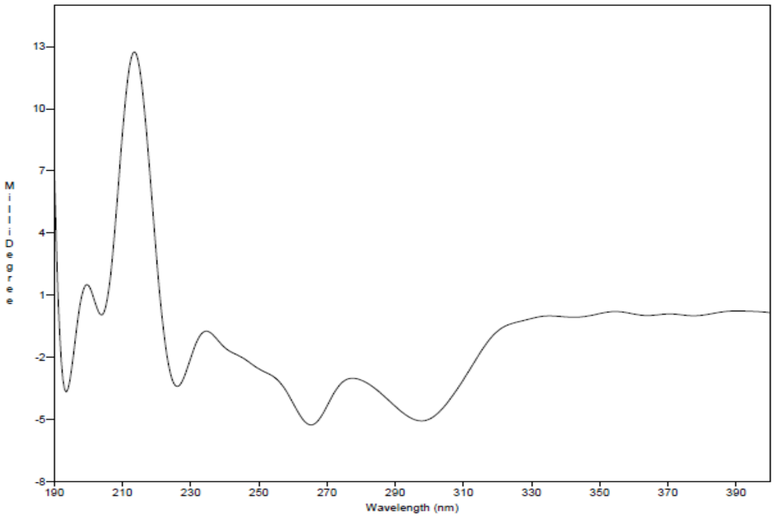

Figure S54 CD spectrum of penicimenolide F (**6**) in MeOH

Spectrum Peak Pick Report

FIELD FIELD TEXT

Data Set: 没有

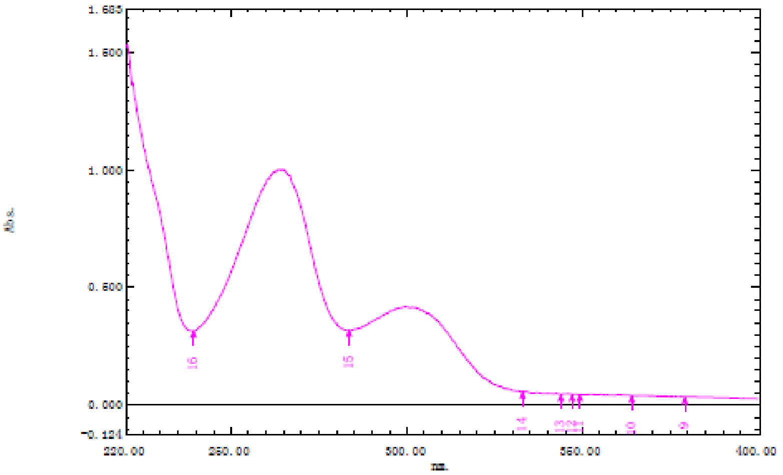

测定属性
波长范围 (nm.): 220. 00到400. 00
扫描速度: 高速
采样间隔: 0. 2
自动采样间隔: 启用
扫描模式: 单一的

试样准备属性
重量: 0. 5
体积 : 10
稀释:
光程长: 407
附加信息:

仪器属性
仪器类型: UV-1700
测定方式: 吸收值
狭缝宽: 1. 0 nm
光源改变波长: 340. 8 nm
S/R 转换: 标准

No.	P/V	Wavelength	Abs.	描述
1	P	380.60	.035	
2	P	364.60	.040	
3	P	355.20	.044	
4	P	348.00	.046	
5	P	345.60	.048	
6	P	333.80	.057	
7	P	299.40	.418	
8	P	263.80	1.003	
9	V	379.00	.034	
10	V	364.00	.040	
11	V	349.00	.041	
12	V	347.00	.044	
13	V	343.80	.045	
14	V	333.00	.052	
15	V	283.60	.316	
16	V	239.20	.314	

Figure S55 UV spectrum of penicimenolide F (**6**) in MeOH

Elemental Composition Report

Page 1

Single Mass Analysis

Tolerance = 5.0 PPM / DBE: min = -1.5, max = 30.0
Element prediction: Off
Number of isotope peaks used for i-FIT = 3

Monoisotopic Mass, Even Electron Ions
186 formula(e) evaluated with 1 results within limits (up to 20 closest results for each mass)
Elements Used:
C: 0-50 H: 0-100 N: 0-3 O: 0-30
573-1
20150617-05 65 (0.531) Cm (63:65) 1: TOF MS ES+ 6.61e+005

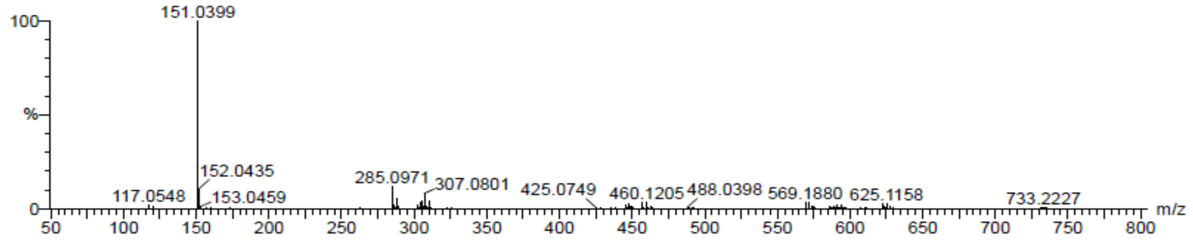

Minimum:				-1.5				
Maximum:		5.0	5.0	30.0				
Mass	Calc. Mass	mDa	PPM	DBE	i-FIT	Norm	Conf(%)	Formula
285.0971	285.0974	-0.3	-1.1	5.5	288.6	n/a	n/a	C13 H17 O7

Figure S56 HR-ESI-MS spectrum of penicimenolide F (**6**)

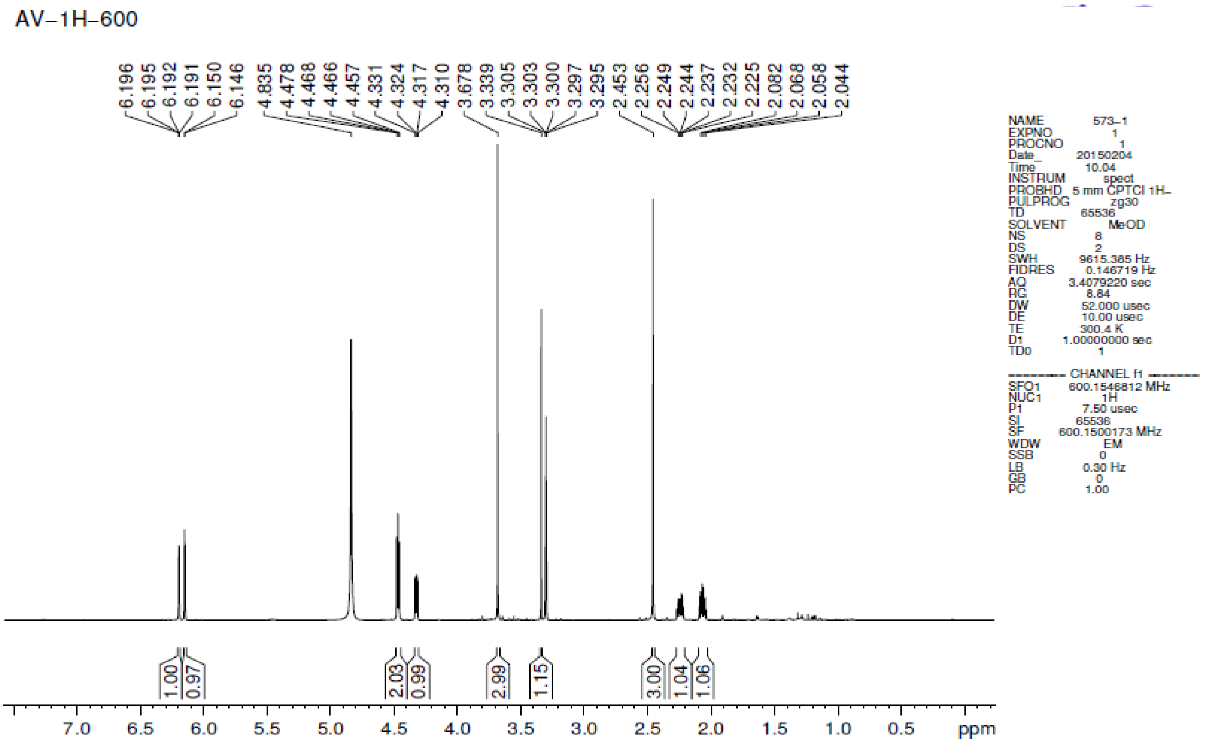

Figure S57 ^{1}H-NMR spectrum of **6** (600MHz, in CD_3OD)

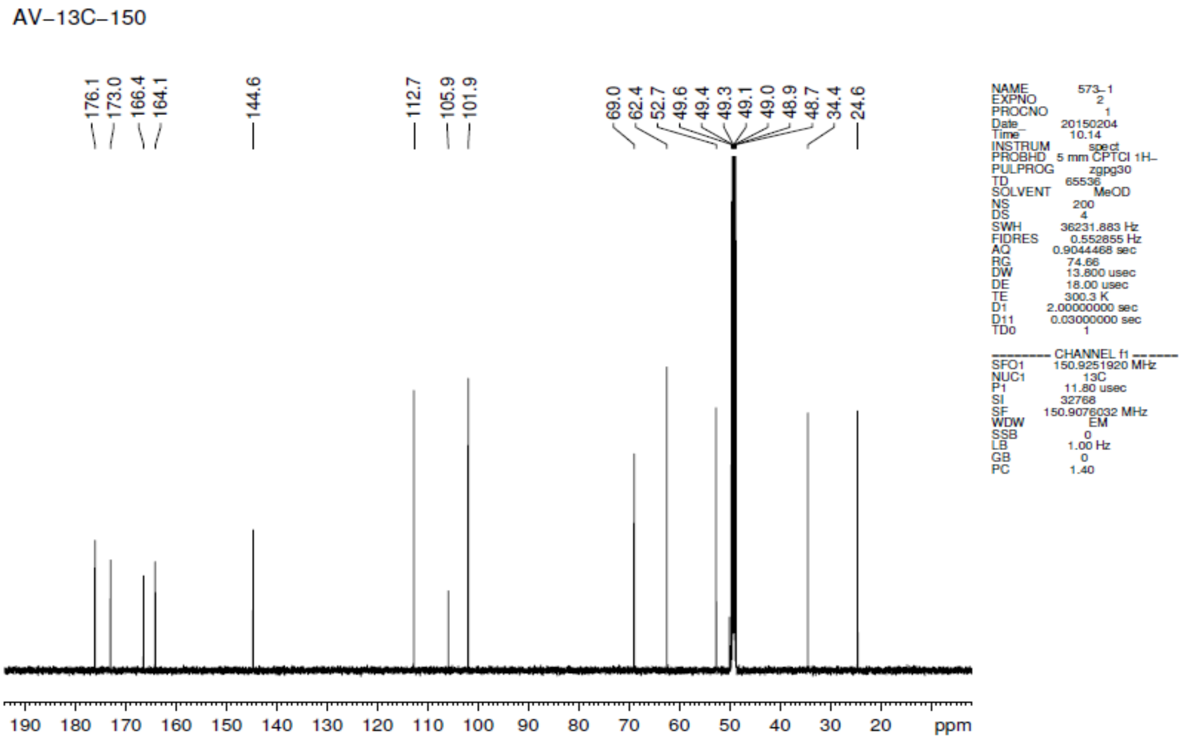

Figure S58 ^{13}C-NMR spectrum of **6** (150MHz, in CD_3OD)

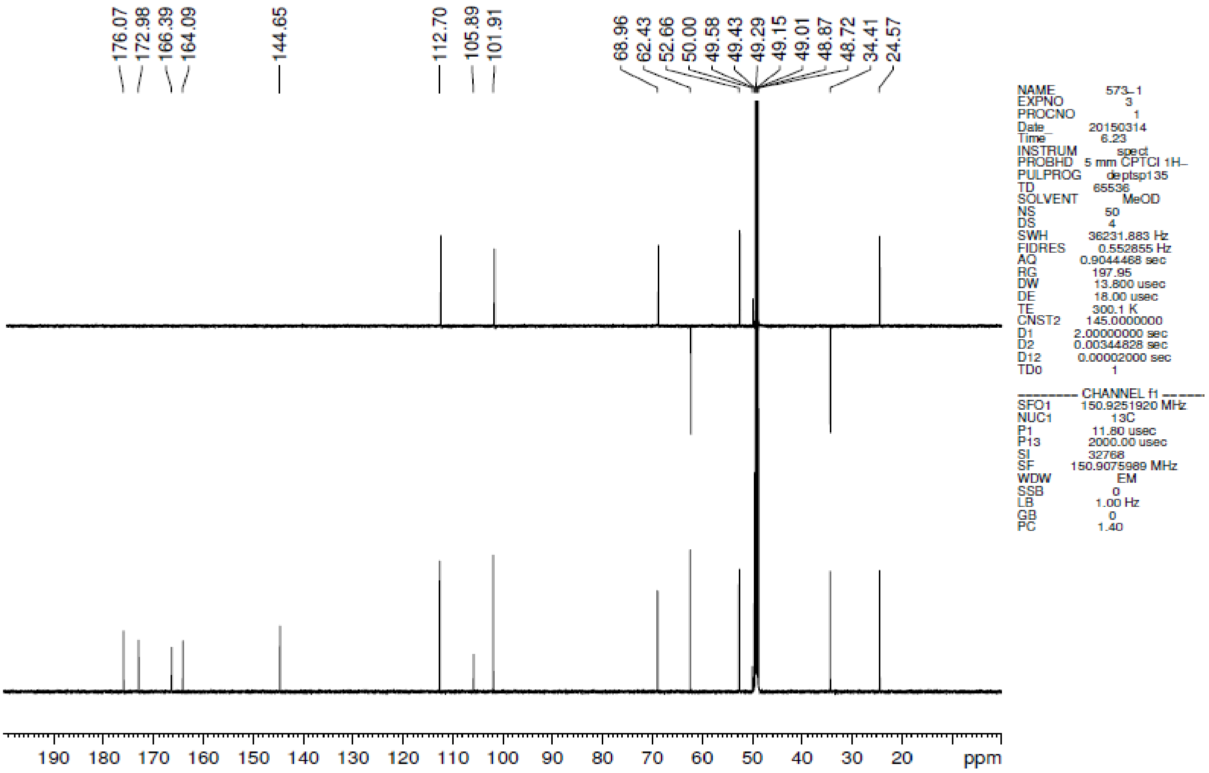

Figure S59 ^{13}C-NMR and DEPT 135 spectra of **6** (150MHz, in CD_3OD)

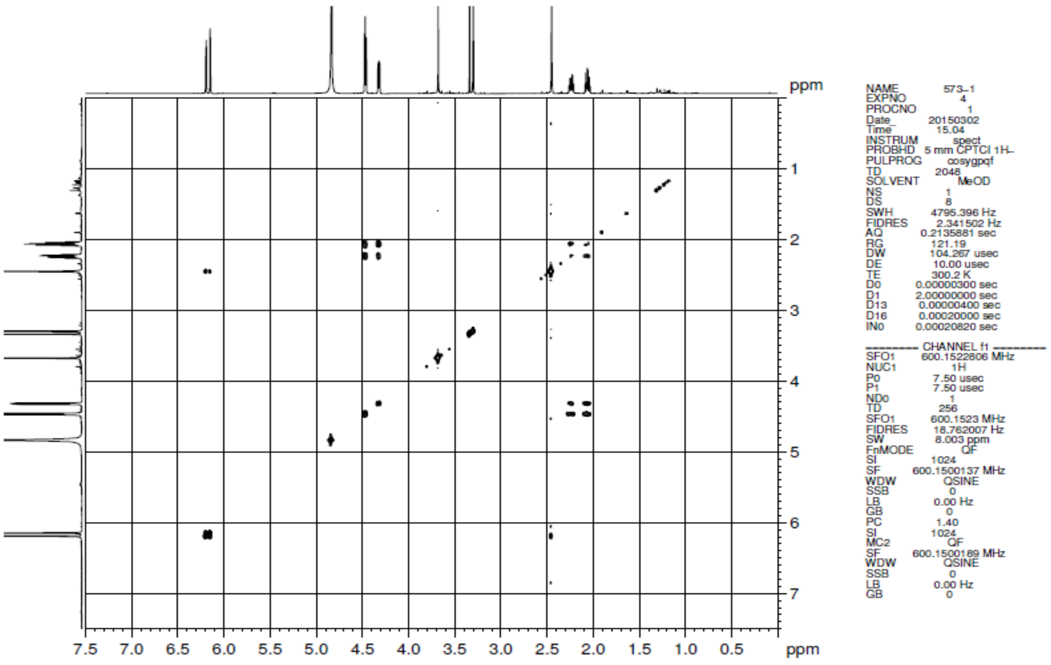

Figure S60 ^{1}H-^{1}H COSY spectrum of **6** (600MHz, in CD_3OD)

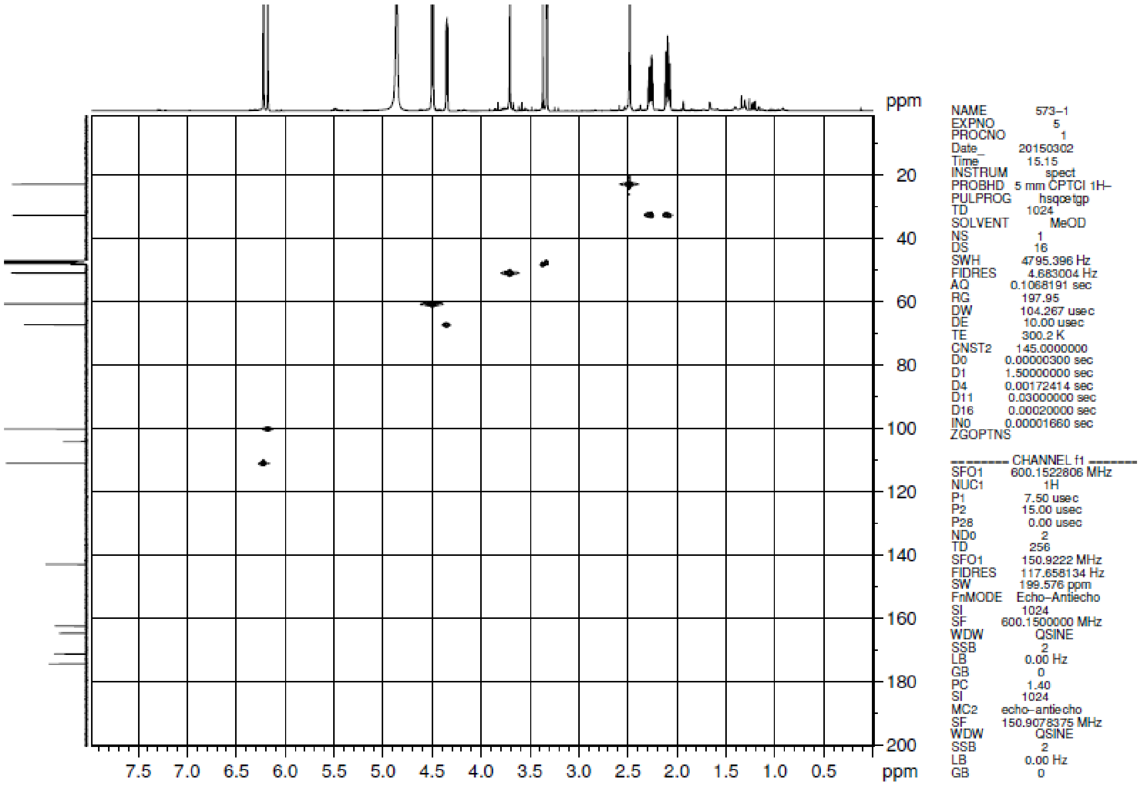

Figure S61 HSQC spectrum of **6** (600MHz, in CD_3OD)

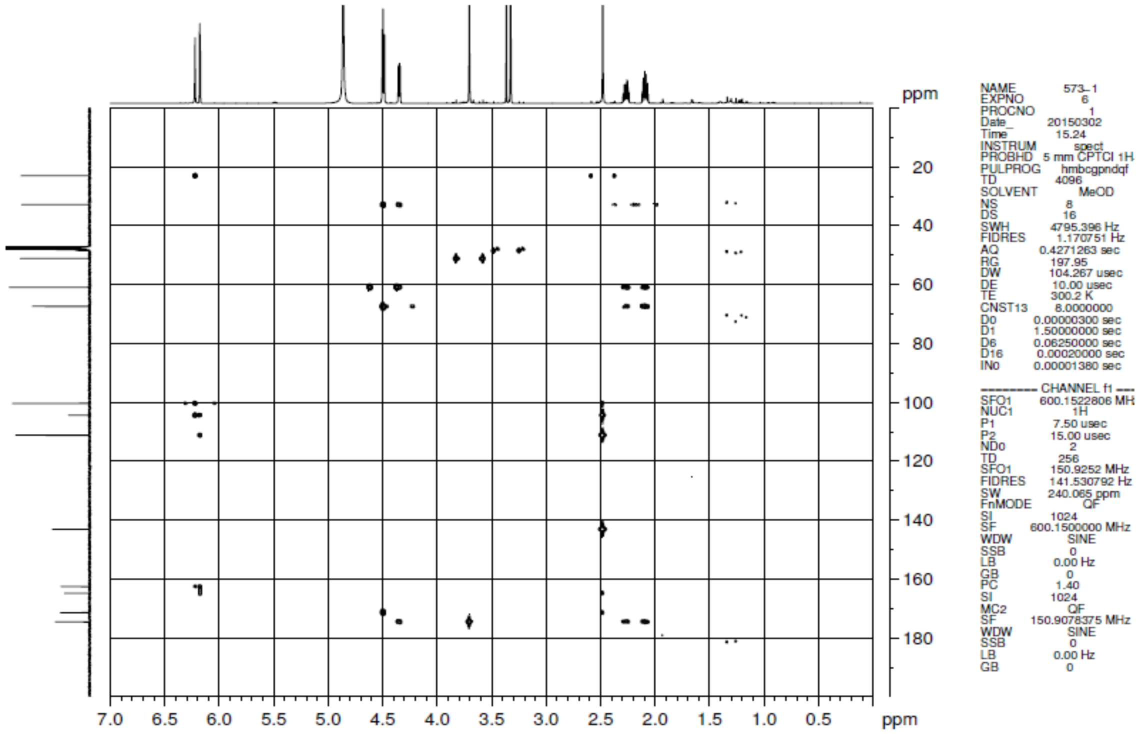

Figure S62 HMBC spectrum of **6** (600MHz, in CD_3OD)

4. NMR data of compound 8

Table S1 NMR data of compound **8** in CD_3OD

position	δ_C	δ_H (*J* in Hz)
1	172.6	
3	74.7	5.10, m
4	32.9	1.62, m
		1.64, m
5	22.3	1.46, m
		1.47, m
6	28.3	1.47, m
		1.49, m
7	22.3	2.00, m
		1.79, m
8	42.7	2.67, m
		2.33, m
9	211.8	
10	51.8	4.68, d (18.7)
		3.78, d (18.5)
11	140.4	
12	113.9	6.09, d (2.3)
13	163.9	
14	103.0	6.24, d (2.3)
15	166.7	
16	106.8	
17	19.6	1.28, d (6.2)

5. ^{1}H-NMR chemical shift differences for the MTPA esters of 3, 5 and 6

Table S2 ^{1}H-NMR chemical shift differences between MTPA esters of **3a** and **3b** (Pyridine-d_5)

position	**3**	**3a**	**3b**	**Δδ**
	δ_H	δ_H	δ_H	
7	5.928	5.761	5.778	-0.017
3´	1.627	1.581	1.511	0.069

Table S3 ^{1}H-NMR chemical shift differences between MTPA esters of **5a** and **5b** (Pyridine-d_5)

position	**5**	**5a**	**5b**	**Δδ**
	δ_H	δ_H	δ_H	
4	2.052	1.705	1.629	0.076
	-	-	-	-
5	1.768	1.507	1.466	0.041
	-	-	-	-
6	2.247	2.025	1.937	0.05
	2.120	1.860	1.747	0.05
7	4.754	-	-	-
8	5.598	5.537	5.406	0.131
9	3.876	-	-	-
10	3.461	3.364	3.409	-0.306
17	1.408	1.225	1.073	0.152

Table S4 ^{1}H-NMR chemical shift differences between MTPA esters of **6a** and **6b** (Pyridine-d_5)

position	**6**	**6a**	**6b**	**Δδ**
	δ_H	δ_H	δ_H	
1´	4.792	4.555	4.440	0.115
	4.756	4.518	4.394	0.120
2´	2.496	2.538	2.476	0.06
	2.358	2.456	2.423	0.03
5´	3.69	3.709	3.775	-0.07

6. Single crystal X-ray diffraction analysis of compound 10

data_574-4-4

_audit_creation_date 2015-10-17

_audit_creation_method

;

Olex2 1.2

(compiled 2015.09.30 svn.r3233 for OlexSys, GUI svn.r5103)

;

_publ_contact_author_address ?

_publ_contact_author_email ?

_publ_contact_author_name ''

_publ_contact_author_phone ?

_publ_section_references

;

Dolomanov, O.V., Bourhis, L.J., Gildea, R.J, Howard, J.A.K. & Puschmann, H.

(2009), J. Appl. Cryst. 42, 339-341.

Sheldrick, G.M. (2008). Acta Cryst. A64, 112-122.

Sheldrick, G.M. (2015). Acta Cryst. C71, 3-8.

;

_chemical_name_common ?

_chemical_name_systematic

;

?

;

_chemical_formula_moiety 'C17 H22 O6'

_chemical_formula_sum 'C17 H22 O6'

_chemical_formula_weight 322.35

_chemical_absolute_configuration ad

_chemical_melting_point ?

_chemical_oxdiff_formula 'C H O'

```
loop_
  _atom_type_symbol
  _atom_type_description
  _atom_type_scat_dispersion_real
  _atom_type_scat_dispersion_imag
  _atom_type_scat_source
 'C' 'C' 0.0181 0.0091 'International Tables Vol C Tables 4.2.6.8 and 6.1.1.4'
 'H' 'H' 0.0000 0.0000 'International Tables Vol C Tables 4.2.6.8 and 6.1.1.4'
 'O' 'O' 0.0492 0.0322 'International Tables Vol C Tables 4.2.6.8 and 6.1.1.4'
_space_group_crystal_system       'monoclinic'
_space_group_IT_number            4
_space_group_name_H-M_alt         'P 1 21 1'
_space_group_name_Hall            'P 2yb'
loop_
  _space_group_symop_id
  _space_group_symop_operation_xyz
 1 'x, y, z'
 2 '-x, y+1/2, -z'
_cell_length_a                    5.05804(7)
_cell_length_b                    11.74745(14)
_cell_length_c                    13.77837(18)
_cell_angle_alpha                 90.00
_cell_angle_beta                  100.1922(13)
_cell_angle_gamma                 90.00
_cell_volume                      805.778(18)
_cell_formula_units_Z             2
_cell_measurement_reflns_used     4162
_cell_measurement_temperature     150
_cell_measurement_theta_max       75.1230
_cell_measurement_theta_min       3.2430
```

_exptl_absorpt_coefficient_mu 0.836

_exptl_absorpt_correction_T_max 1.00000

_exptl_absorpt_correction_T_min 0.54692

_exptl_absorpt_correction_type multi-scan

_exptl_absorpt_process_details

;

CrysAlisPro, Agilent Technologies,

Version 1.171.36.32 (release 02-08-2013 CrysAlis171 .NET)

(compiled Aug 2 2013,16:46:58)

Empirical absorption correction using spherical harmonics,

implemented in SCALE3 ABSPACK scaling algorithm.

;

_exptl_crystal_colour ''

_exptl_crystal_density_diffrn 1.329

_exptl_crystal_density_meas ?

_exptl_crystal_density_method 'not measured'

_exptl_crystal_description ''

_exptl_crystal_F_000 344

_exptl_crystal_size_max ?

_exptl_crystal_size_mid ?

_exptl_crystal_size_min ?

_exptl_special_details

;

?

;

_diffrn_reflns_av_R_equivalents 0.0167

_diffrn_reflns_av_unetI/netI 0.0140

_diffrn_reflns_limit_h_max 6

_diffrn_reflns_limit_h_min -5

_diffrn_reflns_limit_k_max 14

_diffrn_reflns_limit_k_min -14

_diffrn_reflns_limit_l_max 16

_diffrn_reflns_limit_l_min -17

_diffrn_reflns_number 5093

_diffrn_reflns_theta_full 75.46

_diffrn_reflns_theta_max 75.46

_diffrn_reflns_theta_min 3.26

_diffrn_ambient_temperature 150

_diffrn_detector_area_resol_mean 10.6525

_diffrn_measured_fraction_theta_full 0.971

_diffrn_measured_fraction_theta_max 0.971

_diffrn_measurement_details

;

```
#__ type_ start__ end____ width___ exp.time_
  1 omega  -90.00  -10.00   1.0000    1.0000
omega____ theta____ kappa____ phi______ frames
    -      -39.3998  -57.0000   30.0000 80
#__ type_ start__ end____ width___ exp.time_
  2 omega  -50.00   34.00   1.0000    1.0000
omega____ theta____ kappa____ phi______ frames
    -      -39.3998   38.0000   60.0000 84
#__ type_ start__ end____ width___ exp.time_
  3 omega  -96.00  -32.00   1.0000    1.0000
omega____ theta____ kappa____ phi______ frames
    -      -39.3998  -38.0000 -120.0000 64
#__ type_ start__ end____ width___ exp.time_
  4 omega   42.00   79.00   1.0000    1.0000
omega____ theta____ kappa____ phi______ frames
    -       39.3998  -99.0000  -90.0000 37
```

#__ type_ start__ end_____ width___ exp.time_

5 omega 17.00 43.00 1.0000 1.0000

omega____ theta____ kappa____ phi______ frames

- 39.3998 57.0000 -90.0000 26

#__ type_ start__ end_____ width___ exp.time_

6 omega -27.00 56.00 1.0000 1.0000

omega____ theta____ kappa____ phi______ frames

- 39.3998 -38.0000 -180.0000 83

#__ type_ start__ end_____ width___ exp.time_

7 omega 81.00 178.00 1.0000 1.0000

omega____ theta____ kappa____ phi______ frames

- 111.3657 30.0000 60.0000 97

#__ type_ start__ end_____ width___ exp.time_

8 omega 120.00 151.00 1.0000 1.0000

omega____ theta____ kappa____ phi______ frames

- 111.3657 45.0000 120.0000 31

#__ type_ start__ end_____ width___ exp.time_

9 omega 82.00 177.00 1.0000 1.0000

omega____ theta____ kappa____ phi______ frames

- 111.3657 30.0000 -30.0000 95

#__ type_ start__ end_____ width___ exp.time_

10 omega 83.00 108.00 1.0000 1.0000

omega____ theta____ kappa____ phi______ frames

- 111.3657 -94.0000 -90.0000 25

#__ type_ start__ end_____ width___ exp.time_

11 omega 30.00 102.00 1.0000 1.0000

omega____ theta____ kappa____ phi______ frames

- 111.3657 -94.0000 120.0000 72

#__ type_ start__ end_____ width___ exp.time_

12 omega 53.00 95.00 1.0000 1.0000

omega____ theta____ kappa____ phi______ frames

- 111.3657 -94.0000 0.0000 42

#__ type_ start__ end____ width___ exp.time_

13 omega 35.00 62.00 1.0000 1.0000

omega____ theta____ kappa____ phi______ frames

- 111.3657 -94.0000 -90.0000 27

#__ type_ start__ end____ width___ exp.time_

14 omega 30.00 111.00 1.0000 1.0000

omega____ theta____ kappa____ phi______ frames

- 111.3657 -98.0000 -142.0000 81

#__ type_ start__ end____ width___ exp.time_

15 omega 81.00 175.00 1.0000 1.0000

omega____ theta____ kappa____ phi______ frames

- 111.3657 61.0000 -150.0000 94

#__ type_ start__ end____ width___ exp.time_

16 omega 33.00 89.00 1.0000 1.0000

omega____ theta____ kappa____ phi______ frames

- 111.3657 -30.0000 150.0000 56

#__ type_ start__ end____ width___ exp.time_

17 omega 34.00 81.00 1.0000 1.0000

omega____ theta____ kappa____ phi______ frames

- 111.3657 -30.0000 -90.0000 47

#__ type_ start__ end____ width___ exp.time_

18 omega 88.00 132.00 1.0000 1.0000

omega____ theta____ kappa____ phi______ frames

- 111.3657 45.0000 90.0000 44

#__ type_ start__ end____ width___ exp.time_

19 omega 78.00 109.00 1.0000 1.0000

omega____ theta____ kappa____ phi______ frames

- 111.3657 -94.0000 30.0000 31

#__ type_ start__ end____ width___ exp.time_

20 omega 104.00 167.00 1.0000 1.0000

omega____ theta____ kappa____ phi______ frames

\- 111.3657 111.0000 60.0000 63

;

_diffrn_measurement_device_type 'SuperNova, Dual, Cu at zero, Atlas'

_diffrn_measurement_method '\w scans'

_diffrn_orient_matrix_UB_11 0.2563312000

_diffrn_orient_matrix_UB_12 0.0018305000

_diffrn_orient_matrix_UB_13 0.0792356000

_diffrn_orient_matrix_UB_21 -0.0541486000

_diffrn_orient_matrix_UB_22 -0.1237051000

_diffrn_orient_matrix_UB_23 0.0279951000

_diffrn_orient_matrix_UB_31 0.1646206000

_diffrn_orient_matrix_UB_32 -0.0434483000

_diffrn_orient_matrix_UB_33 -0.0764728000

_diffrn_radiation_monochromator mirror

_diffrn_radiation_type CuK\a

_diffrn_radiation_wavelength 1.54184

_diffrn_source 'SuperNova (Cu) X-ray Source'

_diffrn_standards_decay_% ?

_diffrn_standards_interval_count ?

_diffrn_standards_interval_time ?

_diffrn_standards_number ?

_reflns_number_gt 3123

_reflns_number_total 3141

_reflns_odcompleteness_completeness 98.58

_reflns_odcompleteness_iscentric 1

_reflns_odcompleteness_theta 68.13

_reflns_threshold_expression >2sigma(I)

_computing_cell_refinement
;
CrysAlisPro, Agilent Technologies,
Version 1.171.36.32 (release 02-08-2013 CrysAlis171 .NET)
(compiled Aug 2 2013,16:46:58)
;
_computing_data_collection
;
CrysAlisPro, Agilent Technologies,
Version 1.171.36.32 (release 02-08-2013 CrysAlis171 .NET)
(compiled Aug 2 2013,16:46:58)
;
_computing_data_reduction
;
CrysAlisPro, Agilent Technologies,
Version 1.171.36.32 (release 02-08-2013 CrysAlis171 .NET)
(compiled Aug 2 2013,16:46:58)
;
_computing_molecular_graphics 'Olex2 (Dolomanov et al., 2009)'
_computing_publication_material 'Olex2 (Dolomanov et al., 2009)'
_computing_structure_refinement 'ShelXL (Sheldrick, 2015)'
_computing_structure_solution 'XS (Sheldrick, 2008)'
_refine_diff_density_max 0.202
_refine_diff_density_min -0.199
_refine_diff_density_rms 0.038
_refine_ls_abs_structure_details 'Flack H D (1983), Acta Cryst. A39, 876-881'
_refine_ls_abs_structure_Flack 0.08(10)
_refine_ls_extinction_coef ?
_refine_ls_extinction_method none
_refine_ls_goodness_of_fit_ref 0.971

_refine_ls_hydrogen_treatment constr

_refine_ls_matrix_type full

_refine_ls_number_parameters 212

_refine_ls_number_reflns 3141

_refine_ls_number_restraints 1

_refine_ls_R_factor_all 0.0336

_refine_ls_R_factor_gt 0.0333

_refine_ls_restrained_S_all 0.971

_refine_ls_shift/su_max 0.000

_refine_ls_shift/su_mean 0.000

_refine_ls_structure_factor_coef Fsqd

_refine_ls_weighting_details

'calc w=1/[\s^2^(Fo^2^)+(0.1000P)^2^+0.0000P] where P=(Fo^2^+2Fc^2^)/3'

_refine_ls_weighting_scheme calc

_refine_ls_wR_factor_gt 0.1000

_refine_ls_wR_factor_ref 0.1007

_refine_special_details

;

Refinement of F^2^ against ALL reflections. The weighted R-factor wR and goodness of fit S are based on F^2^, conventional R-factors R are based on F, with F set to zero for negative F^2^. The threshold expression of F^2^ > 2sigma(F^2^) is used only for calculating R-factors(gt) etc. and is not relevant to the choice of reflections for refinement. R-factors based on F^2^ are statistically about twice as large as those based on F, and R-factors based on ALL data will be even larger.

;

_olex2_refinement_description

;

1. Fixed Uiso

At 1.2 times of:

All C(H) groups, All C(H,H) groups
At 1.5 times of:
All C(H,H,H) groups, All O(H) groups
2.a Ternary CH refined with riding coordinates:
C7(H7), C3(H3)
2.b Secondary CH2 refined with riding coordinates:
C8(H8A,H8B), C10(H10A,H10B), C5(H5A,H5B), C4(H4A,H4B), C6(H6A,H6B)
2.c Aromatic/amide H refined with riding coordinates:
C14(H14), C12(H12)
2.d Idealised Me refined as rotating group:
C18(H18A,H18B,H18C), C17(H17A,H17B,H17C)
2.e Idealised tetrahedral OH refined as rotating group:
O1(H1), O6(H6)
;
_atom_sites_solution_hydrogens geom
_atom_sites_solution_primary direct
_atom_sites_solution_secondary difmap
loop_
_atom_site_label
_atom_site_type_symbol
_atom_site_fract_x
_atom_site_fract_y
_atom_site_fract_z
_atom_site_U_iso_or_equiv
_atom_site_adp_type
_atom_site_occupancy
_atom_site_calc_flag
_atom_site_disorder_assembly
_atom_site_disorder_group
_atom_site_refinement_flags_posn

O5 O 0.09976(16) 0.24159(8) 0.14651(7) 0.0257(2) Uani 1 d . . .

O1 O -0.03984(19) 0.60821(9) 0.33759(8) 0.0343(2) Uani 1 d . . .

H1 H -0.0778 0.5522 0.3704 0.051 Uiso 1 calc . . GR

O6 O 0.4603(2) 0.70790(8) 0.09151(7) 0.0342(2) Uani 1 d . . .

H6 H 0.4091 0.7733 0.1045 0.051 Uiso 1 calc . . GR

O3 O 0.29093(18) 0.28453(8) 0.36167(6) 0.0267(2) Uani 1 d . . .

O4 O 0.27681(18) -0.07692(7) 0.10716(6) 0.0260(2) Uani 1 d . . .

O2 O 0.0163(2) 0.41098(10) 0.41253(8) 0.0377(3) Uani 1 d . . .

C11 C 0.4129(2) 0.43710(10) 0.21309(8) 0.0200(2) Uani 1 d . . .

C16 C 0.2390(2) 0.46321(10) 0.28110(8) 0.0209(2) Uani 1 d . . .

C1 C 0.1699(2) 0.38533(11) 0.35630(9) 0.0239(2) Uani 1 d . . .

C9 C 0.3413(2) 0.22805(10) 0.16438(7) 0.0178(2) Uani 1 d . . .

C14 C 0.1935(3) 0.65669(10) 0.21330(10) 0.0277(3) Uani 1 d . . .

H14 H 0.1136 0.7300 0.2111 0.033 Uiso 1 calc . . R

C8 C 0.4613(2) 0.11390(9) 0.14335(8) 0.0210(2) Uani 1 d . . .

H8A H 0.6518 0.1115 0.1762 0.025 Uiso 1 calc . . R

H8B H 0.4562 0.1066 0.0715 0.025 Uiso 1 calc . . R

C12 C 0.4767(2) 0.52030(10) 0.15095(9) 0.0221(2) Uani 1 d . . .

H12 H 0.5927 0.5022 0.1060 0.026 Uiso 1 calc . . R

C7 C 0.3119(2) 0.01291(9) 0.17901(8) 0.0205(2) Uani 1 d . . .

H7 H 0.1303 0.0396 0.1883 0.025 Uiso 1 calc . . R

C10 C 0.5363(2) 0.32141(9) 0.20361(8) 0.0198(2) Uani 1 d . . .

H10A H 0.6701 0.3285 0.1595 0.024 Uiso 1 calc . . R

H10B H 0.6338 0.2982 0.2693 0.024 Uiso 1 calc . . R

C3 C 0.2421(2) 0.20521(12) 0.43870(8) 0.0266(3) Uani 1 d . . .

H3 H 0.0633 0.2220 0.4569 0.032 Uiso 1 calc . . R

C5 C 0.4987(2) 0.05022(11) 0.36038(8) 0.0250(2) Uani 1 d . . .

H5A H 0.5892 0.1182 0.3392 0.030 Uiso 1 calc . . R

H5B H 0.6190 0.0168 0.4178 0.030 Uiso 1 calc . . R

C18 C 0.0440(3) -0.05978(12) 0.03270(9) 0.0316(3) Uani 1 d . . .

H18A H 0.0435 -0.1154 -0.0203 0.047 Uiso 1 calc . . GR

H18B H 0.0474 0.0173 0.0058 0.047 Uiso 1 calc . . GR

H18C H -0.1182 -0.0695 0.0616 0.047 Uiso 1 calc . . GR

C4 C 0.2360(2) 0.08723(12) 0.39228(8) 0.0268(3) Uani 1 d . . .

H4A H 0.0906 0.0854 0.3338 0.032 Uiso 1 calc . . R

H4B H 0.1905 0.0309 0.4402 0.032 Uiso 1 calc . . R

C15 C 0.1290(2) 0.57493(10) 0.27746(9) 0.0247(3) Uani 1 d . . .

C6 C 0.4560(2) -0.03674(11) 0.27614(9) 0.0265(3) Uani 1 d . . .

H6A H 0.6328 -0.0666 0.2668 0.032 Uiso 1 calc . . R

H6B H 0.3499 -0.1014 0.2948 0.032 Uiso 1 calc . . R

C17 C 0.4610(3) 0.22269(14) 0.52803(9) 0.0348(3) Uani 1 d . . .

H17A H 0.6370 0.2104 0.5093 0.052 Uiso 1 calc . . GR

H17B H 0.4364 0.1685 0.5797 0.052 Uiso 1 calc . . GR

H17C H 0.4512 0.3005 0.5527 0.052 Uiso 1 calc . . GR

C13 C 0.3749(3) 0.63107(10) 0.15233(9) 0.0254(3) Uani 1 d . . .

loop_

_atom_site_aniso_label

_atom_site_aniso_U_11

_atom_site_aniso_U_22

_atom_site_aniso_U_33

_atom_site_aniso_U_23

_atom_site_aniso_U_13

_atom_site_aniso_U_12

O5 0.0199(4) 0.0212(4) 0.0345(4) 0.0013(3) 0.0009(3) 0.0034(3)

O1 0.0311(5) 0.0341(5) 0.0406(5) -0.0127(4) 0.0147(4) 0.0052(4)

O6 0.0520(6) 0.0168(4) 0.0366(5) 0.0020(4) 0.0155(4) 0.0056(4)

O3 0.0305(5) 0.0301(5) 0.0223(4) 0.0044(4) 0.0119(3) 0.0046(4)

O4 0.0328(4) 0.0171(4) 0.0268(4) -0.0031(3) 0.0015(3) 0.0022(3)

O2 0.0416(5) 0.0390(5) 0.0387(5) -0.0023(4) 0.0240(4) 0.0051(4)

C11 0.0182(5) 0.0182(5) 0.0233(5) -0.0034(4) 0.0027(4) -0.0004(4)

C16 0.0188(5) 0.0212(5) 0.0226(5) -0.0058(4) 0.0034(4) -0.0004(4)

C1 0.0208(5) 0.0287(6) 0.0229(5) -0.0057(4) 0.0055(4) -0.0016(5)

C9 0.0213(5) 0.0172(5) 0.0157(5) 0.0027(4) 0.0055(3) 0.0024(4)

C14 0.0287(5) 0.0202(5) 0.0327(6) -0.0088(5) 0.0011(4) 0.0054(5)

C8 0.0231(5) 0.0168(5) 0.0238(5) -0.0006(4) 0.0057(4) 0.0028(4)

C12 0.0245(5) 0.0177(5) 0.0246(5) -0.0035(4) 0.0061(4) 0.0018(4)

C7 0.0234(5) 0.0151(5) 0.0224(5) -0.0009(4) 0.0027(4) 0.0012(4)

C10 0.0190(5) 0.0175(5) 0.0242(5) -0.0001(4) 0.0073(4) 0.0031(4)

C3 0.0273(5) 0.0360(7) 0.0179(5) 0.0011(5) 0.0079(4) -0.0041(5)

C5 0.0256(5) 0.0264(6) 0.0215(5) 0.0020(5) -0.0004(4) -0.0007(4)

C18 0.0323(6) 0.0323(6) 0.0281(6) -0.0077(5) -0.0009(5) -0.0024(5)

C4 0.0263(5) 0.0323(6) 0.0221(5) 0.0010(5) 0.0047(4) -0.0073(5)

C15 0.0215(5) 0.0251(6) 0.0269(5) -0.0100(5) 0.0026(4) 0.0018(5)

C6 0.0305(6) 0.0222(6) 0.0249(6) 0.0047(4) -0.0003(4) 0.0021(5)

C17 0.0361(7) 0.0482(9) 0.0198(5) -0.0012(5) 0.0039(4) -0.0096(6)

C13 0.0317(6) 0.0185(5) 0.0246(5) -0.0023(4) 0.0013(4) 0.0015(4)

_geom_special_details

;

All esds (except the esd in the dihedral angle between two l.s. planes)
are estimated using the full covariance matrix. The cell esds are taken
into account individually in the estimation of esds in distances, angles
and torsion angles; correlations between esds in cell parameters are only
used when they are defined by crystal symmetry. An approximate (isotropic)
treatment of cell esds is used for estimating esds involving l.s. planes.

;

loop_

_geom_bond_atom_site_label_1

_geom_bond_atom_site_label_2

_geom_bond_distance

_geom_bond_site_symmetry_2

_geom_bond_publ_flag
O5 C9 1.2133(14) . ?
O1 C15 1.3485(15) . ?
O6 C13 1.3535(16) . ?
O3 C1 1.3290(17) . ?
O3 C3 1.4658(15) . ?
O4 C7 1.4362(13) . ?
O4 C18 1.4326(14) . ?
O2 C1 1.2282(15) . ?
C11 C16 1.4273(16) . ?
C11 C12 1.3749(17) . ?
C11 C10 1.5108(15) . ?
C16 C1 1.4699(17) . ?
C16 C15 1.4230(16) . ?
C9 C8 1.5207(14) . ?
C9 C10 1.5096(15) . ?
C14 C15 1.3830(19) . ?
C14 C13 1.3821(19) . ?
C8 C7 1.5333(16) . ?
C12 C13 1.4009(16) . ?
C7 C6 1.5223(15) . ?
C3 C4 1.5245(19) . ?
C3 C17 1.5169(17) . ?
C5 C4 1.5342(18) . ?
C5 C6 1.5325(16) . ?
loop_
_geom_angle_atom_site_label_1
_geom_angle_atom_site_label_2
_geom_angle_atom_site_label_3
_geom_angle

_geom_angle_site_symmetry_1
_geom_angle_site_symmetry_3
_geom_angle_publ_flag
C1 O3 C3 118.15(9) . . ?
C18 O4 C7 112.08(9) . . ?
C16 C11 C10 124.20(10) . . ?
C12 C11 C16 119.74(10) . . ?
C12 C11 C10 116.07(10) . . ?
C11 C16 C1 125.44(11) . . ?
C15 C16 C11 117.23(11) . . ?
C15 C16 C1 117.30(10) . . ?
O3 C1 C16 115.42(9) . . ?
O2 C1 O3 121.34(12) . . ?
O2 C1 C16 123.22(12) . . ?
O5 C9 C8 119.90(10) . . ?
O5 C9 C10 123.22(10) . . ?
C10 C9 C8 116.84(9) . . ?
C13 C14 C15 119.63(11) . . ?
C9 C8 C7 112.66(9) . . ?
C11 C12 C13 121.63(11) . . ?
O4 C7 C8 110.82(9) . . ?
O4 C7 C6 107.81(9) . . ?
C6 C7 C8 113.06(9) . . ?
C9 C10 C11 115.52(9) . . ?
O3 C3 C4 105.52(9) . . ?
O3 C3 C17 108.00(11) . . ?
C17 C3 C4 114.90(12) . . ?
C6 C5 C4 113.13(9) . . ?
C3 C4 C5 115.07(10) . . ?
O1 C15 C16 122.14(12) . . ?

O1 C15 C14 115.98(11) . . ?

C14 C15 C16 121.86(11) . . ?

C7 C6 C5 113.07(10) . . ?

O6 C13 C14 123.37(11) . . ?

O6 C13 C12 116.91(11) . . ?

C14 C13 C12 119.71(12) . . ?

_olex2_submission_special_instructions 'No special instructions were received'

7. CheckCIFPLATON report of compound 10

You have not supplied any structure factors. As a result the full set of tests cannot be run.

THIS REPORT IS FOR GUIDANCE ONLY. IF USED AS PART OF A REVIEW PROCEDURE FOR PUBLICATION, IT SHOULD NOT REPLACE THE EXPERTISE OF AN EXPERIENCED CRYSTALLOGRAPHIC REFEREE.

No syntax errors found. CIF dictionary Interpreting this report

Datablock: 574-4-4

```
Bond precision:  C-C = 0.0017 A          Wavelength=1.54184

Cell:          a=5.05804(7)   b=11.74745(14)      c=13.77837(18)
               alpha=90       beta=100.1922(13)    gamma=90
Temperature:   150 K

                Calculated                  Reported
Volume          805.779(18)                 805.778(18)
Space group     P 21                        P 1 21 1
Hall group      P 2yb                       P 2yb
Moiety formula  C17 H22 O6                  C17 H22 O6
Sum formula     C17 H22 O6                  C17 H22 O6
Mr              322.35                      322.35
Dx,g cm-3       1.329                       1.329
Z               2                           2
Mu (mm-1)       0.836                       0.836
F000            344.0                       344.0
F000'           345.17
h,k,lmax        6,14,17                     6,14,17
Nref            3359[ 1766]                 3141
Tmin,Tmax                                   0.547,1.000
Tmin'

Correction method= # Reported T Limits: Tmin=0.547 Tmax=1.000
AbsCorr = MULTI-SCAN

Data completeness= 1.78/0.94       Theta(max)= 75.460

R(reflections)= 0.0333( 3123)      wR2(reflections)= 0.1007( 3141)

S = 0.971                 Npar= 212
```

The following ALERTS were generated. Each ALERT has the format
test-name_ALERT_alert-type_alert-level.
Click on the hyperlinks for more details of the test.

Alert level C

```
CRYSC01_ALERT_1_C No recognised colour has been given for crystal colour.
PLAT029_ALERT_3_C _diffrn_measured_fraction_theta_full Low .......      0.971 Note
PLAT053_ALERT_1_C Minimum Crystal Dimension Missing (or Error) ...     Please Check
PLAT054_ALERT_1_C Medium  Crystal Dimension Missing (or Error) ...     Please Check
PLAT055_ALERT_1_C Maximum Crystal Dimension Missing (or Error) ...     Please Check
```

Alert level G

```
PLAT005_ALERT_5_G No Embedded Refinement Details found in the CIF      Please Do !
PLAT007_ALERT_5_G Number of Unrefined Donor-H Atoms ..............          2 Report
PLAT791_ALERT_4_G The Model has Chirality at C3      (Chiral SPGR)          R Verify
PLAT791_ALERT_4_G The Model has Chirality at C7      (Chiral SPGR)          S Verify
```

```
   0 ALERT level A = Most likely a serious problem - resolve or explain
   0 ALERT level B = A potentially serious problem, consider carefully
   5 ALERT level C = Check. Ensure it is not caused by an omission or oversight
   4 ALERT level G = General information/check it is not something unexpected

   4 ALERT type 1 CIF construction/syntax error, inconsistent or missing data
   0 ALERT type 2 Indicator that the structure model may be wrong or deficient
   1 ALERT type 3 Indicator that the structure quality may be low
   2 ALERT type 4 Improvement, methodology, query or suggestion
   2 ALERT type 5 Informative message, check
```

Validation response form

Please find below a validation response form (VRF) that can be filled in and pasted into your CIF.

```
# start Validation Reply Form
_vrf_CRYSC01_574-4-4
;
PROBLEM: No recognised colour has been given for crystal colour.
RESPONSE: ...
;
_vrf_PLAT029_574-4-4
;
PROBLEM: _diffrn_measured_fraction_theta_full Low .......      0.971 Note
RESPONSE: ...
;
_vrf_PLAT053_574-4-4
;
PROBLEM: Minimum Crystal Dimension Missing (or Error) ...     Please Check
RESPONSE: ...
;
_vrf_PLAT054_574-4-4
;
PROBLEM: Medium  Crystal Dimension Missing (or Error) ...     Please Check
RESPONSE: ...
;
_vrf_PLAT055_574-4-4
;
PROBLEM: Maximum Crystal Dimension Missing (or Error) ...     Please Check
RESPONSE: ...
;
# end Validation Reply Form
```

It is advisable to attempt to resolve as many as possible of the alerts in all categories. Often the minor alerts point to easily fixed oversights, errors and omissions in your CIF or refinement strategy, so attention to these fine details can be worthwhile. In order to resolve some of the more serious problems it may be necessary to carry out additional measurements or structure refinements. However, the purpose of your study may justify the reported deviations and the more serious of these should normally be commented upon in the discussion or experimental section of a paper or in the "special_details" fields of the CIF. checkCIF was carefully designed to identify outliers and unusual parameters, but every test has its limitations and alerts that are not important in a particular case may appear. Conversely, the absence of alerts does not guarantee there are no aspects of the results needing attention. It is up to the individual to critically assess their own results and, if necessary, seek expert advice.

Publication of your CIF in IUCr journals

A basic structural check has been run on your CIF. These basic checks will be run on all CIFs submitted for publication in IUCr journals (*Acta Crystallographica*, *Journal of Applied Crystallography*, *Journal of Synchrotron Radiation*); however, if you intend to submit to *Acta Crystallographica Section C* or *E*, you should make sure that full publication checks are run on the final version of your CIF prior to submission.

Publication of your CIF in other journals

Please refer to the *Notes for Authors* of the relevant journal for any special instructions relating to CIF submission.

PLATON version of 19/11/2015; check.def file version of 17/11/2015

Datablock 574-4-4 - ellipsoid plot

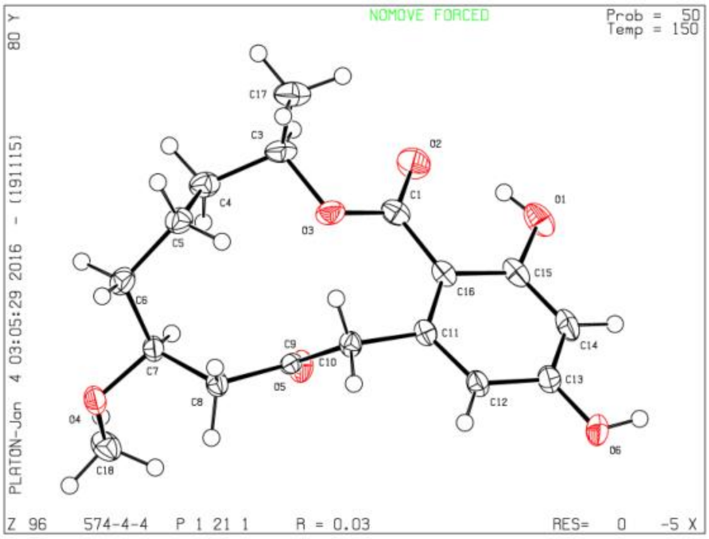

Supplement: Supplementary Information [file srep27396-s1.pdf]
